# Hidden diversity in Europe: new species and a revised taxonomy of the subgenus *Leptoconops* (*Leptoconops*) (Diptera, Ceratopogonidae)

**DOI:** 10.3897/zookeys.1279.186179

**Published:** 2026-05-08

**Authors:** Dumitru Ionut Paun-Tanase, Mikel Alexander González, Sergio Magallanes, Giovanni Naro, Sara Epis, Jordi Figuerola

**Affiliations:** 1 Departamento de Biología de la Conservación y Cambio Global, Estación Biológica de Doñana (EBD, CSIC), Avda. Américo Vespucio 26, 41092, Sevilla, Spain CIBER de Epidemiologia y Salud Pública (CIBERESP) Madrid Spain; 2 CIBER de Epidemiologia y Salud Pública (CIBERESP), Av. Monforte de Lemos, 3-5. Pabellón 11. Planta 0, 28029 Madrid, Spain Departamento de Biología de la Conservación y Cambio Global, Estación Biológica de Doñana (EBD, CSIC) Sevilla Spain; 3 Department of Biosciences and Pediatric Clinical Research Center "Romeo and Enrica Invernizzi", University of Milan, Milan, Italy Department of Biosciences and Pediatric Clinical Research Center “Romeo and Enrica Invernizzi”, University of Milan Milan Italy

**Keywords:** Biting flies, biting midges, blood sucking arthropods, diversity, new distribution records, new species, pest, taxonomy

## Abstract

The Ceratopogonidae (Diptera, Nematocera) is a highly diverse family of insects inhabiting a wide range of habitat types that is only absent from polar regions and remote islands. Despite this broad diversity, scientific knowledge of this family remains incomplete, including the genus *Leptoconops* Skuse, 1889. Although several *Leptoconops* species have been reported from Europe, no detailed study has yet been conducted to resolve the taxonomy of this genus and clarify species occurrence. Here, the first comprehensive study of *Leptoconops* diversity in southwest Spain is provided based on extensive sampling conducted in 2023–2024. In total, eight *Leptoconops* species were collected using carbon-dioxide suction traps: Leptoconops (Leptoconops) noei Clastrier & Coluzzi, 1973, Leptoconops (Leptoconops) irritans Noé, 1905, and four new species: Leptoconops (Leptoconops) nigrithorax González & Tanase, **sp. nov**., Leptoconops (Leptoconops) triangularis González & Tanase, sp. nov., Leptoconops (Leptoconops) pseudoirritans González & Tanase, **sp. nov**., and Leptoconops (Leptoconops) communis González & Tanase, **sp. nov**. Additionally, Leptoconops (Leptoconops) bidentatus Gutsevich, 1960, was recorded for the first time from the Iberian Peninsula. One species of the subgenus *Holoconops*: Leptoconops (Holoconops) cf. *kerteszi* Kieffer, 1908, was also identified. Molecular support for the morphological identification is provided using COX1 barcode sequences. A brief review of *Leptoconops* species occurrence in Europe is also included, with detailed morphological descriptions supported by high-quality images of the newly described species, and a complete identification key for dry-preserved and slide-mounted adult females of the subgenus *L.* (*Leptoconops*).

## Abstract (es)

La familia Ceratopogonidae constituye un grupo diverso de dípteros, que habita en una amplia variedad de hábitats, estando ausente únicamente de las regiones polares e islas remotas. Sin embargo, algunos géneros de esta familia, como *Leptoconops* Skuse, 1889 han sido escasamente estudiados, particularmente en Europa, donde el conocimiento sobre estos jejenes sigue siendo muy incompleto. En consecuencia, aún falta información relevante sobre su taxonomía, biología y ecología. Este estudio tiene como objetivo dilucidar la diversidad de *Leptoconops* en el suroeste de España, basado en un muestreo intensivo realizado en 2023 y 2024. Se recolectaron ejemplares de ocho especies distintas utilizando trampas cebadas con CO_2_: Leptoconops (Leptoconops) noei Clastrier & Coluzzi, 1973, Leptoconops (Leptoconops) irritans Noé, 1905, y cuatro nuevos taxones: Leptoconops (Leptoconops) nigrithorax González & Tanase, **sp. nov**., Leptoconops (Leptoconops) triangularis González & Tanase, **sp. nov**., Leptoconops (Leptoconops) pseudoirritans González & Tanase, **sp. nov**. y Leptoconops (Leptoconops) communis González & Tanase, **sp. nov**. Asimismo, Leptoconops (Leptoconops) bidentatus Gutsevich, 1960 se cita por primera vez en la Península Ibérica. También se identificó una especie del subgénero *Holoconops*: Leptoconops (Holoconops) cf. *kerteszi* Kieffer, 1908. Esta información morfológica se respalda por secuencias del gen COX1. Finalmente, se incluye una breve revisión de las especies de *Leptoconops* en Europa, descripciones morfológicas detalladas respaldadas por imágenes de alta calidad de las especies recién descritas y una clave de identificación exhaustiva para hembras adultas del subgénero *L.* (*Leptoconops*) en Europa, aplicable tanto a especímenes conservados en seco como a ejemplares montados.

## Introduction

The Ceratopogonidae (Diptera, Nematocera) comprises a highly diverse group of minute dipterans, with more than 6,300 described extant species placed in 107 genera, as well as probably many thousands of additional species yet to be recognized worldwide, based solely on morphological diagnosis (Borkent et al. 2024, 2025). In Europe, approximately 590 species belonging to 27 genera have been documented (Jong et al. 2014; Borkent and Dominiak 2020). The adult females of four genera [*Austroconops* Wirth & Lee, 1959, *Leptoconops* Skuse, 1889, *Forcipomyia* Meigen,1818 (subgenus *F.* (*Lasiohelea*) Kieffer, 1921), and *Culicoides* Latreille, 1809] are hematophagous and need to feed on vertebrate blood to complete egg development (Kettle 1962; Borkent and Spinelli 2007; Mullen and Murphree 2019). By contrast, adult males are pollinators that depend on nectar for their development, although some females of certain species also visit flowers for pollen to satisfy their energetic requirements (Wiesenborn 2003; Chua et al. 2020). Among the blood-sucking genera, *Culicoides* is the most diverse and significant genus, with 1,373 described species (Borkent et al. 2025), including numerous species of medical and veterinary importance implicated in the transmission of pathogens such as viruses, protozoans, and filarial nematodes to humans and animals (Mullen and Murphree 2019; Borkent and Dominiak 2020). Albeit less studied, other genera such as *Leptoconops* are notorious pests that severely affect human and animal activity including tourism and certain outdoor activities (Bettini and Finizio 1968; Fausto et al. 2007; Borkent and Dominiak 2020). The genus *Leptoconops* includes a total of 174 species distributed worldwide (Borkent and Dominiak 2020; Borkent et al. 2025; Ghosh et al. 2025).

Adult female *Leptoconops*, commonly known as “black gnats” or “biting midges”, can be morphologically distinguished from *Culicoides* in the Palearctic Region by their milky-white wings, the absence of the r-m cross-vein, with 11 or 12 flagellomeres and typically markedly elongate cerci (Carter 1921; Wirth et al. 1974). Although there are only a moderate number of *Leptoconops* species, these midges are widely distributed in tropical and subtropical regions and are infamous for their aggressive biting behaviour. In recent years, this genus has gained attention in biostratigraphy, paleoecology, and amber fossil studies (Szadziewski 2016). Unlike *Culicoides*, *Leptoconops* species are diurnal. In the Mediterranean region, they are most active in the early hours of the day when temperatures typically range between 25 and 30 °C (Carrieri et al. 2011). While many of these species require a blood meal for oviposition, some are autogenous and are capable of reproducing without (Linley 1983). Despite being stronger flyers than *Culicoides*, *Leptoconops* usually remain close to their breeding sites and are highly sensitive to wind, which can hinder their active dispersal (Carrieri et al. 2011).

Thirty-five species of *Leptoconops* have been documented in the Western Palearctic region (Borkent and Dominiak 2020). The knowledge of *Leptoconops* in Europe is largely restricted to Italy and France, where their biting activity poses significant public health concerns. In Tuscany and Lazio, and in the coastal regions of Languedoc and Camargue, significant economic losses in the tourism sector have been associated with the nuisance of their bites (Bettini and Finizio 1968; Belardinelli et al. 2010). In Spain, *Leptoconops* has largely been neglected, with only three extant species officially recorded to date (Delecolle 2002; González et al. 2013, 2025), mainly due to the lack of research in the region.

Several comprehensive faunistic studies focusing on this genus in Europe have been published (Gutsevich 1960; Clastrier 1973; Clastrier and Coluzzi 1973), but current research is limited by the lack of updated taxonomic keys of the *Leptoconops* genus. Accurate species identification represents a fundamental step in any attempt to generate biological and ecological knowledge of vector species. To address this gap, we provide a detailed morphological characterization of the Leptoconops (Leptoconops) species present in southern Spain based on adult female morphology. In addition, we provide the first dichotomous identification key for adult females, applicable to both dry and freshly collected, and slide-mounted specimens, to facilitate the identification of all Leptoconops (Leptoconops) species currently known in Europe.

## Materials and methods

### Material examined

The material analysed was collected in 2023–2024 in the provinces of Seville, Cádiz, and Huelva, in an area of ca 31,500 km^2^. BG-Sentinel traps (BIOGENS, Germany) (BGS) baited with ~1.2 kg of CO_2_ (dry ice) were used to collect the specimens. A total of 951 sampling sites were surveyed during the study period. Further details of the trapping methods and design are provided in González et al. (2024). The holotype and paratypes of the new species have been deposited in the Invertebrate Collection of the Scientific Collection at Estación Biológica de Doñana (**EBD-CSIC**, Sevilla, Spain). Additional paratypes have been deposited in the Entomology Collection of the Museo Nacional de Ciencias Naturales (**MNCN-CSIC**, Madrid, Spain) and in the Natural History Museum (**NHMUK**, London, United Kingdom). All these collections provide access to material upon request (EBD-CSIC: https://icts-donana.csic.es/servicios-instalaciones/colecciones-cientificas; MNCN-CSIC: https://www.mncn.csic.es/es/colecciones/cientificas/entomologia-col; NHMUK: https://www.nhm.ac.uk/our-science/services/collections/entomology.html).

### Morphological identification

Specimens were sorted in Petri dishes by sex, locality, and date. The collected material consisted almost entirely of females. A single male specimen of Leptoconops (Leptoconops) irritans Noé, 1905 was collected in poor condition as noted below. Taxa were initially grouped based on external morphological traits observed under a stereomicroscope, after which selected specimens were prepared in Hoyer’s medium for slide-mounting (7 specimens per taxon). Before mounting, specimens were digested in 10% NaOH for 24 h and then dissected into their respective body parts: head, thorax, abdomen, and legs. Photographed specimens were examined under a ZEISS Stemi 2000 C stereomicroscope equipped with a Leica Flexacam C3 (Leica Microsystems, Spain) and a ZEISS Axiolab 5 microscope equipped with an Axiocam 208 color digital camera (Carl Zeiss Microscopy GmbH, Germany). Identification was based on existing taxonomic keys and the original species descriptions (Harant and Galan 1944; Gutsevich 1960, 1973; Clastrier 1972; Clastrier and Coluzzi 1973).

Measurements were performed using the graphics tools included in ZEISS software. The following structures were measured: (i) body length: distance from the tip of the head to the extreme of the lamella; (ii) head (length × width, anterior view): distance from top of the head to the tormae; (iii) interocular space as the distance between eyes (in number of ommatidia); (iv) palpus (length × width, ventral view); (v) sensory pit area of the third palpal segment; (vi) proboscis (length): distance from the tip of the labrum to the torma; (vii) antenna (length): sum of the lengths of flagellomeres I–XII; (viii) thorax (length × width, dorsal view): length includes the scutellum, the width being measured at the widest part (humeral callus); (ix) wing (length × width): length measured from basal arculus to the wing tip, and width as the distance from the apex of M_4_ to the anterior wing margin; (x) legs (length of all 3 pairs): sum of trochanter, femur, tibia, and I–V tarsomeres; (xi) lamella (length × width): length measured from the base to the apex of the lamella and the width of the base; and (xii) spermatheca (length × width). The following indices were calculated: (i) antennal ratio (AR), defined as the combined length of the four apical flagellomeres (IX–XII) divided by the combined length of the basal flagellomeres (I–VIII); (ii) palpal ratio (PR), defined as the length of the third palpal segment divided by its breadth; (iii) costal ratio (CR), defined as the length of the costa from the arculus divided by the wing length; and (iv) lamella index, defined as the lamellar length divided by its breadth at its base; (v) third palpal index, defined as the palpal length divided by its breadth at the middle.

Between five and seven specimens were measured to calculate mean values. This sample size was considered sufficient to ensure a 95% confidence level with a 5% margin of error. Standard deviations (SD) were also calculated. Body and wing measurements were recorded in millimeters (mm), while all other measurements were expressed in µm. All measurements were taken from slide-mounted specimens (MS), except for body, thorax, and wing length, which were obtained from dry and freshly collected specimens (FS).

Morphological terminology for the species description follows Downes and Wirth (1981) and Borkent (2017, 2024). The terminology for the antennal sensory organs follows Wirth and Navai (1978) and distinguishes between the sensilla chaetica, sensilla trichodea, and sensilla basiconica, also referred to as the blunt-tipped sensilla or hyaline sensory setae (ss).

### Molecular identification

Genomic DNA was extracted from whole bodies (*n* = 30 specimens) using the DNeasy® blood and tissue kit (QIAGEN, Germany). A 658 bp fragment of the COX1 gene was amplified using the universal primers LCO1490 (5'-GGTCAACAAATCATAAAGATATTGG-3') and HCO2198 (5'-TAAACTTCAGGGTGACCAAAAAATCA-3') following Folmer et al. (1994). To improve primer specificity for the *Leptoconops* DNA sequences, the annealing temperature was set to 50 °C, rather than 40 °C, as noted in the original protocol, for 1 min. Amplicons were sequenced in both directions via capillary electrophoresis by the genomics service at the Universidad Complutense de Madrid (Spain). Forward and reverse sequences were aligned using pairwise alignment, followed by the trimming of amplicon ends and primer regions to obtain 658-bp consensus sequences. Sequence editing was carried out using Geneious v. 2025.1.2 (https://www.geneious.com). The resulting sequences were compared using the Basic Local Alignment Search Tool (megablast algorithm) (NCBI BLAST, 2019 https://blast.ncbi.nlm.nih.gov/Blast.cgi). The COX1 sequences were submitted to GenBank (https://www.ncbi.nlm.nih.gov/genbank/) via the BankIt submission portal (https://submit.ncbi.nlm.nih.gov/) for public use under the accession numbers listed in Table 1.

**Table 1.** COX1 sequences of the *Leptoconops* species recorded in our study.

Species	Subgenus	GenBank accession number
*Leptoconops nigrithorax* González & Tanase, sp. nov.	* Leptoconops *	PX112029, PX112030
*Leptoconops triangularis* González & Tanase, sp. nov.	* Leptoconops *	PX253990, PX253991
*Leptoconops pseudoirritans* González & Tanase, sp. nov.	* Leptoconops *	PX290102, PX290103, PX290104, PX290105, PX504285
*Leptoconops communis* González & Tanase, sp. nov.	* Leptoconops *	PX290098, PX290099, PX290100, PX290101, PX501801, PX501802, PX501803, PX501804, PX501805, PX501806
*Leptoconops bidentatus* Gutsevich, 1960	* Leptoconops *	PX058092, PX058093
*Leptoconops noei* Clastrier & Coluzzi, 1973	* Leptoconops *	PX063902, PX063903, PX063904,
*Leptoconops irritans* Noé, 1905	* Leptoconops *	PV617359, PV617360, PV617361
*Leptoconops cf. kerteszi* Kieffer, 1908	* Holoconops *	PV628239, PV628240, PV628241

### Literature review of *Leptoconops* in Europe and surrounding regions

A literature review was conducted to identify relevant studies addressing *Leptoconops* species reported in Europe and surrounding regions (North Africa and the Middle East). The literature search was performed using Web of Science search engine covering the publication period from 1905 to 2025. Search terms used a combination of keywords such as ‘Biting midges’, ‘Ceratopogonidae’, ‘Black gnats’, or ‘*Leptoconops’* in conjunction with ‘*irritans’*, ‘*bidentatus’*, ‘*bezzii’*, ‘*noei’*, ‘*pugnax’*, ‘*gallicus’*, ‘*kertezsi’*, ‘*borealis’*, ‘*pavlovskyi’*, ‘*lisbonnei’*, ‘*acer’*, and other terms such as ‘Europe’, ‘presence’, ‘synonyms’, ‘North Africa’, ‘Middle East, ‘Central Europe’. The consulted bibliography was Gutsevich (1960), Clastrier (1972, 1973), Soós and Papp (1988), Delecolle (2002), González et al. (2013, 2025), Borkent and Dominiak (2020), and Borkent et al. (2025).

## Results

Our study identified eight *Leptoconops* species present in southwest Spain. Within the subgenus *L.* (*Leptoconops*), seven species were detected, including one new record and four new species: Leptoconops (Leptoconops) noei Clastrier & Coluzzi, 1973, L. (L.) bidentatus Gutsevich, 1960, L. (L.) irritans Noé, 1905, L. (L.) pseudoirritans González & Tanase, sp. nov., L. (L.) triangularis González & Tanase, sp. nov., L. (L.) nigrithorax González & Tanase, sp. nov. and L. (L.) communis González & Tanase, sp. nov.

A single species was also assigned to the subgenus *Holoconops*: Leptoconops (Holoconops) cf. kerteszi Kieffer, 1908. This discovery of four new species and a new record increase the known diversity of this genus to 15 species in Europe and nine in Spain (although the existence of *Leptoconops bezzii* Noé, 1905 requires confirmation).

We provide a revised key for the European species of the subgenus *L.* (*Leptoconops*) applicable to both dry-fresh and slide-mounted specimens (Figs 1, 3, 5, 7, Suppl. material 1: figs S1–S25).

**Figure 1.**
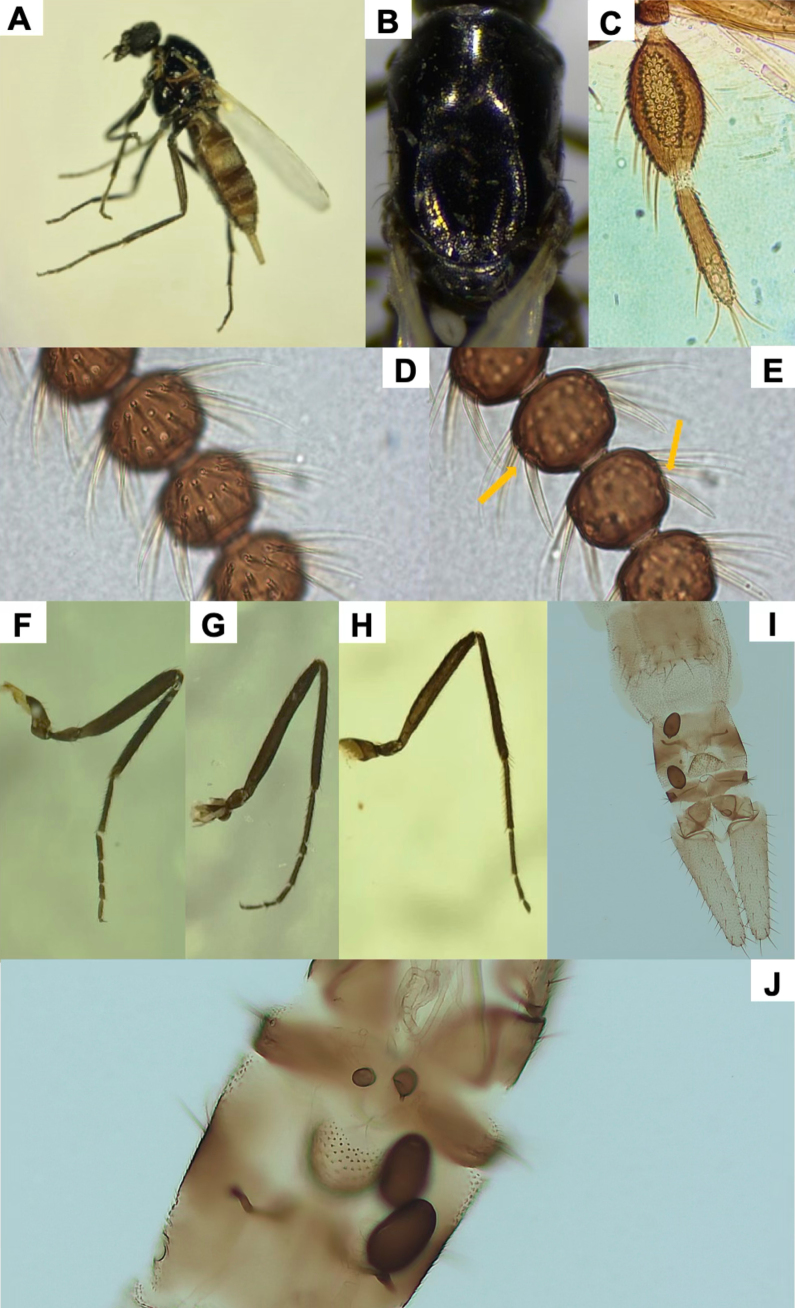
*Leptoconops nigrithorax* sp. nov. voucher (from holotype and paratypes). **A**. Habitus, lateral view (antennae missing) (FS); **B**. Thorax, dorsal view (FS); **C**. Palpus, ventral view (MS); **D**. Sensilla trichodea on proximal flagellomeres (MS); E. Hyaline sensory setae (indicated by yellow arrows) on distal flagellomeres (MS); **F**. Foreleg; **G**. Midleg; **H**. Hindleg (FS); **I**. Apical portion of the abdomen, ventral view; genitalia (MS); **J**. Main and accessory spermathecae (MS).

**Figure 2.**
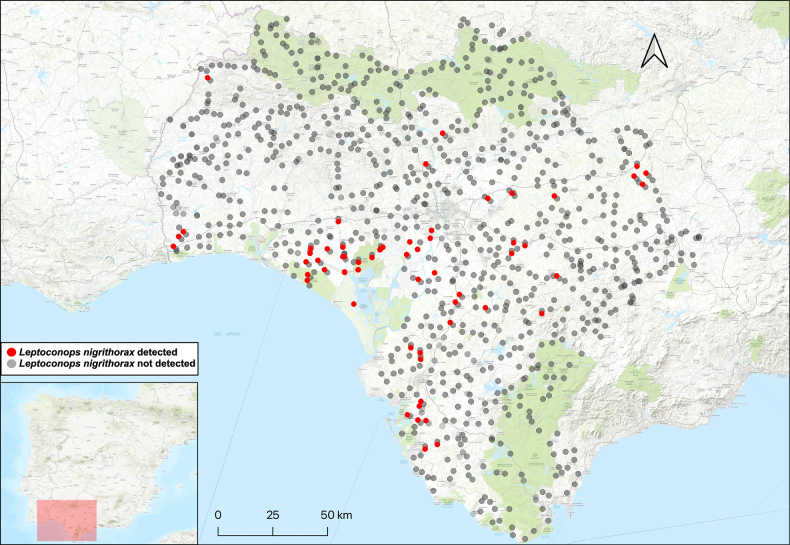
*Leptoconops nigrithorax* sp. nov. distribution in the study area, shown in red. The various attempted sampling sites are indicated in grey.

**Figure 3.**
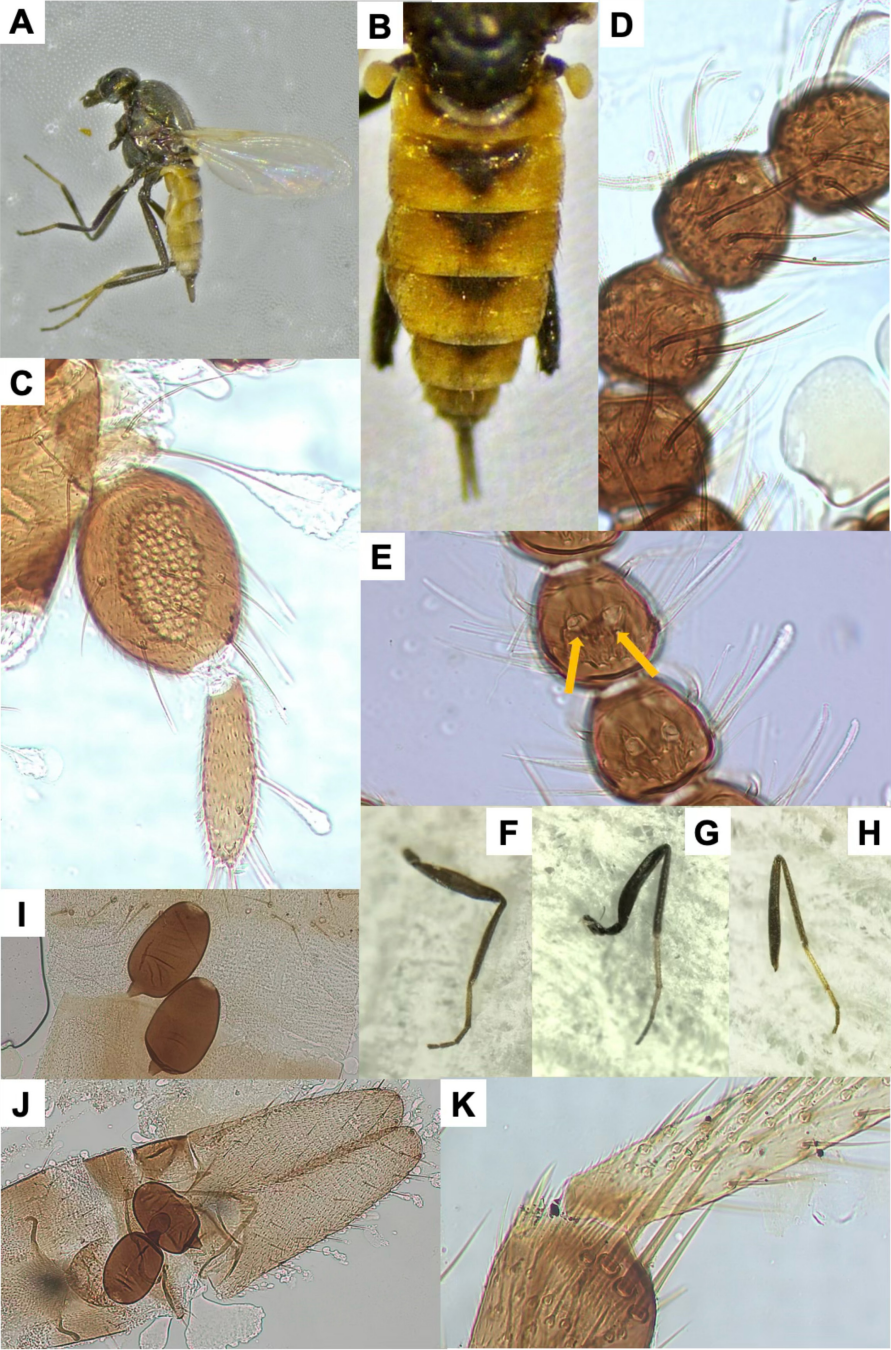
*Leptoconops triangularis* sp. nov. voucher (from holotype and paratypes). **A**. Habitus, lateral view (antennae and forelegs missing) (FS); B. Abdomen, dorsal view (FS); **C**. Palpus, ventral view (MS); **D**. Sensilla chaetica on proximal flagellomeres (MS); **E**. Hyaline sensory setae (indicated by yellow arrows) on distal flagellomeres (MS); **F**. Foreleg; **G**. Midleg; **H**. Hindleg (FS); **I**. Main spermathecae (MS); **J**. Apical portion of the abdomen, ventral view; genitalia and main spermathecae (MS); **K**. Tibial comb (MS).

**Figure 4.**
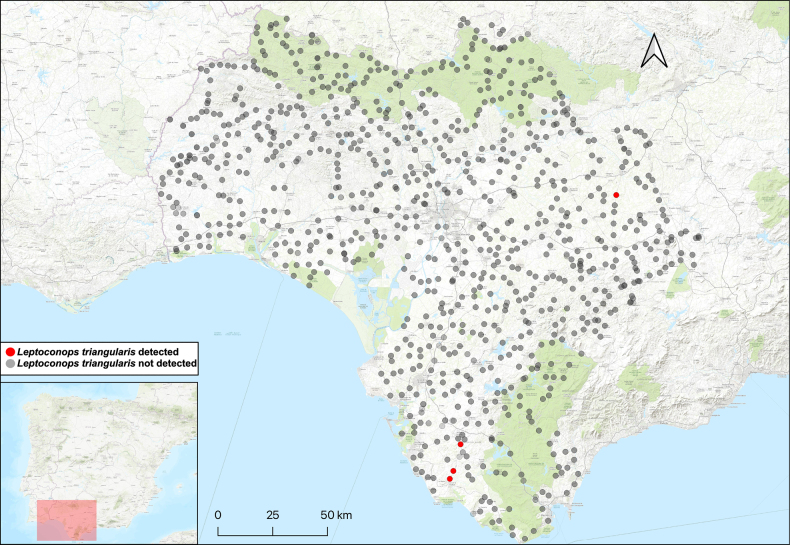
*Leptoconops triangularis* sp. nov. distribution in the study area, shown in red. The various attempted sampling sites are indicated in grey.

**Figure 5.**
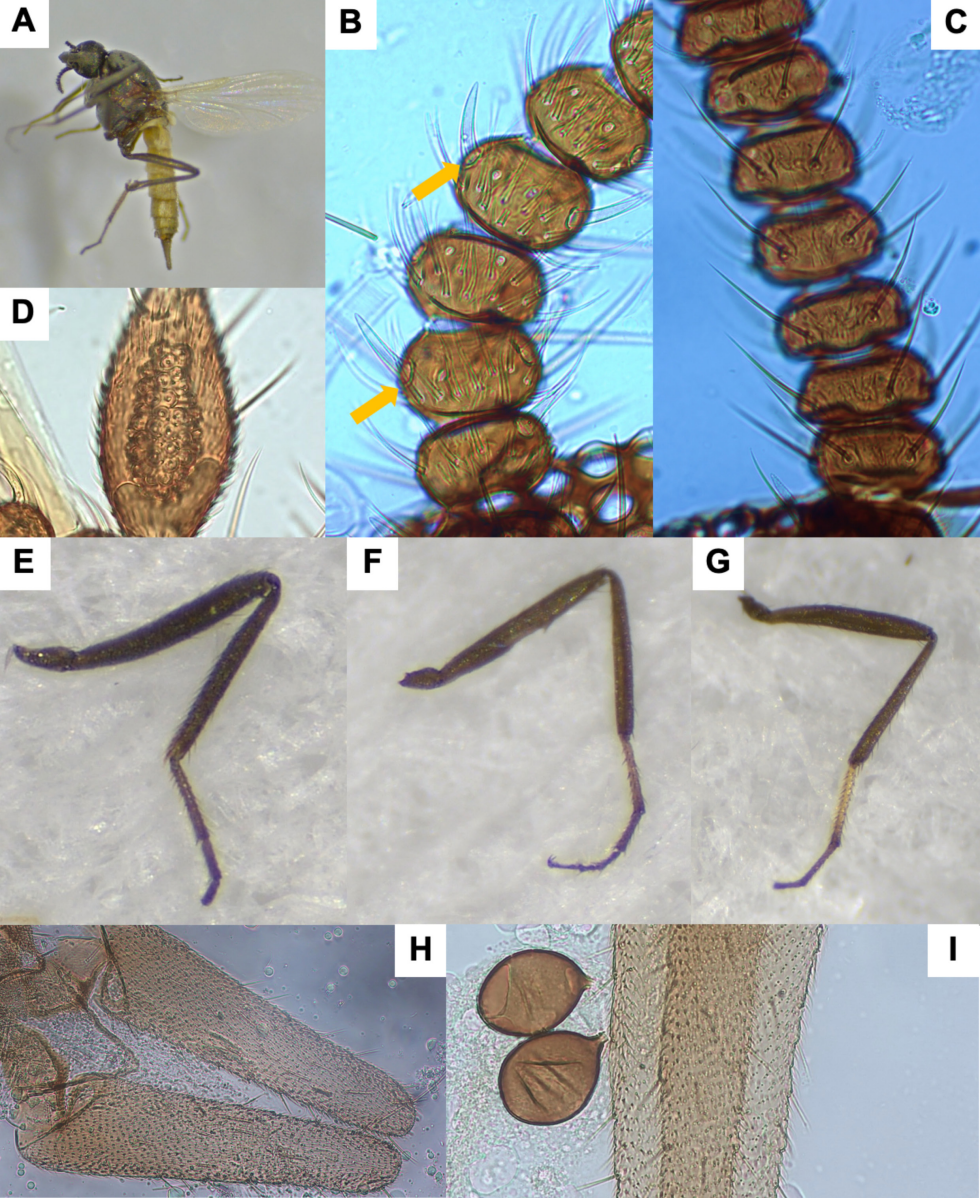
*Leptoconops pseudoirritans* sp. nov. voucher (from holotype and paratypes). **A**. Habitus, lateral view (FS); **B**. Hyaline sensory setae (pointed by yellow arrows) and sensilla trichodea on proximal flagellomeres (MS); **C**. Sensilla chaetica on proximal flagellomeres (MS); **D**. Third palp segment, ventral view (MS); **E**. Foreleg; **F**. Midleg; **G**. Hindleg (FS); **H**. Apical portion of the abdomen, ventral view; genitalia; **I**. Main spermathecae (MS).

**Figure 6.**
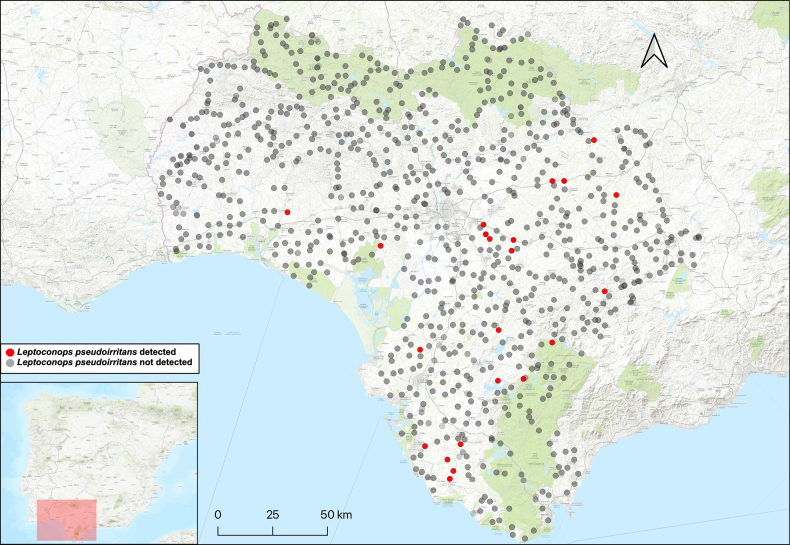
*Leptoconops pseudoirritans* sp. nov. distribution in the study area, shown in red. The various attempted sampling sites are indicated in grey.

**Figure 7.**
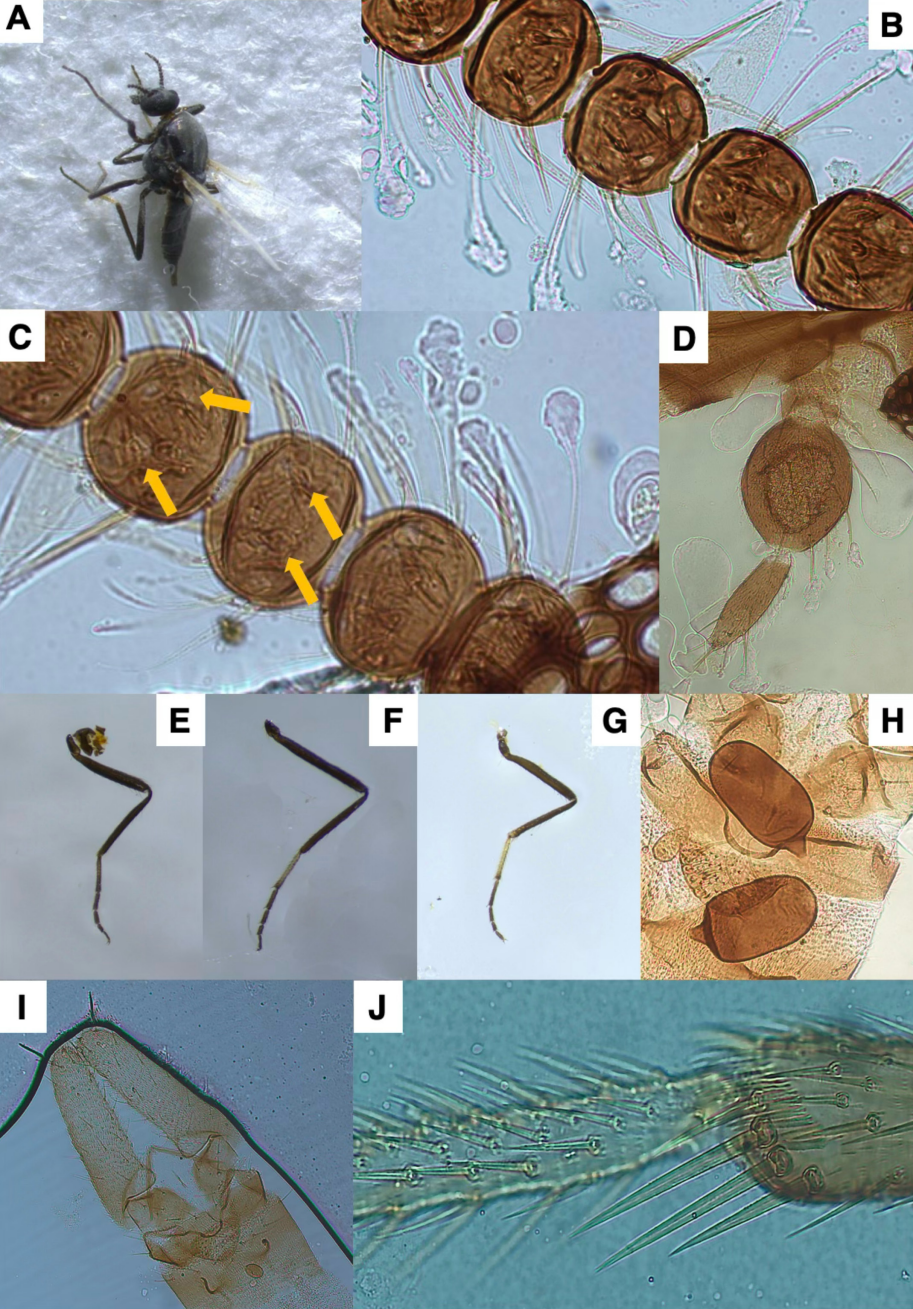
*Leptoconops communis* sp. nov. voucher (from holotype and paratypes). **A**. Habitus, lateral view (FS); **B**. Sensilla trichodea and sensilla chaetica on distal flagellomeres (MS); **C**. Hyaline sensory setae (indicated by yellow arrows) on proximal flagellomeres (MS); **D**. Palpus, ventral view (MS); **E**. Foreleg; **F**. Midleg; **G**. Hindleg (FS); **H**. Main spermathecae (MS); **I**. Apical portion of the abdomen, ventral view; genitalia (MS); **J**. Tibial comb (MS).

### Key to species of the subgenus Leptoconops (Leptoconops) of Europe (based on dry-fresh specimens at 40–80 × magnification)

Note that our key includes all the *L.* (*Leptoconops*) species recorded in Europe except *Leptoconops lisbonnei* Harant & Galan, 1944, for which only a male was described in France (Harant and Galan 1944).

#### Female

**Table:** 

1	Legs uniformly colored; thorax shiny and polished (dark brown or black). Humeral pits absent or inconspicuous; post-scutellar pits present	**2**
–	Legs bicolored; thorax pruinose, dull or polished (grey or greenish tones). Humeral and post-scutellar pits present	**3**
2	Legs dark brown (Fig. 1F–H); thorax and pleuron shiny black (Fig. 1B). Abdomen dark brown. Palpus uniformly colored	***L. nigrithorax* sp. nov**.
–	Legs pale brown (Fig. 10A); thorax and pleuron shiny brown (Fig. 10B). Abdomen yellow or pale brown (Fig. 10A). Palpus bicolored	***L. bidentatus* Gutsevich, 1960**
3	Abdomen pale cream, pale grey-yellow, sepia-tinted or isabelline; proboscis variable	**4**
–	Abdomen different (dark, brownish, or greyish); proboscis shorter than head	**6**
4	Proboscis distinctly longer than head; palpus elongated with last segment large and slender; abdominal terga without unmarked or with blurred black spots	**5**
–	Proboscis as long or slightly shorter than head; palpus markedly swollen, last segment of normal size and length (Fig. 3C); abdominal terga with 3 or 4 distinctive black triangular patches (Fig. 3B)	***L. triangularis* sp. nov**.
5	Large species (> 1.9 mm); abdomen > 1.6 mm; hindlegs > 1.8 mm; proboscis 1.2–1.4 × longer than the head (Fig. 11A–D)	***L. irritans* Noé, 1905**
–	Small species (< 1.7 mm); abdomen ~ 1 mm; hindlegs < 1.8 mm; proboscis 1.3–1.4 × longer than the head (Fig. 5A)	***L. pseudoirritans* sp. nov**.
6	Small species (< 1.7 mm); abdomen pale brownish, darker on tergites. Forelegs with brown femur and tibia, but tarsomeres are mostly pale except the apical part (Fig. 12N); the other tarsomeres evenly brown	***L. noei* Clastrier & Coluzzi, 1973**
–	Abdomen dark brown; forelegs evenly brown (Fig. 7E)	**7**
7	Medium-sized species (< 1.8 mm). Usually four humeral pits (Suppl. material 1: fig. S22B)	***L. communis* sp. nov**.
–	Large species (> 1.9 mm). Usually five humeral pits (Fig. 13O)	***L. bezzii* Noé, 1905**

### Key to species of the subgenus Leptoconops (Leptoconops) of Europe (based on slide-mounted specimens at 100–400 × magnification)

Note that our key includes all the *L.* (*Leptoconops*) species recorded in Europe except *Leptoconops lisbonnei* Harant & Galan, 1944, for which only a male was described in France (Harant and Galan 1944).

**Figure 8.**
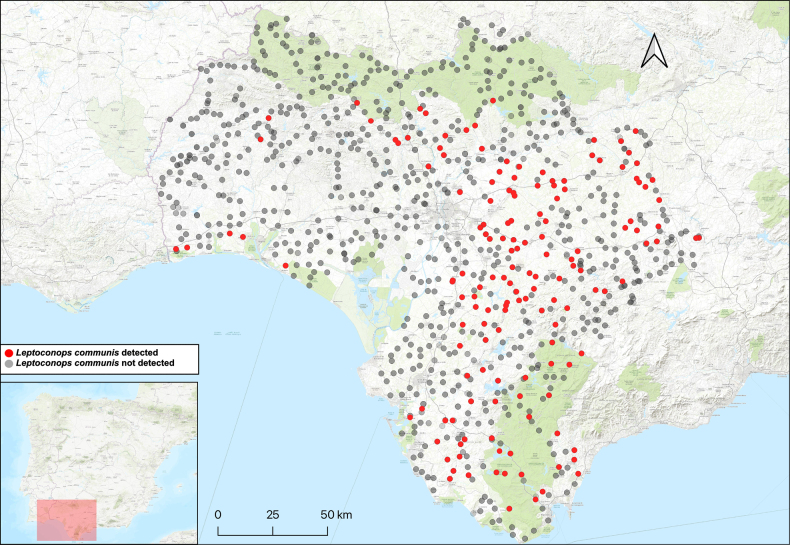
*Leptoconops communis* sp. nov. distribution in the study area, shown in red. The various attempted sampling sites are indicated in grey.

**Figure 9.**
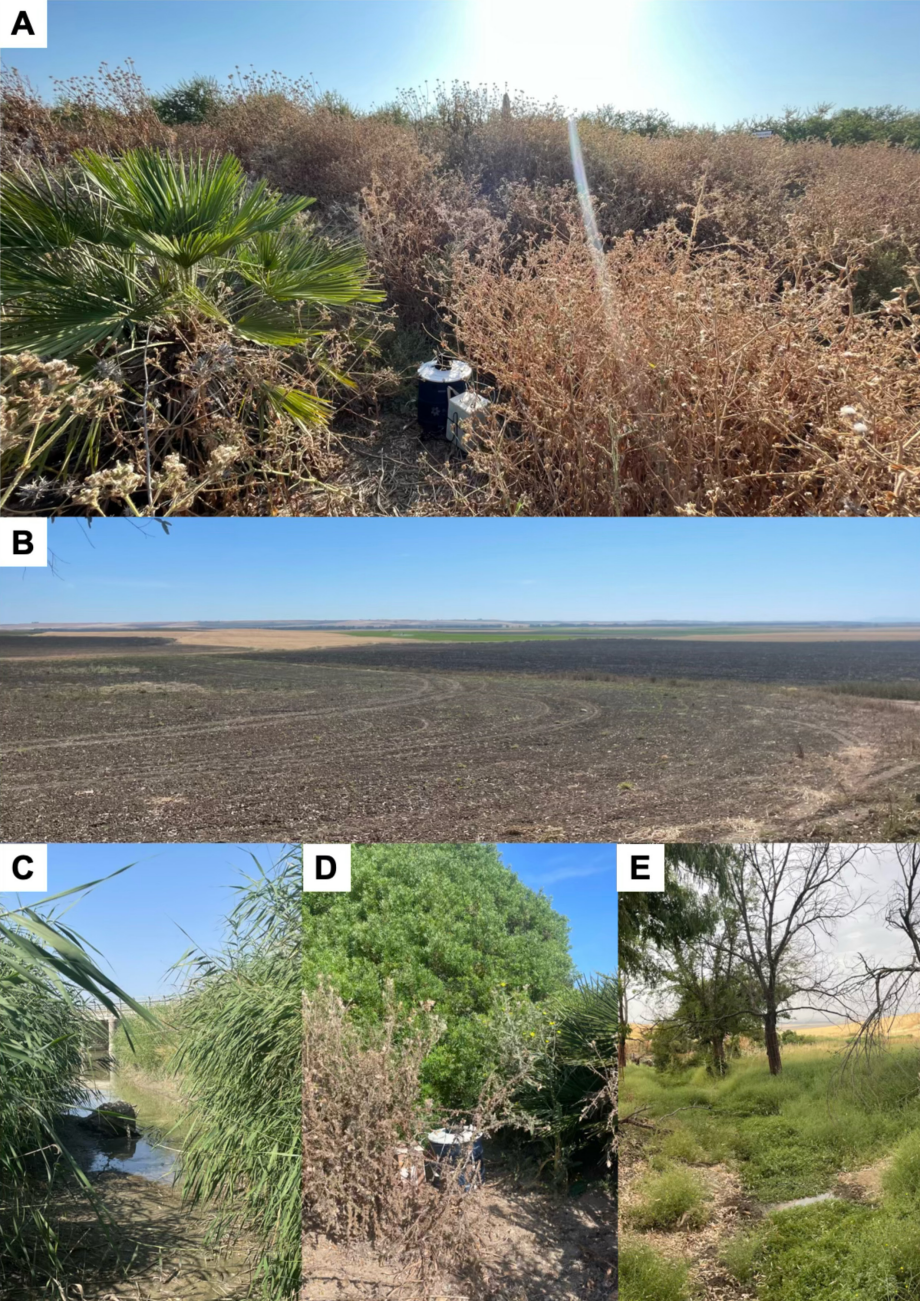
Examples of habitats where the Leptoconops species recorded in this study were recorded: **A**. Mediterranean scrub dominated by *Chamaerops humilis*; **B**. arable cropland margins; **C**. riverside vegetation; **D**. *Pistachia lentiscus* scrub; **E**. transitional woodland scrub habitats.

**Figure 10.**
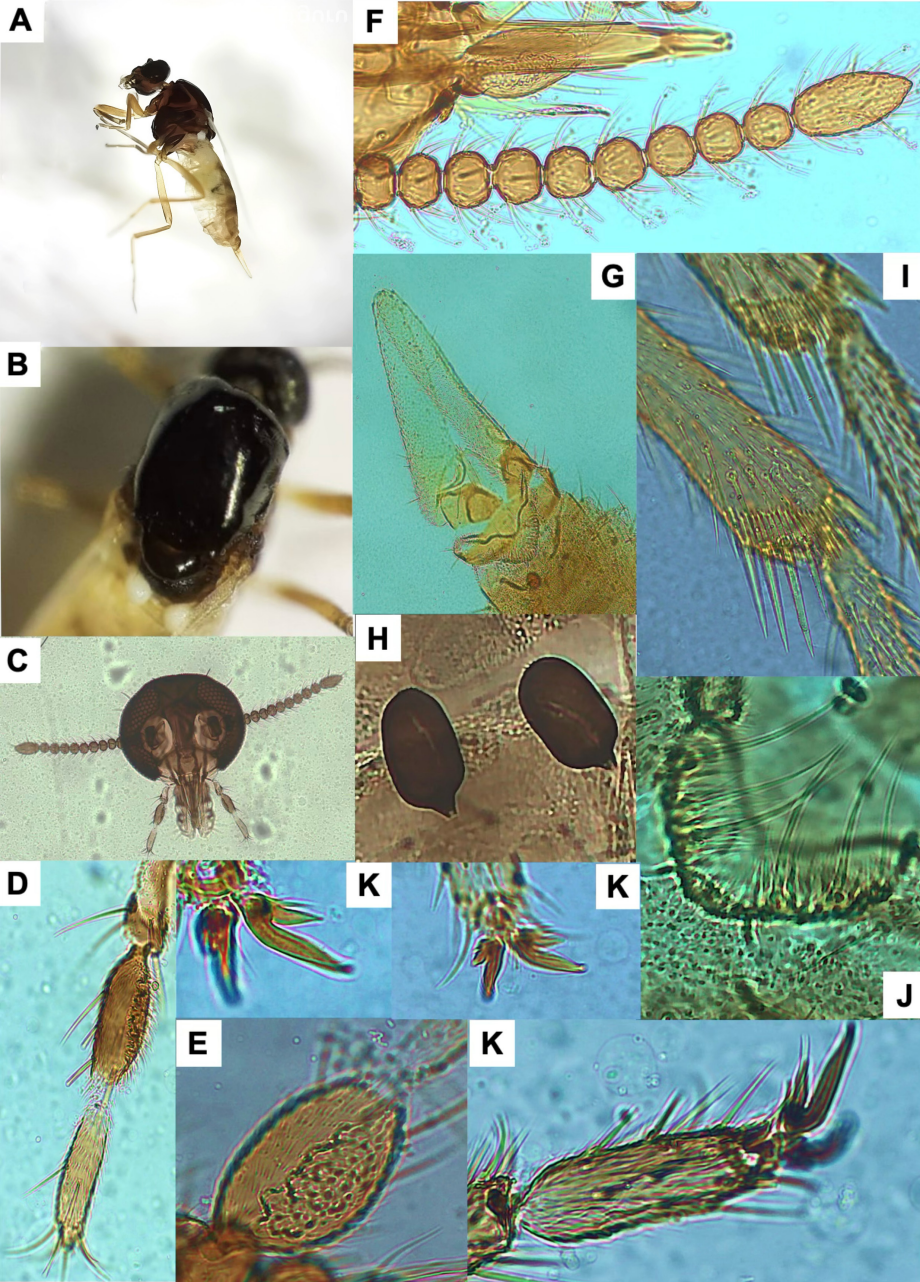
*Leptoconops bidentatus*. **A**. Body (FS); **B**. Thorax (FS); **C**. Head (MS); **D**. Palpus (MS); **E**. Ventral view of third palpal segment (MS); **F**. Antenna (MS); **G**. Apical portion of the abdomen, ventral view; genitalia (MS); **H**. Main spermathecae (MS); **I**. Tibial comb (MS); **J**. Genital atrium (MS); **K**. Claws in different view (MS).

**Figure 11.**
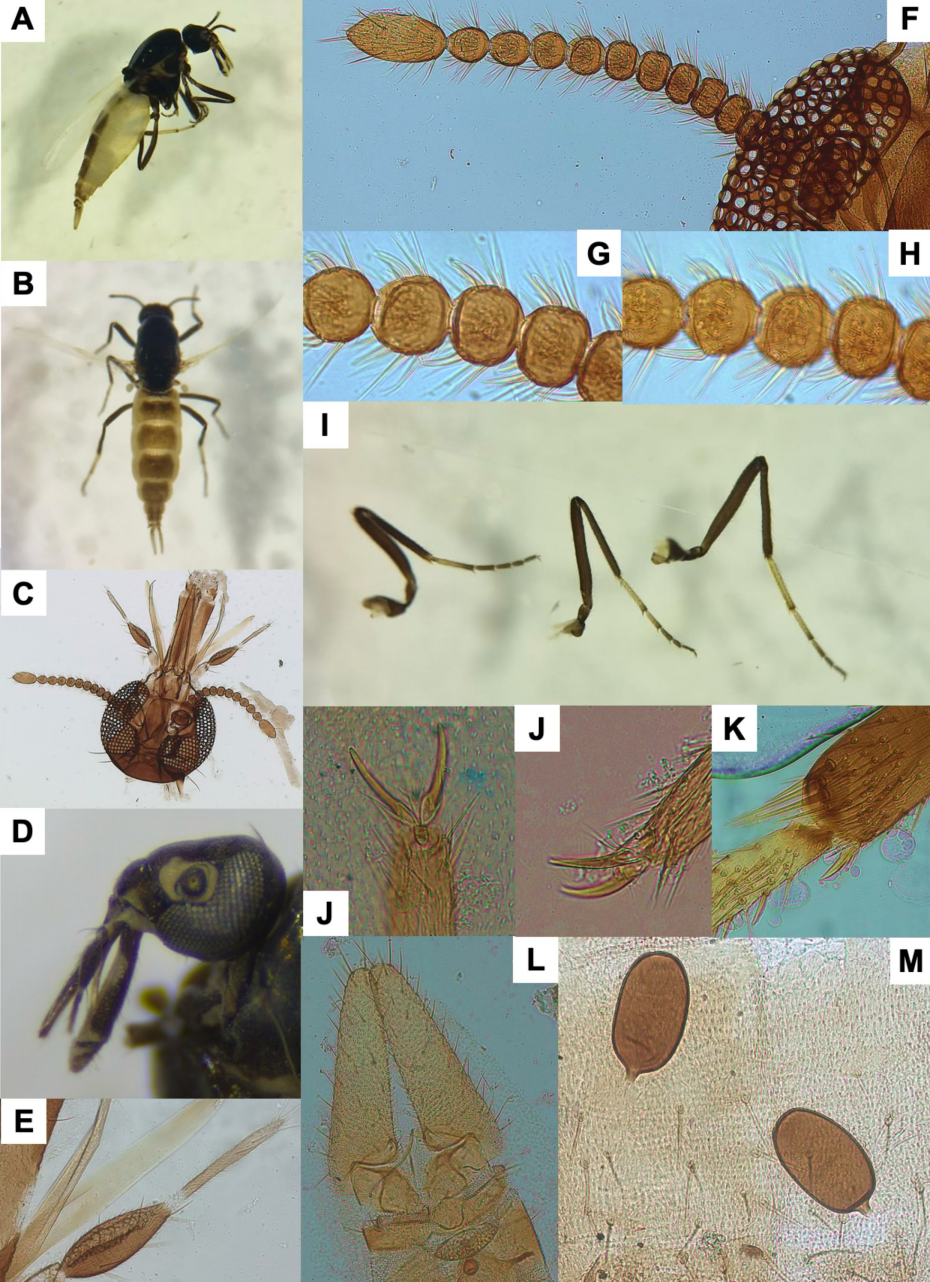
*Leptoconops irritans*. **A, B**. Body (FS); **C**. Head (MS); **D**. Mouthparts (MS); **E**. Palpus (MS); **F**. Antenna (MS); **G, H**. Hyaline setae and sensilla trichodea, respectively (MS); **I**. Foreleg, midleg and hindleg (from right to left) (FS); **J**. Ventral and lateral view of claws (MS); **K**. Tibial comb (MS); **L**. Apical portion of the abdomen, ventral view; genitalia (MS); **M**. Main spermathecae (MS).

**Figure 12.**
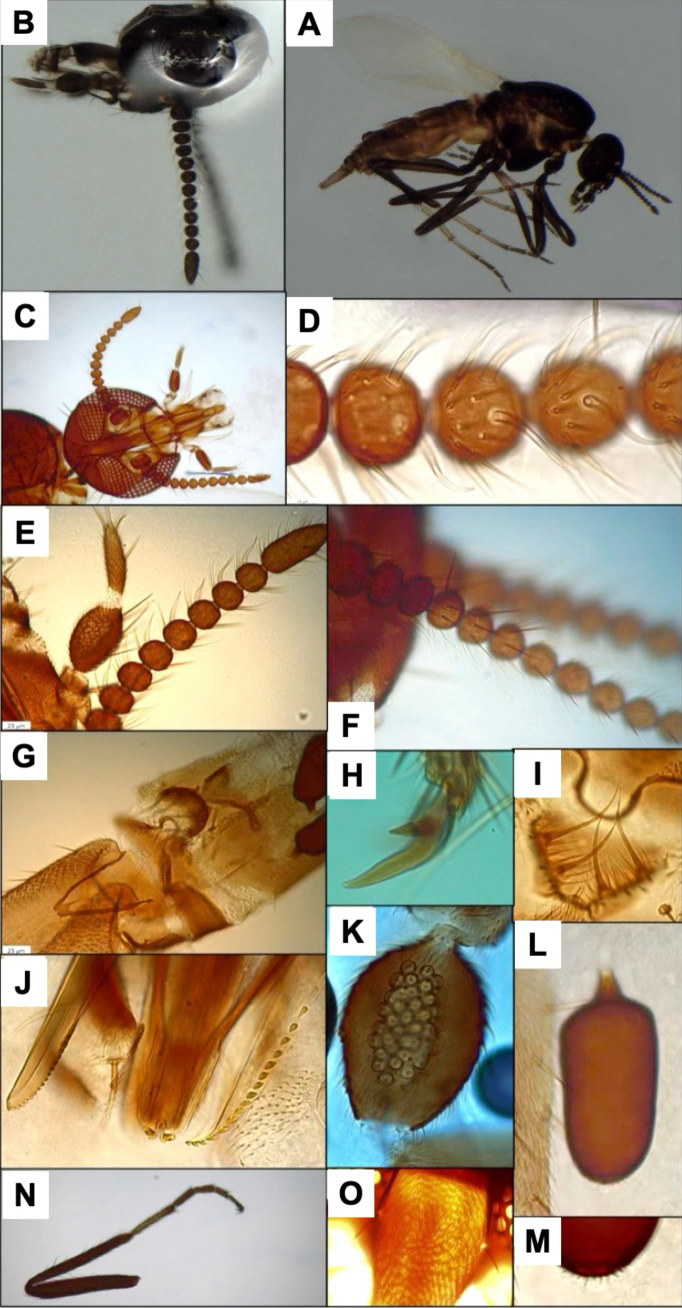
*Leptoconops noei*. **A**. Body (FS); **B**. Antenna (FS); **C**. Head (MS); **D**. Hyaline and hair sensory setae (MS); **E**. Palpus and antennae detail (MS); **F**. External side, dark setae (MS); **G**. Armature of the anal cone (MS); **H**. Claw (MS); **I**. Anal bristles (MS); **J**. Mandible and lacinia teeth (MS); **K**. Third palpal segment (MS); **L**. Spermatheca (MS); **M**. Detail of terminal pubescence (MS); **N**. Foreleg (FS); **O**. Interocular setae (MS). All samples examined were collected in Marina di Alberese Italy.

**Figure 13.**
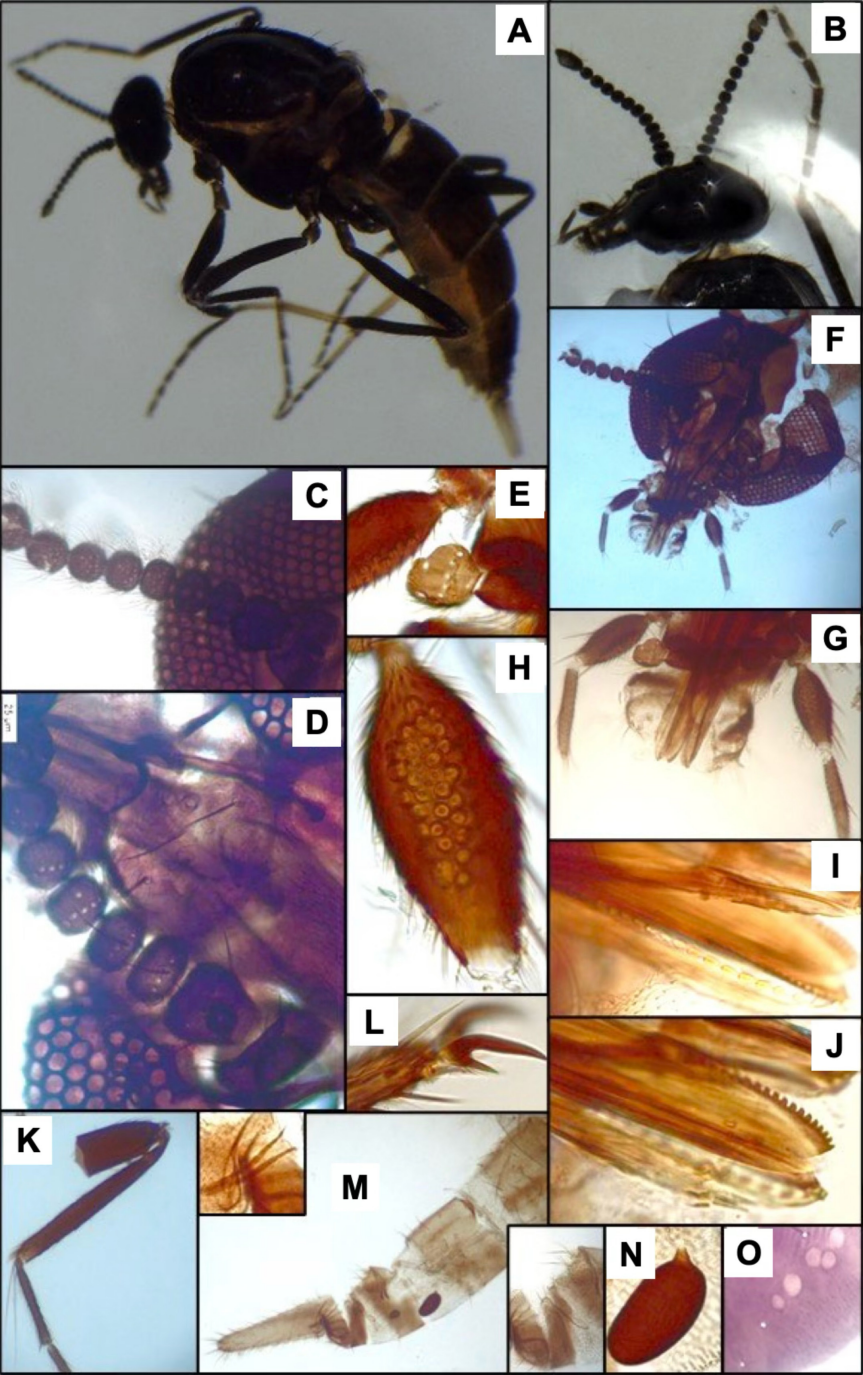
*Leptoconops bezzii*. **A**. Body (FS); **B**. Antennae (FS); **C**. Flagellomeres (internal side) showing hair setae (MS); **D**. Flagellomeres external side (showing sensilla trichodea and chaetica) (MS); **E**. Flagellomere showing hyaline setae (MS); **F**. Head (MS) **G**. Proboscis (MS); **H**. Third palpal segment (MS); **I**. Mandibular teeth (MS); **J**. Lacinia teeth (MS); **K**. Foreleg with dark first tarsomere (FS); **L**. Claw (MS); **M**. Abdomen and lamellae, details of armature of the anal cone and anal bristles (MS); **N**. Spermatheca (MS); **O**. Humeral pits (MS). All samples are from Marina di Alberese Italy.

#### Female

**Table:** 

1	Claws simple or with an inconspicuous tooth; proboscis length (> 225 µm); terminal palpal segment large and slender (> 100 µm); most antennal flagellomeres flattened or subspherical	**2**
–	Claws with a distinctive tooth; proboscis length (< 225 µm); terminal palpal segment (< 100 µm); antennal flagellomeres mixed (flattened, spherical or subspherical	**3**
2	Claws simple without tooth (Fig. 11J); proboscis length > 300 µm. Sensory pit longitudinally distributed along the third palpal segment; > 40 sensilla (Fig. 11E); sensory pit area > 2,000 µm^2^; PR > 2.4; third palpal segment index > 9. Spermathecae longer than wide (Fig. 11M). Flagellomere XII with five sensilla chaetica	***L. irritans* Noé, 1905**
–	Claws with bristle-like teeth (Suppl. material 1: fig. S17D–F); proboscis length < 280 µm. Sensory pits aggregated centrally; < 40 sensilla (Fig. 5D); sensory pit area < 1,000 µm^2^; PR < 2.4; third palpal segment index < 7. Spermathecae nearly as long as wide (Fig. 5I). Flagellomere XII usually with 4 sensilla chaetica	***L. pseudoirritans* sp. nov**.
3	At least with 1 claw with 2 teeth (bidentate) (Fig. 10K); anal cone with 4–8 setae (Fig. 10J); proboscis short (Fig. 10C); third palpal segment small with no more than 30 pits in 3 or 4 irregular rows (Fig. 10E)	***L. bidentatus* Gutsevich, 1960**
–	Claws with 1 tooth; anal cone with 4 setae (rarely 5); proboscis with normally sized; third palpal segment well-developed with > 30 pits in ≥ 4 irregular rows	**4**
4	Spermathecae oval-shaped (< 50 µm) (Fig. 1I, J); sum of total number of lacinia and mandible teeth > 40; hyaline ss arranged diametrically opposed (Fig. 1E)	***L. nigrithorax* sp. nov**.
–	Spermathecae sausage-shaped (> 50 µm); hyaline ss fragile and flexible, arranged sub-laterally and positioned (close to each other); total number of lacinia and mandible teeth < 40	**5**
5	Third palpal segment (ventral view): 1.5 × longer than wide; > 70 sensilla (Fig. 3C). Spermathecae length > 70 µm (Fig. 3I, J)	***L. triangularis* sp. nov**.
–	Third palpal segment (ventral view) variable; sensilla variable; spermathecae length < 70 µm	**6**
6	Third palpal segment twice as long as wide with ~ 30 pits (Fig. 13H). Pair of hyaline ss widely separated (diametrically opposed) on flagellomeres. Abundant hair-like setae distributed in 2 irregular rows on the internal side and 1 on the external side of antenna (Fig. 13C–E). One black seta in the middle of the row on flagellomeres II–IV, and 6 black setae on flagellomere I. Spermathecae length < 65 µm, with no apical pubescence (Fig. 13N)	***L. bezzii* Noé, 1905**
–	Third palpal segment nearly as wide as long; pits variable. Pair of hyaline ss on flagellomeres narrowly separated (emerging from the sublateral of the same face). Black setae on flagellomeres II–XI are arranged differently. Spermathecae with or without apical pubescence	**7**
7	Ventral third palpal segment with numerous irregular rows (8–12) and > 70 pits (Fig. 7D). Spermathecae oval and very large (length > 65 µm) (Fig. 7H); with no apical pubescence on terminal end (Fig. 7H). Armature of the anal cone with a small or inconspicuous protuberance in the middle (Fig. 7I). Black setae (one antenna: > 25) on flagellomeres II–XII	***L. communis* sp. nov**
–	Ventral third palpal segment with a moderate number of rows (6–8) and < 45 pits (Fig. 12K). Spermathecae sausage-shaped and large (length < 65 µm) (Fig. 12L), with apical pubescence (Fig. 12M). Armature of the anal cone with a robust central protuberance (Fig. 12G). Black setae (one antenna: < 25) on flagellomeres II–XII (Fig. 12F)	***L. noei* Clastrier & Coluzzi, 1973**

### Description of new species of *Leptoconops*

Following the International Code of Zoological Nomenclature (ICZN), details of the new species have been submitted to ZooBank with a Life Science Identifier (LSID) zoobank.org.pub: https://zoobank.org/98a2a4fe-5f52-4cea-a976-0431c4ca7d81.

#### Leptoconops nigrithorax

Taxon classification

Animalia

DipteraCeratopogonidae

González & Tanase sp. nov.

D3BFF2BA-EB50-581F-9641-72A91D4E7136

https://zoobank.org/59EDFCFD-4251-4A32-9CB2-D737D4B0FBDA

Fig. 1, Suppl. material 1: figs S1–S6

##### Material examined.

***Holotype***: SPAIN • ♀; Las Pachecas, Cádiz; 36.590805, -6.098175, alt. 61 m; 29 May 2023; M. A. González leg.; BGS trap; EBD I-016375**. *Paratypes***: SPAIN • 1 ♀; labelled as for the holotype, except for EBD I-016376 - 3 ♀; labelled as for the holotype, except for NHMUK014316789-NHMUK014316791 - 2 ♀; Los Palacios, Sevilla; 37.14261, -6.042228, alt. 1 m; 21 Jun 2023; M. A. González leg.; BGS trap; MNCN_Ent 283581.

##### Diagnosis.

*Leptoconops nigrithorax* sp. nov. can be distinguished from other European *Leptoconops* species by the combination of the following characters: (i) shiny black thorax, visible both dorsally and laterally under a stereomicroscope (Fig. 1A, B), (ii) uniformly dark brown legs, without pale coloration on tarsomeres (Fig. 1F–H), and (iii) a higher number of lacinial teeth. In addition, the type and distribution of antennal sensilla also provide useful diagnostic features.

##### Description.

Females large (2.06 ± 0.14 mm). Specimens dark, with a shiny black thorax (Fig. 1A, B, Suppl. material 1: fig. S1A, B), brownish abdomen, and uniformly dark-brown legs (Fig. 1F–H).

***Head***. Subspherical, with dull metallic green tones on clypeus, frons, and vertex (Suppl. material 1: fig. S1C, D). Head length: 343.52 ± 7.59 µm; width: 377.61 ± 7.85 µm (Suppl. material 1: fig. S1D). Head width is 4.2-4.5 × the interocular distance. Two (occasionally 1) lateral interocular setae present, one each side (Suppl. material 1: fig. S1F). Vertex moderately spiculated (400 × magnification) and with 10–12 supraorbital setae arranged in a row (Suppl. material 1: fig. S1E). Eyes bare, moderately separated by a diameter of 7.28 ommatidia (85.89 ± 2.67 µm) (Suppl. material 1: fig. S1F).

***Antenna***. Pedicel with four setae; flagellomere I has five or six setae. Lengths of flagellomeres I–XII are provided in Suppl. material 1: table S2. Total antennal length = 388.75 ± 29.39 µm; AR = 0.7. Proximal flagellomeres II–IV are longitudinally flattened, while V–VI vary from flattened to subspherical; distal flagellomeres VII–XI are spherical (Suppl. material 1: fig. S2A, B). Terminal flagellomere elongate and similar in length to the preceding three flagellomeres combined: densely setose with three or four sensilla chaetica on the anterior mid-section and one or two minute hyaline ss (Suppl. material 1: fig. S2C). Flagellomeres armed with abundant long sensilla trichodea that extend beyond the base of the next segment (sum of both faces): 6–10 on flagellomeres I–IV and 18–22 on flagellomeres V–XI. The sensilla trichodea are arranged in two or three semi-verticils on the external face and in a basal whorl on the internal face (Fig. 1D). Straight and thick hyaline ss (one pair per flagellomere, II–XI) are diametrically opposite (facing each other), tips reach the base of the next flagellomere (Fig. 1E). One minute hyaline ss located on one side of flagellomeres II–XI (sometimes variable and/or difficult to see). No sensilla chaetica on flagellomeres except on I (occasionally 1 on II).

***Mouthparts***. Dark brown (Suppl. material 1: fig. S3A). Clypeus with four frontoclypeal setae (similar length and thickness) arranged trapezoidally: the anterior pair closer but the posterior pair widely separated (Suppl. material 1: fig. S1G). Proboscis (216.98 ± 8.71 µm) shorter than the head. Palps slightly exceed the proboscis tip. Palpus length: 222.57 ± 7.31 µm. PR = 2.06 (1.80–2.32). The first two palpal segments (length: 44.17 ± 7.74 µm) bear three long setae. The third palpal segment (Fig. 1C) is longer than wide (94.95 ± 4.78 µm and 46.41 ± 6.19 µm), slightly incrassate and with 8–10 setae (3 on outer margin, 2 or 3 in sensory area, and 3 or 4 on inner margin). Sensorium with 35–40 sensory pits arranged in 4–6 irregular rows, covering roughly three-quarters of the surface area. The sensillar area measures 1,500.74 ± 93.06 µm^2^. The fourth segment (83.75 ± 1.81 µm) cylindrical, with one or two median setae (usually 1) and 3–5 (usually 4) apical/subapical setae. Lacinia with 22–30 evenly arranged teeth (Suppl. material 1: fig. S3B). Mandible is armed with 19–24 teeth (Suppl. material 1: fig. S3C).

***Thorax***. Mesonotum polished, shiny black, length 660.10 ± 34.10 µm, width 440.24 ± 10.00 µm (Fig. 1B, Suppl. material 1: fig. S4A, B); setose, with three longitudinal straight rows of dark, distinct setae (1 acrostical and 2 dorsocentral, 14–16 setae per row) (Suppl. material 1: fig. S4C, D). Scapular setae are present and a pair of longer prescutellar setae also present. Anterolateral scutal margin with 15–22 scattered setae per side (Suppl. material 1: fig. S4D). Lateral thoracic sclerites polished and shiny, often with reddish mahogany tones, especially in clefts (Suppl. material 1: fig. S4B). Humeral pits absent or inconspicuous. Katepisternum shiny black, with 8–10 scattered setae on each side. Scutellum pruinose with two pairs of long medial setae (subapical and lateral) of similar length (Suppl. material 1: fig. S4E). Post-scutellum strongly arched and bare, with ornamentation and distinct metallic green tones (Suppl. material 1: fig. S4F). Halters white apically, with dark basally (Suppl. material 1: fig. S4F).

***Legs***. Uniformly dark brown, not shiny (Fig. 1F–H). Measurements in Suppl. material 1: table S3. Hindleg (1,896.39 ± 105.81 µm) longer than both foreleg (1,529 ± 50.15 µm) and midleg (1,689.31 ± 77.10 µm). Coxa with six bristles; distal trochanters, basal femur, and tibia possess chordotonal organs (Suppl. material 1: fig. S5D). Trochanter surface with 5–7 long, distinctive bristles plus two short straight bristles and 14–16 chordotonal organs (arranged in two groups, 5–7 + 8–10), femur with 12 bristles arranged in two lines (4 + 8), and tibia with three or four (Suppl. material 1: fig. S5D). Tarsomeres of legs covered with uniformly fine pilosity; femur and tibia with a variable number of long distinct bristles arranged in rows. Typically, at least two lines of bristles arranged in one row, with the other two or three lines mixed. Both anterior and posterior apical regions of the femur each with two or three additional long bristles. Tibial comb with four spines (outer spines shorter than inner ones) (Suppl. material 1: fig. S5A). Apical spines are present on the first (1 or 2, except hind tarsomere, which is variable, having 2–6 strong spines) and second (usually 2) tarsomeres but absent on the third and fourth (variable on the third of the hindlegs). The basitarsus is highly setose in all legs; claws symmetrical, each with a narrow, pointed, moderate-sized basal tooth (Suppl. material 1: fig. S5B, C); empodium with a branched bristle.

***Wing***. Length: 1.08 ± 0.01 mm; width: 0.50 ± 0.03 mm; CR: 0.39 (0.38–0.41). Veins are distinct, radial cells highly sclerotized, with yellow tones (Suppl. material 1: fig. S5E).

***Abdomen***. Tergites (Suppl. material 1: fig. S6A) and sternites (Suppl. material 1: fig. S6B) brown to dark brown, pleural membrane with a leathery texture. Ventral setae are abundant on segments 1–5 (> 30), moderate on segments 6 and 7 (15–22), and scarce on segments 8–10 (< 15). Two small functional spermathecae of equal size (47.09 ± 3.82 µm × 28.76 ± 3.12 µm and 45.79 ± 4.78 µm × 27.65 ± 2.05 µm), oval, and highly sclerotized, with an inconspicuous uncolored area apically and a short, well-formed neck (5.65 ± 2.05 µm) (Fig. 1I, J, Suppl. material 1: fig. S6D). Two small accessory spermathecae present, one bigger than the other (depending on how placed during preparation; the smallest may be difficult to observe) (Fig. 1I, J).

***Genitalia***. On sternite VIII the extremities of the lateral arms of the ventral plate are sclerotized, pointed, and membranous at the base (with stretch marks). These arms are orientated horizontally outwards or slightly curved (Suppl. material 1: fig. S6C). Ventral extremities of segment IV are shaped in a sclerotized structure with three or four bristles. Genital atrium semicircular (slightly wider than long), with four large, thick, curved bristles (arising from a pediculate protuberance) on each side posteriorly and numerous weak bristles evenly arranged at the base (Suppl. material 1: fig. S6E). Behind the atrium lies a pigmented structure with an excavation in the middle section, sometimes featuring a tiny projection. Genital armature of anal cone large, V-shaped ventrally, with a trapezoidal and well-sclerotized upper part (Suppl. material 1: fig. S6C, E). Genital lamella dark, elongate (255.64 ± 7.32 µm × 69.37 ± 9.38 µm, length × width; index: 3.7, 3.10–4.49) bearing 55–70 setae (Fig. 1I, Suppl. material 1: fig. S6C).

**Male**. Unknown.

##### Etymology.

The specific epithet nigrithorax refers to the shiny black thorax that characterizes this species.

##### Distribution.

This species was detected at numerous different sites across the three sampled provinces (Fig. 2).

##### Habitat.

Specimens were collected in large numbers (< 1,000) from Mediterranean shrubs near arable land (diverse crops), pastures, and small water bodies (e.g. creeks) (Fig. 9A–D).

##### Notes.

Ethanol alters the coloration of some structures in fresh specimens. The shiny appearance is less evident in ethanol preserved specimens. Lighting conditions also influence the perception of colors and tones.

#### Leptoconops triangularis

Taxon classification

Animalia

DipteraCeratopogonidae

González & Tanase sp. nov.

E51EB593-0E15-5D56-B318-C721398484A8

https://zoobank.org/3F1772E7-1B60-4B3C-B909-4E0C093BC428

Fig. 3, Suppl. material 1: figs S7–S12

##### Material examined.

***Holotype***: SPAIN • ♀; Pago del Humo, Cádiz; 36.417118, -6.090697; alt. 11 m. 06 Jun. 2024; M. A. González leg.; BGS trap; EBD I-016377**. *Paratypes***: SPAIN • 1 ♀; labelled as for the holotype, except for EBD I-016378 - 1♀; La Canaleja, Cádiz; 36.425053, -5.9079281; alt. 54 m; 6 Jun 2024; M. A. González leg.; BGS trap; MNCN_Ent 283582 - 1♀; Vejer de la Frontera, Cádiz; 36.282130, -5.963067; alt. 59 m; 6 Jun 2024; M. A. González leg.; BGS trap; NHMUK014316788 - 1♀; Écija, Sevilla; 37.448530,-5.1076545; alt. 183 m; 18 Jun 2024; M. A. González leg.; BGS trap; NHMUK014316787 - 1♀; Écija, Sevilla; 37.448530,-5.1076545; alt. 183 m; 18 Jun 2024; M. A. González leg.; BGS trap; MNCN_Ent 283583 - 1♀; Vejer de la Frontera, Cádiz; 36.282130, -5.963067; alt. 59 m; 6 Jun 2024; M. A. González leg.; BGS trap; MNCN_Ent 283584.

##### Diagnosis.

The abdominal terga possess characteristic sharply defined, black triangular spots that are large on segments I–IV but become progressively smaller and more diffuse towards the apex (segments V–VII) (Fig. 3B). This character is unique among the *Leptoconops* species recorded in the Western Palearctic.

##### Description.

Females medium-sized (1.63 ± 0.10 mm). Thorax dorsally pruinose with green metallic reflections; abdomen pale cream to light brown (almost orange), with distinctive black triangular spots on the tergites; legs bicolored (Fig. 3A, B, Suppl. material 1: fig. S7A, B).

***Head***. Subspherical, dull-pruinose dark with metallic-green tones on the clypeus, frons, and vertex (Suppl. material 1: fig. S7C, D). Head width (445.12 ± 43.02 µm) exceeds head length (372.30 ± 34.55 µm) (Suppl. material 1: fig. S7D). Head width 4.3 × (4.1–4.6) the interocular distance. Two lateral interocular setae present (one per side) that are sometimes hard to see (Suppl. material 1: fig. S7E). Vertex moderately spiculated under 400x magnification and have 8–10 setae aligned in a row, four setae within the upper interocular space. Eyes bare, separated by the diameter of 8 ommatidia (102.68 ± 14.35 µm) (Suppl. material 1: fig. S7D, E).

***Antenna***. Pedicel usually with three setae; flagellomere I has six setae (4 + 2). Lengths of flagellomeres I–XII are provided in Suppl. material 1: table S2. Total antennal length = 485.68 ± 17.92 µm; AR = 0.68. Proximal flagellomeres II–IV longitudinally flattened, V subspherical, and VI–XI becoming progressively spherical (Suppl. material 1: fig. S8A). Terminal flagellomere elongate, similar in length to the preceding 2–2.5 segments combined, densely setose, with various minute hyaline ss and four or five sensilla chaetica (1 apical, 1 basal, and 3 beyond the mid-section) (Fig. 3D, Suppl. material 1: fig. S8B). Antenna with 8–14 sensilla trichodea arranged in two or three semi-verticils on both faces of each flagellomere (Suppl. material 1: fig. S8C). Distinctive and conspicuous sensilla chaetica present on one face: three or four long and curved on flagellomeres I–VIII, and one or two entirely straight on flagellomeres IX–XI (Suppl. material 1: fig. S8D). Minute hyaline ss arranged on the distal third in variable numbers (2–5 per flagellomere). A pair of thin hyaline ss present on flagellomeres I–XI, situated sub-laterally on one face, curved beyond mid-length, and proximate (Fig. 3E, Suppl. material 1: fig. S8E).

***Mouthparts***. Dark brown. Clypeus setae with the same configuration as the previous species (Suppl. material 1: fig. S7F). Proboscis 204.27 ± 18.49 µm, shorter than head length. Palps slightly protrude beyond the proboscis tip; total palpal length 226.83 ± 19.83 µm; PR = 1.56 (1.40–1.70). First two palpal segments (length 44.36 ± 11.51 µm) each with three long setae. Third segment (Fig. 3C) large and moderately incrassate, markedly longer than wide (97.25 ± 4.28 µm and 62.43 ± 5.01 µm), with 6–11 setae (2–4 on the outer margin, 2–4 in sensory area, and 2 or 3 on the inner margin). Sensorium with 70–85 sensilla arranged in 6–8 irregular rows, occupying ~3/4 of surface with sensory pits; sensillar area 1,755.57 ± 35.31 µm^2^. Fourth segment (85.21 ± 8.31 µm) cylindrical, with one or two median setae (usually 1), one or two subapical setae (usually 1), and two or three apical setae (Fig. 3C, Suppl. material 1: fig. S9). Lacinia has 21–24 teeth, typically curved apically (Suppl. material 1: fig. S9). Mandible armed with 15–20 teeth (Suppl. material 1: fig. S9).

***Thorax***. Mesonotum pruinose with green or greyish reflections, depending on light, length 553.75 ± 30.08 µm, width 451.66 ± 35.16 µm (Suppl. material 1: fig. S10A). Mesonotum bears brown setae arranged in three longitudinal rows (one acrostical and two dorsocentral), and with scattered setae antero- and post-laterally. Three distinctive supraalar setae present on each side. Humeral callus prominent with angular edges (Suppl. material 1: fig. S10A). Humeral pits consisting of three or four small, rounded black spots aligned anteriorly, with an additional group of three or four black pits centrally in the prescutellar area (Suppl. material 1: fig. S10B, C). Anepisternum without setae; katepisternum pruinose with 8–10 scattered setae on each side. Scutellum pruinose with two (subapical and lateral) or three (1 subapical and 2 lateral, the outer lateral pair shorter) pairs of setae. Post-scutellum strongly arched, pruinose, and bare. Halters yellow apically and dark basally (Suppl. material 1: fig. S10B).

***Legs***. Bicolored, with alternating dark and pale patterns. Measurements in Suppl. material 1: table S3. Foreleg (1,545.09 ± 58.79 µm) and midleg (1,628.80 ± 124.13 µm) shorter than hindleg (2,066 ± 98.10 µm). Femur and tibia uniformly dark brown. Foreleg tarsomeres: first bicolored (three-quarters dark, one quarter pale), others uniformly dark (Fig. 3F). Midleg and hindleg tarsomeres: first and second pale except for dark apical tips; others uniformly dark (Fig. 3G, H). Trochanter, femur, and tibia bear 12–14 (2 groups), 10–12 (basal region, usually 8 + 4), and three or four (basal region), respectively, chordotonal organs. Trochanter with eight bristles (6 distinctive and long, 2 weak, closely positioned, straight). Leg surface with fine pilosity and 3–5 rows of setae arranged in a row or irregularly. Apical regions of femur and tibia have additional long bristles. Tibial comb with four spines, the central two equal in size (largest), the outer two smaller and unequal (Fig. 3K, Suppl. material 1: fig. S11A). Hind basitarsus highly setulose. Apical spines present on the first (1 spine) and second (1 spine) tarsomeres of foreleg, on first, second, third, and fourth (1 or 2 spines each) tarsomeres of midleg, and on first (2–4 spines), second (2 spines), third (1 or 2 spines), and fourth (variable) tarsomeres of hindlegs. Claws similar on all legs, slightly undulate in profile, symmetrical, each with a thick, pointed basal tooth and a bristle arising from its base (Suppl. material 1: fig. S11B, C). Empodium with a branched bristle.

***Wing***. Length 1.14 ± 0.09 mm; width of 0.46 ± 0.02 mm, CR: 0.43 (0.39–0.53). Veins distinct; radial cells highly sclerotized and yellowish (Suppl. material 1: fig. S11D, E).

***Abdomen***. Tergites and sternites pale fawn to pale brown. Tergites 1–4 with well-defined triangular black spots (Fig. 3B). Ventral setae more abundant on segments 1–5 (> 30), moderately numerous on segments 6 and 7 (15–25), and less abundant on segments 8–10 (< 16). The two functional elongate spermathecae are nearly equal in size (74.54 ± 6.3 × 22.29 ± 2.03 µm and 70.71 ± 3.65 × 45.76 ± 1.53 µm), highly sclerotized, and have a discolored apical region (minute yellow spots), and a distinct tronco-conical sclerotized neck (9.89 ± 1.26 µm) (Fig. 3I, J, Suppl. material 1: fig. S12A, D). A small, rudimentary third spermatheca is present (Suppl. material 1: fig. S12A).

***Genitalia***. Sternite VIII and the extremities of the lateral arms of the ventral plates show basal striations and well-developed extremities with variable orientation (Suppl. material 1: fig. S12B, C). Ventral extremities of segment IV form a sclerotized structure with two or three fine bristles. The genital atrium is semicircular (wider than long), with four long, strongly curved bristles, arising from enlarged posterior bases, of similar size. Base with a moderate number of weaker bristles (Suppl. material 1: fig. S12B, C). Behind the atrium lies a pigmented structure with a concave medial region and sclerotized margins. Genital armature of the anal cone large, V-shaped in ventral view, and strongly sclerotized (Suppl. material 1: fig. S12A–C). Lamella dark greyish, not especially long (195.65 ± 15.45 µm × 71.50 ± 6.85 µm, length × width; index 2.7, 2.2–3.5), and bear 50–60 scattered setae, longer apically (Fig. 3I, J, Suppl. material 1: fig. S12A).

**Male**. Unknown.

##### Etymology.

The specific epithet triangularis refers to the distinctive triangular black spots on the abdominal tergites that characterize this species.

##### Distribution.

This species is known from just a few locations in the three sampled provinces (Fig. 4).

##### Habitat.

Specimens were collected in low numbers (< 20) from Mediterranean shrub vegetation bordering arable land (Fig. 9A, B, D).

#### Leptoconops pseudoirritans

Taxon classification

Animalia

DipteraCeratopogonidae

González & Tanase sp. nov.

F334D35D-3AAC-5BFA-86F2-07CFD0667B57

https://zoobank.org/F286CF9A-2967-4FF5-ABCD-EC77B71C938F

Fig. 5, Suppl. material 1: figs S13–S18

##### Material examined.

***Holotype***: SPAIN • ♀; Alcalá de Guadaíra, Sevilla; 37.288340, -5.778454; alt. 45 m. 6 Jun. 2024; M. A. González leg.; BGS trap; EBD I-016379**. *Paratypes***: SPAIN • 1 ♀; labelled as for the holotype, except for EBD I-016380; 2 ♀; Carmona, Sevilla; 37.445281, -5.630657; alt. 87 m; 09 Aug 2023; M. A. González leg.; BGS trap; NHMUK014316795; NHMUK014316796 - 1 ♀; Arahal, Sevilla; 37.26508016923277, -5.636044311686363; alt. 65 m; 06 Jun 2024; M. A. González leg.; BGS trap; NHMUK014316794 - 1 ♀ labelled as previous paratype, except for NHMUK014316793 - 1 ♀; El Palomar, Sevilla; 37.370435, -5.500349; alt 113 m; 10 May 2023; M. A. González leg.; BGS trap; MNCN_Ent 283585 - 1 ♀; Arahal, Sevilla; 37.26508016923277, -5.636044311686363; alt. 65 m; 06 Jun 2024; M. A. González leg.; BGS trap; MNCN_Ent 283586.

##### Diagnosis.

Claws with bristle-like teeth; proboscis length shorter (< 280 µm). Sensory pits with < 40 sensilla (Fig. 5D). Spermathecae nearly as long as wide (Fig. 5I). Flagellomere XII usually with four sensilla chaetica.

##### Description.

Females small (1.52 ± 0.06 mm). Thorax dorsally pruinose with metallic green reflections; abdomen pale cream to brown; legs bicolored (Fig. 5A, Suppl. material 1: fig. S13A).

***Head***. Subspherical, dull pruinose to dark brown, with metallic-green tones (Suppl. material 1: fig. S13B–D). Head length 347.93 ± 11.51 µm, slightly exceeding width 337.44 ± 14.61 µm. Head width is 3.7 **×** (3.3–4.0) the interocular distance; two lateral setae are present, one on each side. Vertex with around ten supraorbital setae arranged in a single row; surface slightly spiculate at 400 × magnification (Suppl. material 1: fig. S13E). Eyes bare, separated by the diameter of 8.2 ommatidia (89.41 ± 6.54 µm) (Suppl. material 1: fig. S13D, E).

***Antenna***. Pedicel with 2–5 long bristles; flagellomere I with five (4 + 1) bristles. Lengths of flagellomeres I–XII are provided in Suppl. material 1: table S2. Total antennal length = 340.20 ± 17.46 µm; AR = 0.64. Flagellomeres I–XI slightly flattened, subspherical (Suppl. material 1: fig. S14A). Terminal flagellomere (Suppl. material 1: fig. S14B) thick and club-shaped, markedly wider than the preceding segments, densely setose, similar in length to the previous two segments combined and with four sensilla chaetica on the distal third (3 beyond the central part, one subapical). Each of flagellomeres I–XI has a pair of short (similar in length to flagellomere) hyaline ss arranged in pairs (diametrically opposed) (Fig. 5B, Suppl. material 1: fig. S14C). On flagellomeres II–XI, 10–14 sensilla trichodea are arranged in semi-verticils on one face (Fig. 5B). Medium-length sensilla chaetica are present on one face of proximal flagellomeres II–VIII (2–4) and distal flagellomeres IX–XI (0–2) (Fig. 5C). Flagellomeres have a variable number of minute hyaline ss scattered across their surface.

***Mouthparts***. Dark brown. Frontoclypeal setae as described in previous species. Proboscis length (265.04 ± 22.99 µm) shorter than head length (Suppl. material 1: fig. S13B, C). Palps reach the tip of the proboscis. Palpus length 314.52 ± 16.81 µm; PR = 2.28 (2.14–2.39). The first two palpal segments measure 68.29 ± 7.41 µm and each bear three long setae. The third segment (Fig. 5D, Suppl. material 1: fig. S15A–C) is brown, slightly incrassate, with 3–7 setae (1–3 on the outer margin, 1–3 on the sensory area, and 1 or 2 on the inner margin), and is twice as long as wide (97.24 ± 4.76 µm and 42.71 ± 3.88 µm). Sensorial area with 20–30 pits arranged in four or five irregular rows, occupying ½ of the segment surface (Fig. 5D, Suppl. material 1: fig. S15C) (area 987.79 ± 156.29 µm^2^). The fourth palpal segment very elongated and narrow (length: 106.27 ± 10.20 µm and width: 17.14 ± 1.17 µm), with two or three median setae (usually 2), two or three subapical setae (usually 2), and 2–4 apical setae. Lacinia with 13–15 teeth (Suppl. material 1: fig. S15D): Mandible armed with 12–18 teeth (Suppl. material 1: fig. S15E).

***Thorax***. Mesonotum pruinose with green or greyish reflections that vary with light; length 530.20 ± 20.68 µm and width 340.10 ± 10.10 µm (Suppl. material 1: fig. S16A). It has indistinct brown setae arranged in three longitudinal rows (one acrostical and two dorsocentral), plus scattered antero-posterolaterally setae (~30). Present on each side of the mesonotum are two or three conspicuous supraalar setae. The humeral callus is slightly prominent and has angular margins. The humeral pits (3 or 4 circular black spots) are aligned anteriorly, with 2–4 small black pits in the prescutellar area (Suppl. material 1: fig. S16A–C). The anepisternum and katepisternum are similar in color to the mesonotum and have abundant inconspicuous scattered setae (~20). Anepisternum clefts have reddish or yellowish tones. The pruinose scutellum has two subapical and two lateral setae. Post-scutellum pruinose and bare. Halters whitish apically and dark basally (Suppl. material 1: fig. S16D).

***Legs***. Bicolored, not pruinose, with alternating dark and pale patterns. Measurements in Suppl. material 1: table S3. Foreleg (1,368.39 ± 81.67 µm) and midleg (1,383 ± 68.37 µm) shorter than hindleg (1,691.755 ± 136.31 µm). Femur and tibia uniformly dark brown. Foreleg tarsomeres: first variable, usually with a darker basal portion (three-quarters dark, one-quarter pale); other tarsomeres uniformly dark (Fig. 5E). Mid and hindleg tarsomeres: first and second pale except for dark apical tips; other segments uniformly dark (Fig. 5F, G). Coxae and trochanters with 6–9 bristles each (Suppl. material 1: fig. S17B). Basal regions of trochanter, femur, and tibia have 1–12, 4 + 8, and 2–4 chordotonal organs, respectively. Surface of legs has fine pilosity; femur and tibia with three or four longitudinal rows of long setae (sometimes incomplete or partially merged) (Suppl. material 1: fig. S17C). Apical region of femur with supplementary setae, as in the two previously described species. Tibial comb with four spines, the outer ones shorter than the inner ones (Suppl. material 1: fig. S17A). Apical spines present on the first (1 or 2 spines) and second (1 spine) foreleg tarsomeres; on the first (2 spines), second (2 spines), and third (variable, usually 2 spines) midleg tarsomeres; and on the first, second, and third (2 spines each) hindleg tarsomeres. Basitarsus of all legs highly setulose, particularly the hind one. Claws symmetrical, each with a small basal tooth (sometimes inconspicuous) plus one associated bristle (Suppl. material 1: fig. S17D–F).

***Wing***. Length 1.02 ± 0.07 mm; width of 0.39 ± 0.02 mm, and CR: 0.46 (0.41–0.5). Veins distinct, radial cells highly sclerotized, with marked yellow tones (Suppl. material 1: fig. S17G).

***Abdomen***. Tergites and sternites pale fawn to pale brown. Tergites sometimes with faint, blurred dark spots on segments 1–2 (Suppl. material 1: fig. S18A). Ventral abdominal setae abundant on segments 1–5 (> 60), moderate on segments 6 and 7 (25–35), and scarce on segments 8–10 (< 20). Two functional ovoid spermathecae of equal size (48.76 ± 3.11 µm × 41.33 ± 3.72 µm and 50.07 ± 4.49 µm × 41.21 ± 3.07 µm), moderately sclerotized, each with an indistinct discolored apical area, and a small, sclerotized neck (6.18 ± 1.22 µm) (Fig. 5I). A small rudimentary third spermatheca present.

***Genitalia***. On sternite VIII the apices of the lateral arms of the ventral plate have differing orientations and degrees of development but are usually weakly developed (Suppl. material 1: fig. S18B). Ventral surface of extremities of segment IV with two fine bristles. Genital atrium large and semicircular (as tall as wide) with four curved bristles of similar size arising posteriorly, and a variable number of smaller bristles covering the margin (Suppl. material 1: fig. S18C, D). Behind the atrium lies a pigmented structure with a central excavation (Suppl. material 1: fig. S18D). Genital armature of the anal cone trapezoidal in ventral view, with the upper margin weakly sclerotized. Lamella long (241.58 ± 29.8 µm × 70.12 ± 6.15 µm, length × width; index 3.34, 3.07–3.91), slightly darker than abdominal coloration, with 55–65 scattered setae, somewhat longer in the apical region (Fig. 5H, Suppl. material 1: fig. S18B).

**Male**. Unknown.

##### Etymology.

The specific epithet pseudoirritans refers to its close morphological similarity to the nominal species *L. irritans*.

##### Distribution.

This species is known from several locations in the three sampled provinces (Fig. 6).

##### Habitat.

Specimens were collected from Mediterranean scrub adjacent to arable land (diverse crops), although in relatively low numbers (< 100) (Fig. 9A–D).

#### Leptoconops communis

Taxon classification

Animalia

DipteraCeratopogonidae

González & Tanase sp. nov.

8F02C7C8-03CC-5B25-B607-F1847FA459A8

https://zoobank.org/31DB65CA-23F8-43B9-807C-485CF3E2DA48

Fig. 7, Suppl. material 1: figs S19–S25

##### Material examined.

***Holotype***. SPAIN • ♀; Almadén de la Plata, Sevilla; 37.800273, -6.11586; alt. 398 m. 16 May 2024; M. A. González leg.; BGS trap; EBD I-016381**. *Paratypes***: SPAIN • 1 ♀; Zalamea la Real, Huelva; 37.688618, -6.751417; alt. 289 m; 25 Apr. 2024; M. A. González leg.; BGS trap; EBD I-016382 - 3 ♀; Guillena, Sevilla; 37.564304, -6.072888; alt. 85 m. 05 May 2023; M. A. González leg.; BGS trap; EBD I-016383; EBD I-016384 - 1 ♀; labelled as for the holotype except for NHMUK014316792 - 1 ♀; Zalamea la Real, Huelva; 37.688618, -6.751417; alt. 289 m; 25 Apr. 2024; M. A. González leg.; BGS trap; MNCN_Ent 283587.

##### Diagnosis.

Under close magnification (20–40×), the third palp segment in *L. communis* sp. nov. is broadly oval and has abundant sensilla (> 70), with the sensory pit occupying three-quarters of the segment. Spermathecae oval and very large (length > 65 µm) with no apical pubescence on terminal end (Fig. 7H). Armature of the anal cone with a small or inconspicuous protuberance in the middle (Fig. 7I).

##### Description.

Females medium-sized (1.69 ± 0.08 mm). Thorax is pruinose and slightly shiny, with dark-green metallic tones; legs bicolored (foreleg uniformly dark brown, mid and hindlegs with mixed coloration) (Fig. 7A, Suppl. material 1: fig. S19A).

***Head***. Subspherical, polished dark with metallic-green reflections on the clypeus, frons, and vertex (Suppl. material 1: fig. S19C). Head length 368.81 ± 20.38 µm; width 416.42 ± 26.97 µm (Suppl. material 1: fig. S19B). Head width 3.7 × (3.5–3.8) the interocular space (Suppl. material 1: fig. S19B). A single interocular seta on each side was observed in one specimen. Vertex slightly spiculated at 400 × magnification, with twelve supraorbital setae (Suppl. material 1: fig. S19D) arranged in a single a row and three setae situated in the upper interocular space. Eyes bare, separated by the diameter of 11 ommatidia (111.40 ± 5.29 µm).

***Antenna***. Pedicel has three setae; flagellomere I bears four setae arranged in a row (Suppl. material 1: fig. S20C). Lengths of flagellomeres I–XII are provided in Suppl. material 1: table S2. Total antennal length 450.53 ± 28.67 µm; AR = 0.66. Proximal flagellomeres II–IV longitudinally flattened, V–VI subspherical, VII slightly spherical, and distal VIII–XI entirely spherical (Suppl. material 1: fig. S20A, B). Terminal flagellomere elongate, of similar length to the combined length of the preceding 2–2.5 segments, densely setose, with numerous minute hyaline ss and 5–7 sensilla chaetica (1 apical, 3 or 4 subapical and 1 or 2 basal) (Suppl. material 1: fig. S20D). Each flagellomere has 12–16 sensilla trichodea arranged in two or three semi-verticils on both faces (Fig. 7B, Suppl. material 1: fig. S20E). Distinctive, completely straight, conspicuous sensilla chaetica are present on one face: four on flagellomere I, three or four on flagellomeres II–VII and two or three on flagellomeres VIII–XI (Fig. 7B, Suppl. material 1: fig. S20E). Numerous minute hyaline ss are scattered over each flagellomere but are difficult to observe. One pair of hyaline ss on I–XI, thickened basally, thin beyond the middle, abruptly curved near the base (bull-horn shaped), and positioned subapically on one face, with very close insertions (Fig. 7C). In proximal flagellomeres (II–V), the hyaline ss tips usually exceed by one-third to one-half the length of the following flagellomeres; in distal flagellomeres (VI–XI), the tips exceed by ¼ the length of the subsequent flagellomere.

***Mouthparts***. Blackish. Clypeus as described for the preceding species (Suppl. material 1: fig. S19D). Proboscis 224.47 ± 23.83 µm, shorter than head. Palpus not exceeding the proboscis tip; total palpus length 216.12 ± 8.44 µm; PR = 1.29 (1.17–1.29) (Suppl. material 1: fig. S21A). The first two palpal segments 36.56 ± 7.11 µm each bear three distinctive conspicuously long setae (usually exceeding 1/3 the length of the third palp segment). Third palp segment large (Fig. 7D), markedly incrassate, with 11–13 setae (3 or 4 on outer margin, four on sensory area usually arranged in 1 or 2 rows, 3 between the margins on each side, and 3 on inner margin) (Suppl. material 1: fig. S21B, C). Third palpal segment moderately longer than wide (91.11 ± 2.5 µm and 71.08 ± 8.98 µm). Sensorium with 70–80 sensilla arranged in 8–11 irregular rows, occupying ¾ (Suppl. material 1: fig. S21C). Sensillar area: 1,918 ± 76.49 µm^2^. Fourth segment cylindrical (length: 88.44 ± 3.28 µm and width: 22.57 ± 2.89 µm), with one or two median (usually 1), two subapical, and four apical setae (Suppl. material 1: fig. S21D). Lacinia with 21–22 apically curved teeth (Suppl. material 1: fig. S21E). Mandible armed with 21–23 teeth (Suppl. material 1: fig. S21D).

***Thorax***. Mesonotum pruinose, slightly shiny with dark-green metallic tones (depending on light), length 684 ± 43.49 µm and width 426 ± 33.04 µm (Suppl. material 1: fig. S22A–D). Three longitudinal rows of setae present (1 acrostical and 2 dorsocentral), with additional setae scattered antero- and postero-laterally; posterior dorsocentral setae longer (Suppl. material 1: fig. S22C). Three distinctive supraalar setae on each side. Humeral callus prominent, angular, with four bristles (Suppl. material 1: fig. S22C). Four small rounded black humeral pits aligned anteriorly (Suppl. material 1: fig. S22B); four additional distinctive black pits in a paired quadrate pattern in the prescutellar area (Suppl. material 1: fig. S22C). Anepisternum shiny, metallic green, without setae (Suppl. material 1: fig. S22B), with amber tones in the anepisternal cleft (Suppl. material 1: fig. S22E). Katepisternum blackish with metallic-green tones, shiny, with 10–12 scattered setae on each side (Suppl. material 1: fig. S22B). Scutellum pruinose with two setae on each side (1 apical and 1 basal), the basal one the shorter of the two (Suppl. material 1: fig. S22D). Post-scutellum slightly arched, pruinose, and bare. Halters entirely yellowish white (Suppl. material 1: fig. S22A).

***Legs***. Bicolored. Measurements in Suppl. material 1: table S3. Foreleg (1,592 ± 99.43 µm) and midleg (1,773 ± 42.92 µm) shorter than hindleg (1,866.69 ± 50.45 µm). Femur and tibia pruinose, uniformly dark brown. Foreleg: first tarsomere evenly dark brown with light/dark coloration proximally, the remaining segments entirely dark brown (Fig. 7E). Midleg: first tarsomere pale yellowish-brown becoming darker distally; remaining segments entirely dark brown (Fig. 7F). Hindleg: first tarsomere entirely pale yellowish-brown with slightly darker apex; second tarsomere mixed (proximally yellowish brown, dark brown beyond the middle), remainder dark brown (Fig. 7G). Coxa with five or six bristles arranged in a row on each side (Suppl. material 1: fig. S23D). Trochanter with 6–9 scattered bristles (Suppl. material 1: fig. 23E). Proximal trochanter with twelve or thirteen chordotonal organs in two groups (5–7 + 6–7) (Suppl. material 1: fig. S23B); femur with twelve chordotonal organs in two rows (8 + 4) (Suppl. material 1: fig. S23C). Trochanter, femur, and tibia pilose; femur with three rows of bristles; tibia with three irregular proximal rows and four irregular distal rows of setae arranged longitudinally. Tibial comb with four spines (Fig. 7J, Suppl. material 1: fig. S23A), the two median ones longer. Apical spines present on first (2 spines), second (1 spine), and third (1 spine) foreleg tarsomeres; on the first (2 spines), second (1 or 2 spines), and third (1 spine) midleg tarsomeres; and on first (2 or 3 spines), second, and third (1 or 2 spines) hindleg tarsomeres. Basitarsus densely setulose; the remaining tarsomere moderately setulose. Claws symmetrical, straight to slightly curved apically, each with a basal tooth longer than wide, and a small accessory bristle (Suppl. material 1: fig. S24).

***Wing***. Length: 1.16 ± 0.03 mm; width: 0.43 ± 0.02 mm, and CR: 0.39 (0.38–0.41). Veins distinct, radial cells strongly sclerotized, with marked yellowish-brown tones (Suppl. material 1: fig. S25A, B).

***Abdomen***. Tergites and sternites polished, dorsally dark brown (Suppl. material 1: fig. S25C), ventrally slightly paler, with metallic-green reflections (depending on light) (Suppl. material 1: fig. S25D). Setae scattered dorsally and ventrally, abundant on segments 1–5 (> 30), moderately abundant on segments 6 and 7 (16–30) but fewer on segments 8–10 (< 10). Sternites with larger setae than tergites. Tergites with two longitudinal rows of pits (one pair per tergite) (Suppl. material 1: fig. S25C). Sternites 6–8 with three central pits arranged in a triangular configuration (Suppl. material 1: fig. S25D). Two functional oval and elongated spermathecae (65.84 ± 3.93 × 45.31 ± 4.64 µm and 60.73 ± 4.62 × 45.95 ± 5.19 µm), highly sclerotized, lacking apical pubescence on terminal end, with a long, conspicuous, tronco-conical sclerotized neck (10.97 ± 1.36 µm) (Fig. 7H, Suppl. material 1: fig. S25I). A third rudimentary spermatheca present.

***Genitalia***. Sternite VIII with the lateral arms of the ventral plate strongly sclerotized, projecting posteriorly (Suppl. material 1: fig. S25H). Genital atrium large and semicircular, with four curved bristles of similar size orientated posteriorly and a variable number of smaller marginal bristles (Suppl. material 1: fig. S25G). Behind the atrium, sternite IX has a pigmented, highly setulose structure with medial excavation, with three lateral bristles. Genital armature of the anal cone V-shape in ventral view, heavily sclerotized, with a small median protuberance (Suppl. material 1: fig. S25H). Genital lamella elongate (Fig. 7I, Suppl. material 1: fig. S25E), light grey (Suppl. material 1: fig. S25F), (217.84 ± 10.87 µm × 66.31 ± 8.6 µm, length × width; index 3.31, 2.91–3.67), each with 40–50 long, thick, scattered setae, including one long apical seta on each lamella (Suppl. material 1: fig. S25E).

**Male**. Unknown.

##### Etymology.

The species epithet communis refers to its high abundance in the studied region.

##### Distribution.

This species is known from numerous locations in the three sampled provinces (Fig. 8).

##### Habitat.

Specimens were collected in large number (>1,000) from Mediterranean shrubs near arable fields (various crop types) and pastures (Fig. 9A–E).

### Remarks on the systematics of *Leptoconops* species

The new species documented here exhibit morphological traits that clearly separate them from previously described *Leptoconops* species in Europe. By comparing the new species with the descriptions of Western Palearctic members of the subgenus *L.* (*Leptoconops*), the following diagnostic differences were noticed:

*Leptoconops nigrithorax* sp. nov. shares certain morphological characters with species recorded from North Africa, Middle East, Russia, and Western Asia. However, *L. nigrithorax* sp. nov. can be distinguished from *Leptoconops camelorum* Kieffer, 1921, *Leptoconops lucidus* Gutsevich, 1964, *Leptoconops turkmenicus* Molotova, 1967, *Leptoconops caucasicus* Gutsevich, 1953, *Leptoconops hyalinipennis* Kieffer, 1918, *Leptoconops flaviventis* Kieffer, 1918, and *Leptoconops golanensis* Clastrier, 1981 by its uniformly dark-brown tarsomeres, which lack the pale coloration present in those species. It also differs in in the configuration of the palpus and in the number of sensilla (< 30 in the aforementioned taxa). In addition, in *L. lucidus* and *L. turkmenicus* the frons bears two setae, whereas is bare in *L. nigrithorax* sp. nov. Based on its shiny black thorax and entirely black legs, *L. nigrithorax* sp. nov. is morphologically closest to *Leptoconops nigripes* Dzhafarov, 1961, *Leptoconops mesopotamiensis* Patton, 1920, and *Leptoconops montigenus* Clastrier, 1981. Nevertheless, in *L. nigripes* the frons bears two hairs, there are ~ 33 sensilla arranged in three or four irregular rows, and a slightly longer fourth palpal segment, while in *L. nigrithorax* sp. nov. the frons is bare and the configuration of the palpus is different. In *L. mesopotamiensis*, the abdomen is light brown and flagellomeres IV–X are rounded, while in *L. nigrithorax* sp. nov. the abdomen is brown to black and flagellomeres II–XI are flattened to subspherical. Moreover, *L. montigenus* has one seta on each side of the frons and a small sensory area on the third palpal segment (less than half of the inner face), while in *L. nigrithorax* sp. nov. the frons is bare and the palpal sensory area occupies ~ 3/4 of the inner surface.

*Leptoconops triangularis* sp. nov. is morphologically closest to *Leptoconops minutus* Gutsevich, 1973 and *Leptoconops aviarum* Gutsevich, 1973, both recorded from different regions of Russia. However, it can be distinguished from these species by several key characters. The third palpal segment is markedly longer than the fourth, whereas in *L. minutus* the third and fourth segments are nearly equal in length. In addition, *L. triangularis* sp. nov. has distinct triangular black spots on tergites 1–4, a feature absent in both *L. minutus* and *L. aviarum*. Additionally, *L. triangularis* sp. nov. is similar to *L. irritans* and *L. pseudoirritans* sp. nov. in having a distinctly pale cream to yellow abdomen but is noticeably more robust and corpulent than both these species. Unlike *L. irritans* and *L. pseudoirritans* sp. nov., which possess conspicuously elongate proboscis and palpus, in L. triangularis sp. nov. these structures have more standard proportions. Additional diagnostic traits include a mesonotum that is nearly as wide as long (versus distinctly longer than wide in *L. irritans* and *L. pseudoirritans* sp. nov.) and a characteristic pale and dark pattern on the hindlegs. Under higher magnification, the claws bear a pointed basal tooth, differing from the simple claws of both aforementioned related species.

*Leptoconops pseudoirritans* sp. nov is morphologically similar to *L. minutus*, recorded in other regions of the Palearctic, but it differs by the presence of a third spermatheca and well-sclerotized necks on the main spermathecae. As well, *L. pseudoirritans* sp. nov. is morphologically similar to *L. irritans*, above all in its pale-brown abdomen, palpus, and proboscis length. Separating these species under a binocular microscope is challenging. Typically, *L. irritans* is larger (> 1.9 mm) than *L. pseudoirritans* sp. nov. (~ 1.6–1.7 mm). In addition, the proboscis is longer in the latter species (see identification key). On side-mounted specimens, the two species can be reliably separated by diagnostic features and morphometric measurements of the palpus.

*Leptoconops communis* sp. nov. is morphologically similar to *L. caucasicus*, a species recorded in the Caucasus region and other parts of the Eastern Europe. However, *L. communis* sp. nov. can be distinguished by the shape of the antennal flagellomeres, the presence of more sensilla, entirely dark-brown forelegs, and differences in the configuration of its tarsal spines. This species also bears a superficial resemblance to *L. hyalinipennis* and *L. flaviventis*. However, both species have a shiny black mesonotum, whereas in *L. communis* sp. nov. the mesonotum is dark brown with no shiny patterning. Additionally, *L. flaviventis* has a yellow abdomen contrasting with the dark brownish abdomen of *L. communis* sp. nov. Moreover, *L. communis* sp. nov. is morphologically similar to *L. bezzii* and *L. noei*. According to the descriptions by Noé (1905) and Clastrier and Coluzzi (1973), in *L. bezzii* the third palpal segment is longer than wide and has fewer sensilla (~ 30). The spermathecae of *L. communis* sp. nov. possess a distinct and well-developed neck (> 10 µm), whereas in *L. bezzii* it has a smaller and less pronounced neck (< 10 µm). *Leptoconops noei* differs from the new species by the presence of apical pubescence at the spermatheca terminal end, a character absent in *L. communis* sp. nov.

### Remarks on other captured *Leptoconops* species

**Figure 14.**
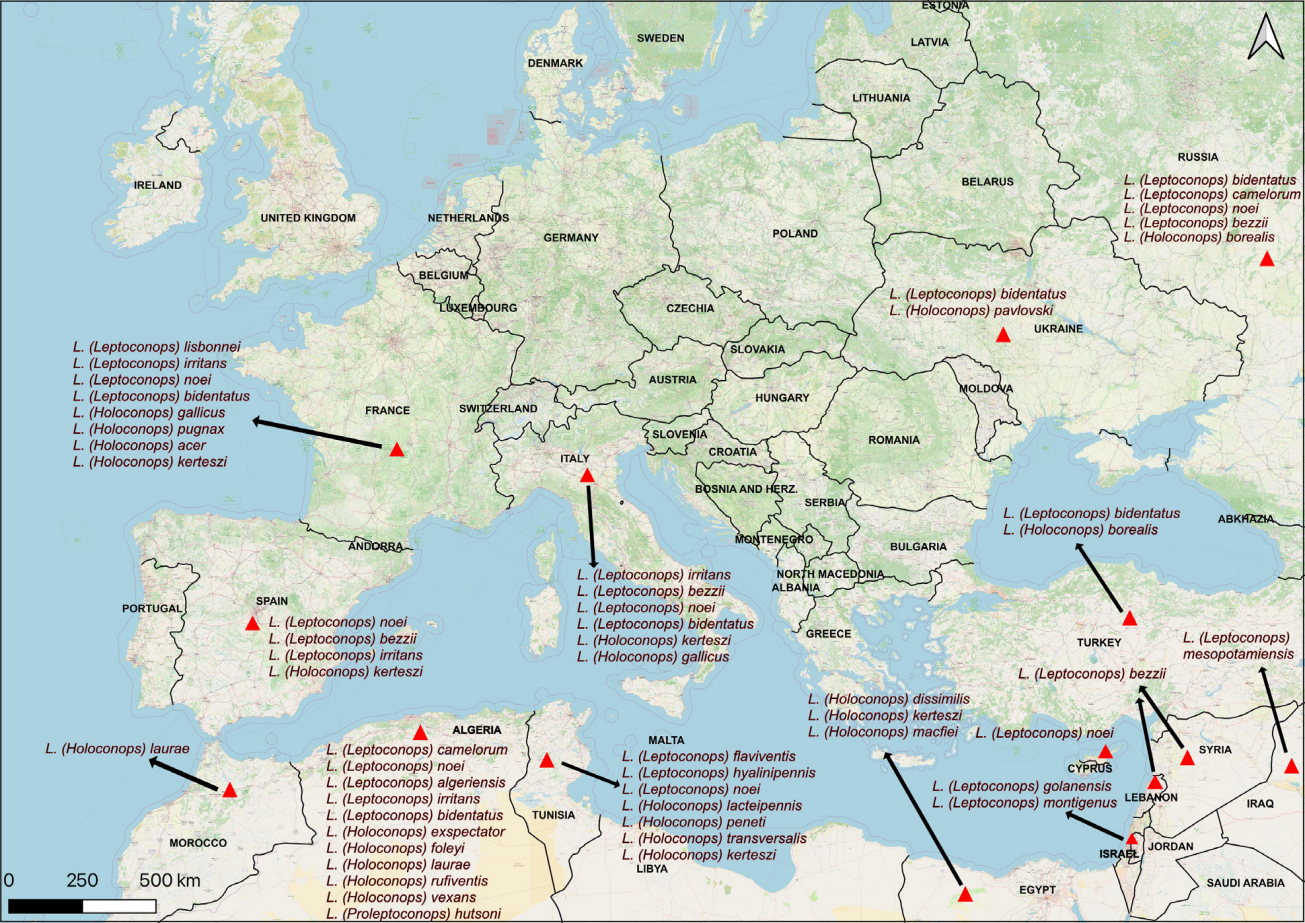
Map illustrating the distribution of Leptoconops species in Europe and surroundings regions (North Africa and Middle East). Locations are approximate and indicate the countries where the species have been recorded. Species in the map are assigned to three subgenera: *L.* (*Leptoconops*), *L.* (*Holoconops*) and *L.* (*Proleptoconops*) as indicated in the labels.

*Leptoconops bidentatus* (*n* = 10 females examined). This study provides the first record of *L. bidentatus* from the Iberian Peninsula, a species originally described by Gutsevich in 1960 from Russia. Since then, it has been reported from the Middle East, North Africa, and Mediterranean Europe (Gutsevich 1960; Clastrier 1972; Soós and Papp 1988; Turgut and Kiliç 2015) (Fig. 14). A few subsequent redescriptions have expanded upon Gutsevich’s original work (Clastrier 1972; Turgut 2011).

Minor differences in body size and morphology are documented in these records, particularly in the length of the third palpal segment, the antennal ratio, and size of the spermathecae, all of which suggests that considerable intraspecific variability occurs throughout this species’ range. Our specimens showed an unusually high variation in the number of bristles on the genital atrium (4–8, typically 4 or 6), a feature not observed in material from Turkey and Russia.

This species is the only known *Leptoconops* species in the Palearctic region with bidentate claws (Fig. 10K). Gutsevich also measured the length of the second spur of the hind tibial comb, a character that is shared with other European congeners. Additional diagnostic characters include its small size, short proboscis, shiny dark-brown mesonotum contrasting with the yellow abdomen (Fig. 10A, B), uniformly pale legs (Fig. 10A), bicolored palps, and the small third palpal segment bearing only a few sensory pits (Fig. 10E, Suppl. material 1: table SS1). No males were collected in our study.

*Leptoconops irritans* (*n* = 10 females examined). This represents the second formal record of *L. irritans* from Spain, following a recent record from Menorca (Balearic Islands) (González et al. 2025). It is one of the most widespread and well-known *Leptoconops* species in Europe (Soós and Papp 1988; Polidori et al. 2023; González et al. 2025) and is considered the most troublesome species for humans in Italy (Carter 1921; Fausto et al. 2007). It has also been recorded from North Africa (Algeria) (Soós and Papp 1988) (Fig. 14). Despite being a common species, some morphological features remain inconsistently described. For example, Huttel and Huttel (1952) describe the abdominal terga as dark, whereas Noé (1905) and Carter (1921) report the abdomen as white, becoming isabelline dorsally (Fig. 11A, B). Such discrepancies are not uncommon in early taxonomic works. Until recently, *L. irritans* was the only European (non-African) species with a distinctly elongate and slender terminal palpus segment (116.63 ± 5.16 µm, length), and a proboscis that is markedly longer than the head, two traits that clearly differentiate it from other species (Fig. 11A, D, Suppl. material 1: table SS1). However, the records of *L. pseudoirritans* sp. nov. in Spain show that these two species share these diagnostic features. A single male in poor condition was collected. Male genitalia coincide with the descriptions in the original records.

*Leptoconops noei* (*n* = 2 females examined). This represents the second record of *L. noei* from Spain, following its detection in northern Spain 13 years ago (González et al. 2013). This species has also been recorded from North Africa (Algeria and Tunisia), Cyprus, France, and Italy (Soós and Papp 1988) (Fig. 14). In the present study, only a few specimens were collected. Its distinguishing characters are given in Fig. 12 and Suppl. material 1: table SS1.

Misidentification of *Leptoconops bezzii*. Some *Leptoconops* with mixed-colored legs were initially identified as *L. bezzii*. However, detailed microscopic examination revealed consistent morphological differences that do not match any previously described European species. This led us to describe these specimens as a new species, *Leptoconops communis* sp. nov. Further molecular barcoding has confirmed this hypothesis. *Leptoconops communis* sp. nov. can be distinguished from *L. bezzii* based most notably on the third palpal segment, which is incrassate and bears fewer than 70 sensory pits. Additional distinguishing features include four humeral pits and subapically positioned hyaline sensory sensilla shaped like a bull-horn. The diagnostic characters of *L. bezzii* are illustrated in Fig. 13 and summarized in Suppl. material 1: table SS1.

Although *L. bezzii* had previously been recorded in the Monegros region in northeast Spain (Pedrocchi Renault 1998; Delecolle 2002), and in Córdoba and Cádiz (south Spain) (Obregón et al. 2019; Borkent 2024) it was not recorded in our survey. In Europe, *L. bezzii* is widely distributed (Rioux and Descous 1966; Clastrier and Coluzzi 1973; Gutsevich 1973) (Fig. 14).

### *Leptoconops* species distribution in Europe and surroundings regions

Eleven species are currently recognized in Europe. The distribution of *Leptoconops* species in Europe and surrounding regions, shows a clear concentration of species in the Mediterranean basin, including southern Europe (Spain, France, Italy, and the Balkans) and North Africa (Morocco, Algeria, Tunisia, and Egypt). Several species also occur in the Middle East (Turkey, Cyprus, Lebanon, and surrounding areas), while comparatively fewer records are known from central and eastern Europe and western Russia (Fig. 14).

Based on the available records, species of *L.* (*Leptoconops*) appear to be the most widely distributed subgenus, whereas *L.* (*Holoconops*) is mainly represented in Mediterranean regions. In contrast, *L.* (*Proleptoconops*) is represented by very few records (Fig. 14).

## Discussion

We provide here the first comprehensive characterization of Leptoconops (Leptoconops) species in Spain within a broader European context. The current understanding of *Leptoconops* diversity in Europe remains limited compared to other hematophagous ceratopogonid genera such as *Culicoides*. Most taxonomic knowledge of this genus was generated during the late nineteenth and twentieth centuries, particularly in France, Italy, and Russia. Subsequently, species occurrence within the subgenera *L.* (*Holoconops*) was clarified by Clastrier (1973) and in *L.* (*Leptoconops*) by Gutsevich (1960, 1973), Clastrier (1972), and Clastrier and Coluzzi (1973). More recently, some studies have attempted to refine the morphological differences using molecular techniques and electronic microscopy analysis (Polidori et al. 2023; Naro et al. 2025). However, accurate species identification within this genus remains a challenge.

In our study, confident species identification was hindered by several taxonomic issues including long-standing synonymies (Huttel and Huttel 1952; Dzhafarov 1962), inconsistencies in morphological descriptions among authors (Carter 1921; Gutsevich 1960; Clastrier 1972; Clastrier and Coluzzi 1973), and, in many cases, the limited accessibility of historical literature. Additionally, the assignment of females to their conspecific males remains a persistent source of confusion. Many identification keys are severely outdated and need a major revision if an updated species list is to be generated; without such a list, reliable identification will remain extremely difficult. We also detected notable discrepancies in the description of some morphological characters for the same species (Carter 1921; Clastrier 1972; Clastrier and Coluzzi 1973; Gutsevich 1973). For example, thoracic coloration appears to have been interpreted differently by various authors, probably due to observational bias provoked by differing lightning conditions or specimen preservation methods. Several characters show high levels of intraspecific variation (Carter 1921), whereas others, such as the palpus, are strongly affected by the slide orientation or viewing angle (Kieffer 1908; Clastrier and Wirth 1978).

Our comparative analysis of the specimens examined reveals clear morphological differences between the new species described here and previously documented species from North Africa, Western Asia (including Caucasus region), Central Asia, and the Middle East. These differences primarily involve palpal morphology, coloration patterns on the mesonotum and tarsomeres, the shape of the antennal flagellomeres, the associated arrangement of the sensilla, and the presence or absence of a third accessory spermatheca.

Despite these challenges, our study provides the most detailed and up-to-date assessment of *Leptoconops* diversity in Europe. It has enabled us to compile a revised and annotated list of all European species of the subgenus *L.* (*Leptoconops*), describe several new species, and produce over 30 high-quality taxonomic plates (see Supplementary Material). We also provide an updated key, together with thorough morphological features and morphometric measurements of all known species (*L. bidentatus*, *L. noei*, *L. irritans* and *L. bezzii*). Furthermore, COX1 barcodes are provided to facilitate reliable molecular identification in future faunistic and medical entomology studies. Such sequencing will also allow confident association between males and females and between adults and immatures. While *L. irritans* and *L. pseudoirritans* sp. nov. are very similar morphologically, COX1 barcodes further support them as different taxa. The similarity within the three available *L. irritans* sequences ranged between 97.87 and 99.85% and between 99.54 and 99.85% for the four *L. pseudoirritans* sp. nov. sequences. However, similarities between *L. irritans* and *L. pseudoirritans* sp. nov. sequences ranged between 84.04% and 85.11%.

Our study is based exclusively on female specimens, as males are rarely captured using CO_2_-baited traps. Males can instead be collected by aspiration at their resting sites (e.g., on vegetation or soil) or by aerial net-sweeping, emergence traps, or Malaise traps (Clastrier 1972). Further exhaustive research is also needed to define the ecological niche of each species and to identify oviposition substrates and larval development sites. Finally, understanding the biology of the newly described species - including their biting activity, phenology, and host-feeding preferences - will be essential for elucidating the ecology and potential epidemiological relevance of *Leptoconops* species in Europe.

## Conclusions

This study represents the first comprehensive characterisation of European *Leptoconops* species belonging to the subgenus *L.* (*Leptoconops*) and represents a significant contribution to the current incomplete taxonomic knowledge of this genus. We document eight species from southwest Spain, including four newly described taxa, and provide detailed accounts of their morphological traits supported by high-quality images. Our findings reveal a previously unrecognized level of diversity within this genus in Europe. An updated and comprehensive identification key presented here will facilitate accurate species determination and support future research on the ecology, distribution, and potential medical and veterinary relevance of *Leptoconops*. This work establishes a robust foundation for further studies of the biology, phenology, and habitat requirements of these still poorly known biting midges.

## Supplementary Material

XML Treatment for Leptoconops nigrithorax

XML Treatment for Leptoconops triangularis

XML Treatment for Leptoconops pseudoirritans

XML Treatment for Leptoconops communis

## Additional information

### Artificial Intelligence (AI) use

The authors accept full responsibility for the content of the manuscript, including the disclosure of any use of AI.

No AI tools were used in the preparation of this manuscript.

### Author ORCIDs

D.I. Paun-Tanase https://orcid.org/0009-0006-9113-8858

M.A. González https://orcid.org/0000-0002-5754-862X

S. Magallanes https://orcid.org/0000-0002-6748-9547

G. Naro https://orcid.org/0009-0006-4732-9649

S. Epis https://orcid.org/0000-0001-8807-4802

J. Figuerola https://orcid.org/0000-0002-4664-9011

### Data availability

All of the data that support the findings of this study are available in the main text or Supplementary Information. Photographs of species plates in high resolution are uploaded in Zenodo under the following doi: https://doi.org/10.5281/zenodo.19950325.

## Supplementary materials

Supplementary material 1 *Leptoconops* taxonomy Data type docx Explanation note In this document is available all suplementary taxonomic images from the species, and also three tables with information about taxonomic features. https://binary.pensoft.net/file/1629961This dataset is made available under the Open Database License (http://opendatacommons.org/licenses/odbl/1.0/). The Open Database License (ODbL) is a license agreement intended to allow users to freely share, modify, and use this Dataset while maintaining this same freedom for others, provided that the original source and author(s) are credited. Dumitru Ionut Paun-Tanase, Mikel Alexander González, Sergio Magallanes, Giovanni Naro, Sara Epis, Jordi Figuerola
