# Supplementary material for: Hidden diversity in Europe: new species and a revised taxonomy of the subgenus Leptoconops (Leptoconops) (Diptera, Ceratopogonidae)
Source: Zookeys. 2026 May 8;1279:201–39. doi: 10.3897/zookeys.1279.186179 (PMC13179505; doi:10.3897/zookeys.1279.186179)
Supplement: Supplementary material 1 — Leptoconops taxonomy [file zookeys-1279-201_article-186179__-s001.docx]

**Hidden diversity in Europe: New species and a revised taxonomy of the subgenus *Leptoconops* (*Leptoconops*) (Diptera: Ceratopogonidae)**

**Dumitru Ionut Paun-Tanase^1,2^*^,a^, Mikel Alexander González^1,2^*^,b^, Sergio Magallanes^1,2^, Giovanni Naro^3^, Sara Epis^3^, Jordi Figuerola^1,2^**

^1^Department of Conservation Biology and Global Change, Biological Station of Doñana (EBD, CSIC), Avda. Américo Vespucio 26, 41092, Seville, Spain.

^2^CIBER de Epidemiologia y Salud Pública (CIBERESP), Av. Monforte de Lemos, 3-5. Pabellón 11. Planta 0, 28029 Madrid, Spain

^3^Department of Biosciences and Pediatric Clinical Research Center “Romeo and Enrica Invernizzi”, University of Milan, Milan, Italy

**** Both authors contributed equally***

***^a^ Corresponding author****:* Dumitru Ionut Paun-Tanase. Avda. Américo Vespucio 26, 41092, Sevilla, Spain. E-mail: [dumitrui.paun@ebd.csic.es](mailto:dumitrui.paun@ebd.csic.es)

***^b^ Corresponding author****:* Mikel Alexander González. Avda. Américo Vespucio 26, 41092, Sevilla, Spain. E-mail: [mikel_alexander86@hotmail.com](mailto:mikel_alexander86@hotmail.com)


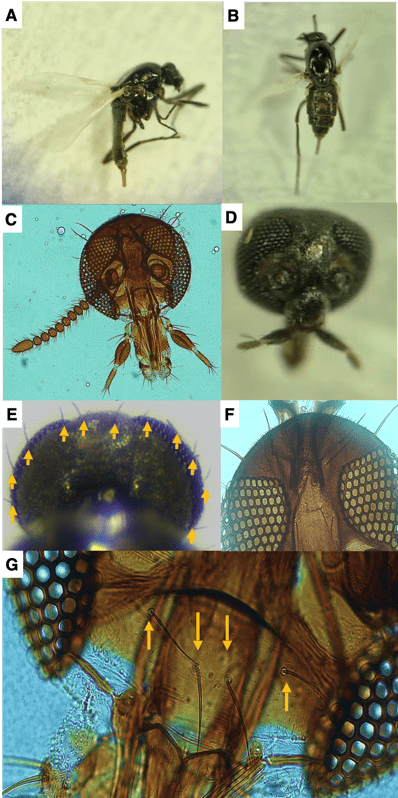
***Figure S1***. Habitus and head of *Leptoconops nigrithorax* sp. nov. A. Lateral view (FS), B. Dorsal view (FS), C. Head (MS), D. Head (FS), E, F. Vertex with setae distribution pointed by yellow arrows (MS), G. Arrangement of setae pointed by yellow arrows. on fronto-clypeus (MS)

***Figure S2.*** Antenna of *Leptoconops nigrithorax* sp. nov. A. Flagellomeres III - XIV (FS) B. Flagellomeres VI - XIV (FS) C. Terminal segment (MS)


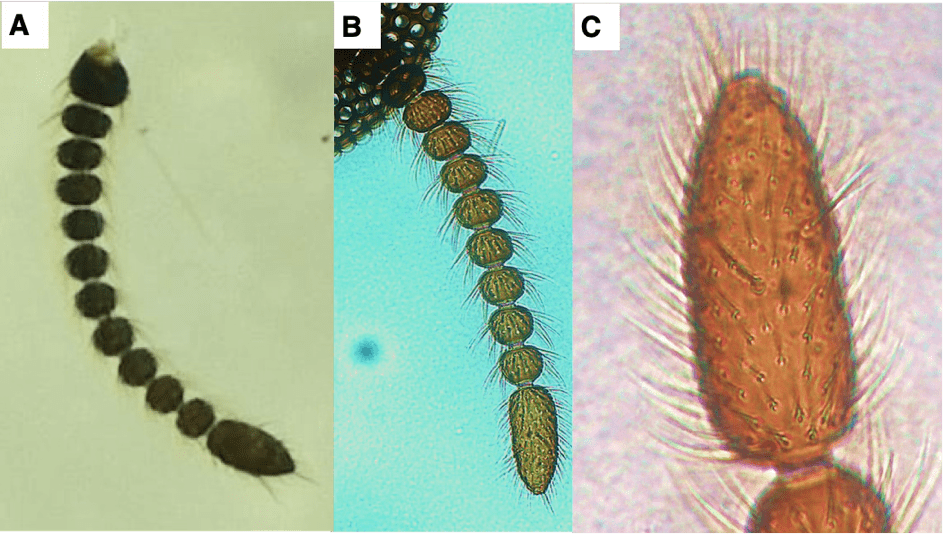


***Figure S3***. Maxillary palp of *Leptoconops nigrithorax* sp. nov. A. Palpus (FS), B. Maxilla (MS), C. Mandible (MS).


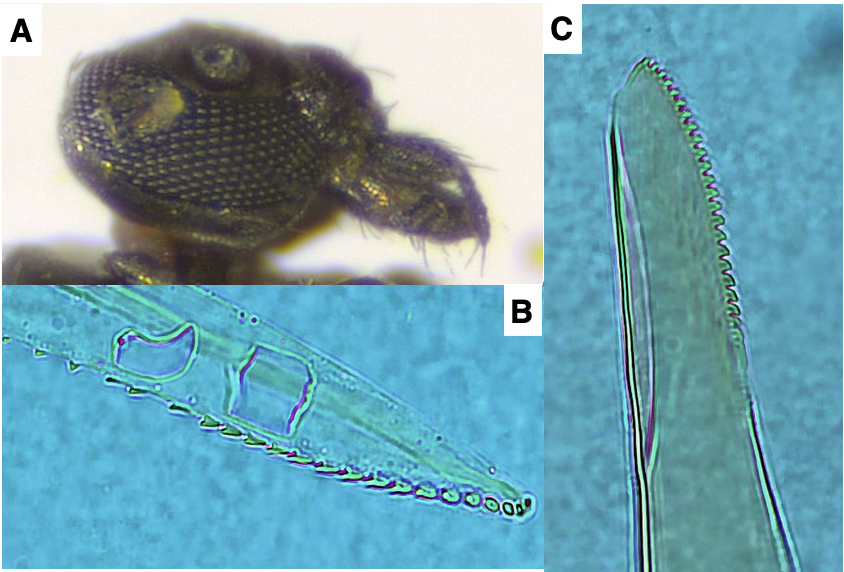


***Figure S4***. Thorax of *Leptoconops nigrithorax* sp. nov. A. Mesonotum (FS), B. Pleuron (FS), C, D. Dorsal chaetotaxy (FS), E. Scutellum setae distribution pointed by yellow arrows (MS), F. Pre-scutellum and halters (MS).


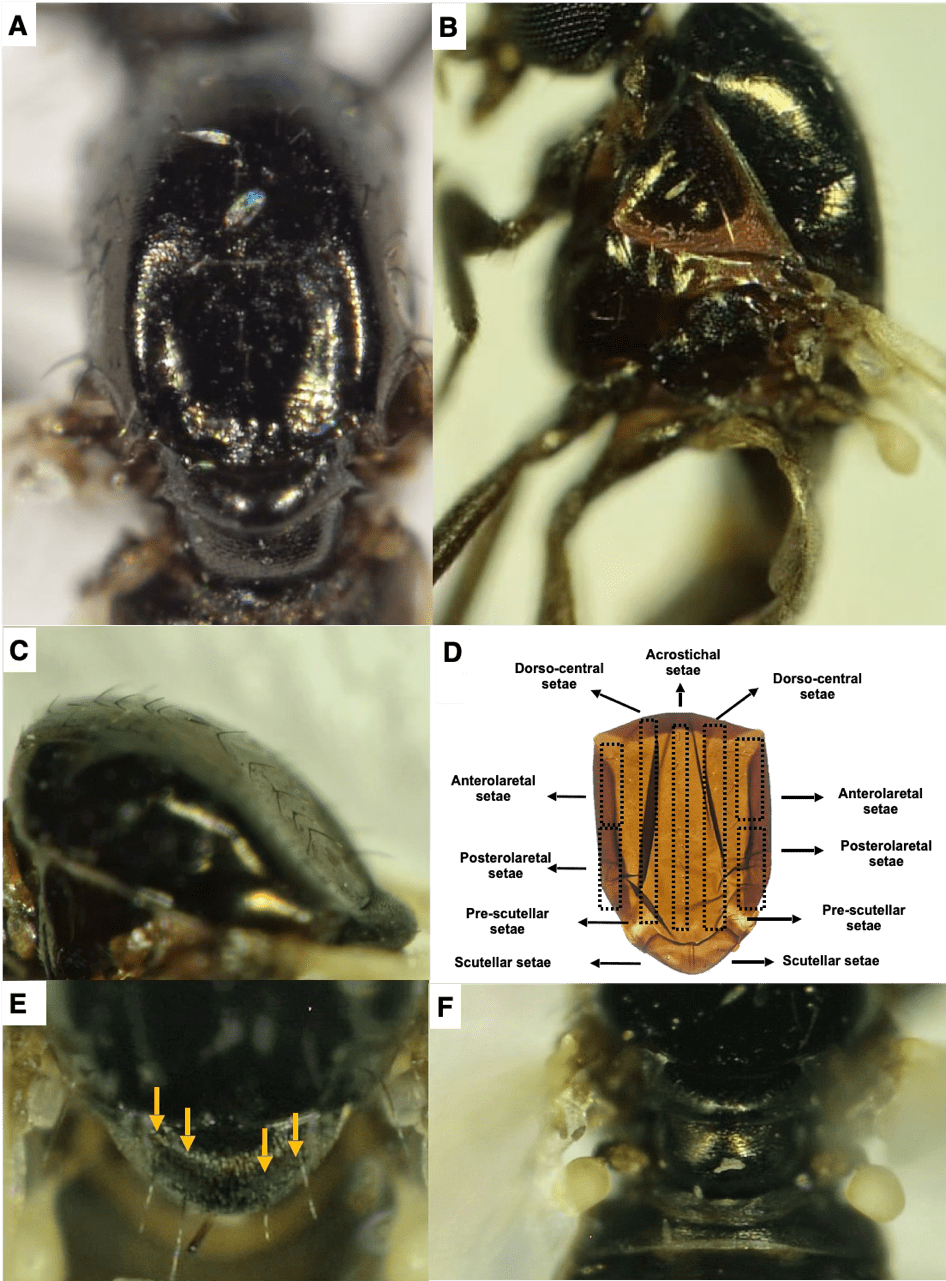


***Figure S5***. Legs *of Leptoconops nigrithorax* sp. nov. A. Tibial comb (MS), B, C. Claws (MS), D. Coxa and femur chordotonal organs pointed by yellow arrows (MS), E. Wing (FS).


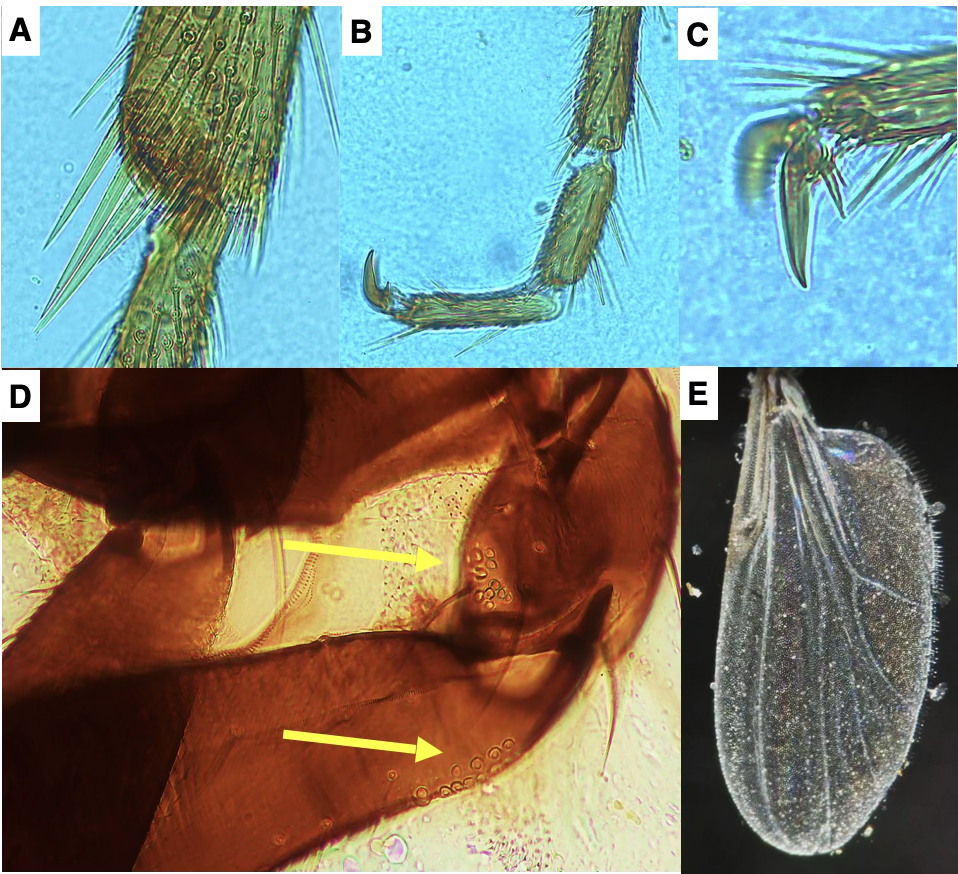


***Figure S6***. Abdomen of *Leptoconops nigrithorax* sp. nov. A. Tergite (FS), B. Sternite (FS), C. Ventral view of sternite VIII and lamella cercus (MS), D. Main spermatheca (one visible) (MS), E. Anal armature (MS).


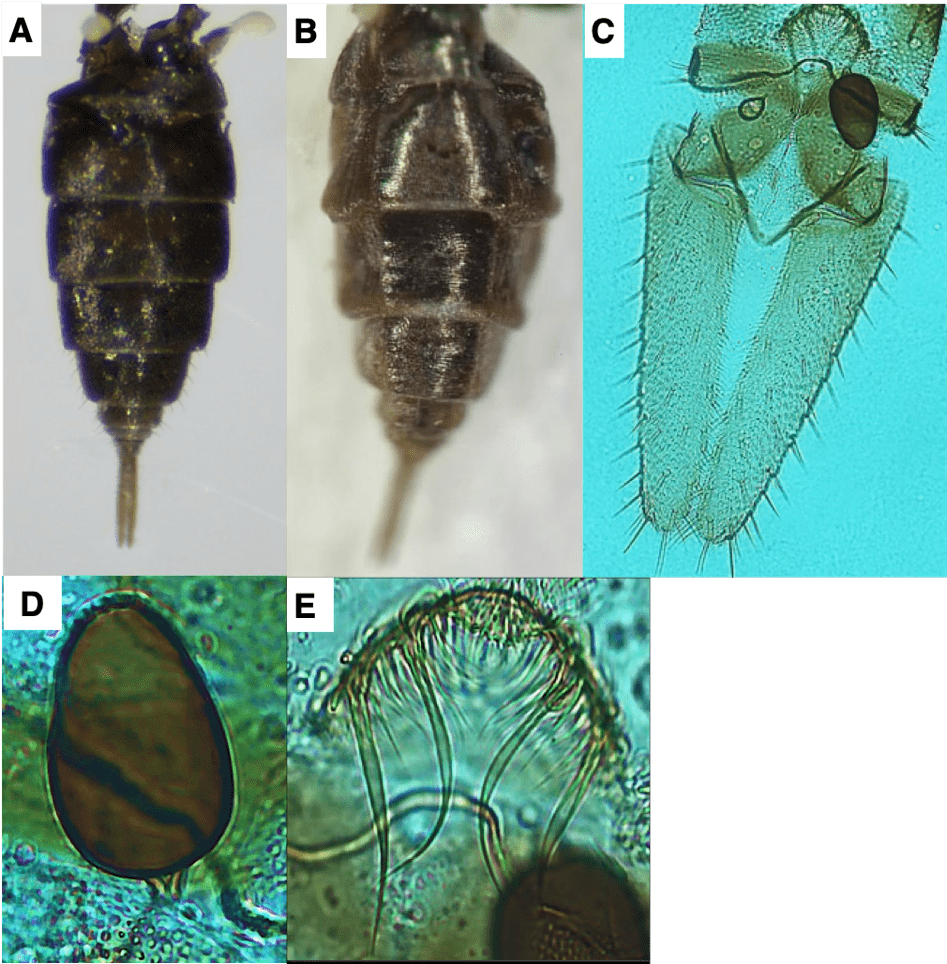


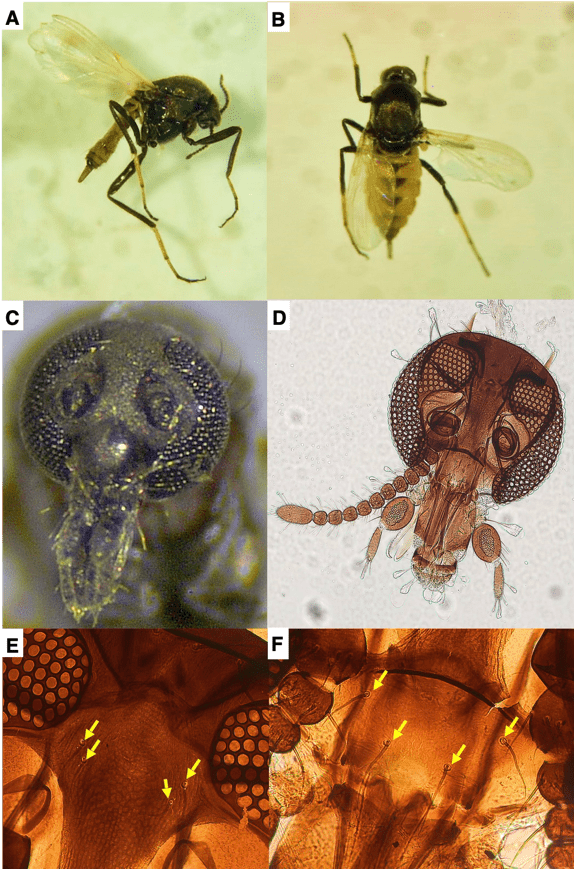
***Figure S7***. Head of *Leptoconops triangularis* sp. nov. A. Lateral view (FS), B. Dorsal view (FS), C. Head (FS), D. Head (MS), E. Interocular space, yellow arrows pointing setae (MS), F. Arrangement of setae pointed by yellow arrows on fronto-clypeus (MS).

***Figure S8***. Antenna of *Leptoconops triangularis* sp. nov. A. Whole antenna (MS) B., Terminal segment (MS), C. Sensilla trichodea (MS), D. Sensilla chaetica (MS), E. Hyaline sensory setae (MS).


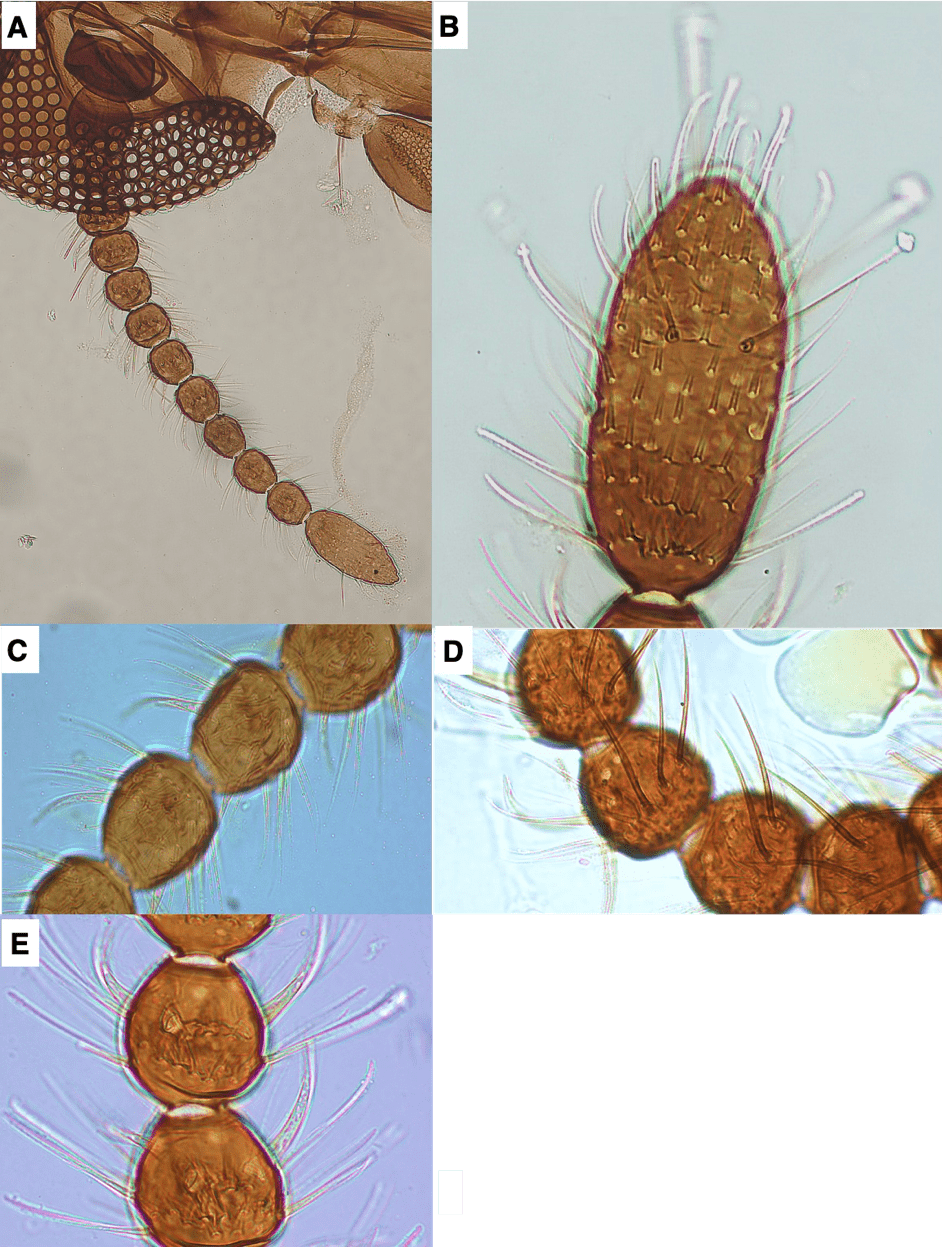


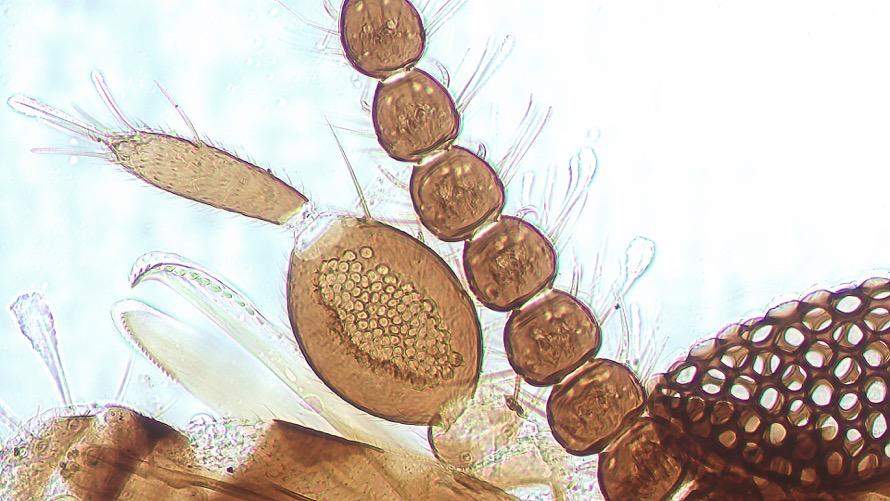
***Figure S9***. Mouthparts of *Leptoconops triangularis* sp. nov (MS).

***Figure S10***. Thorax of *Leptoconops triangularis* sp. nov. A. Mesonotum (FS), B, C. Prescutellum pits, humeral pits, and halters (FS).


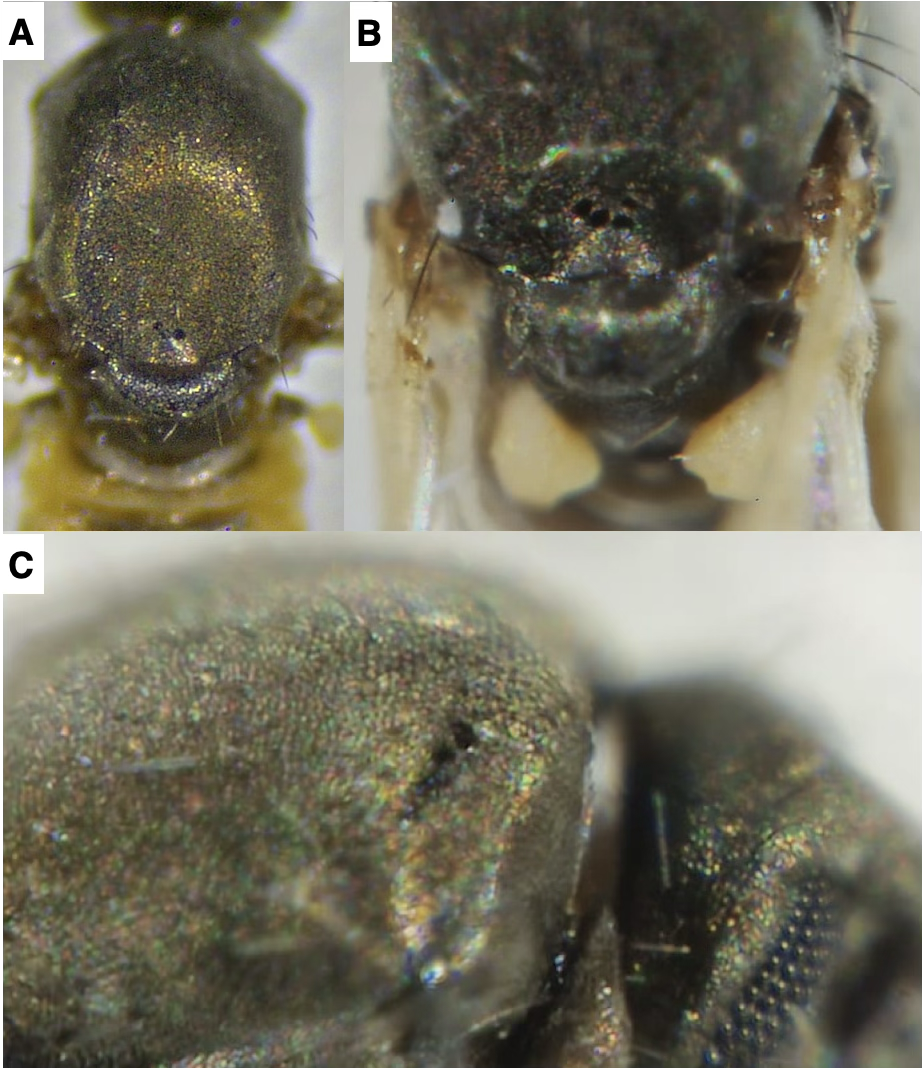


***Figure S11***. Legs of *Leptoconops triangularis* sp. nov. A. Tibial comb (MS), B, C. Claws (MS), D, E. Wing (FS).


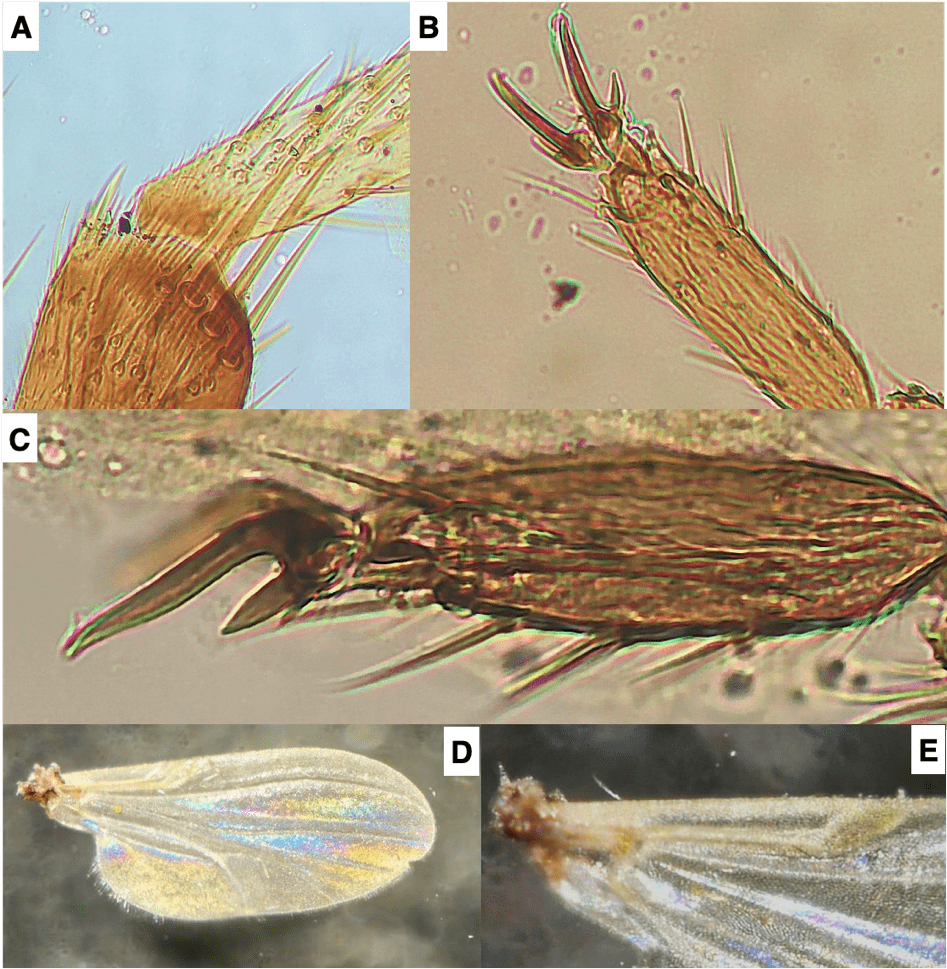


***Figure S12***. Abdomen of *Leptoconops triangularis* sp. nov. A., Ventral view of sternite VIII and lamella (MS), B. Lateral view of sternite VIII (MS), C. Anal armature (MS), D. Main spermathecae (MS).


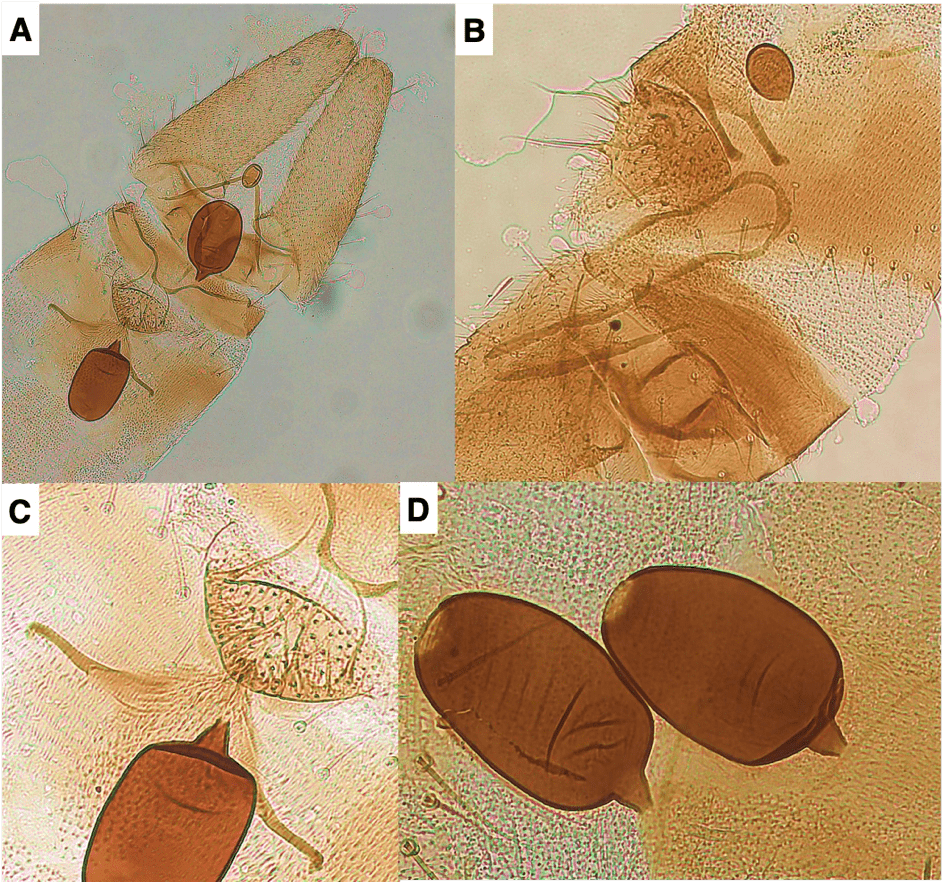


***Figure S13***. Habitus and head of *Leptoconops pseudoirritans* sp. nov. A. Dorsal view (FS), B. Head (FS) C, D. Proboscis (FS), E. Arrangement of the vertex setae (MS).


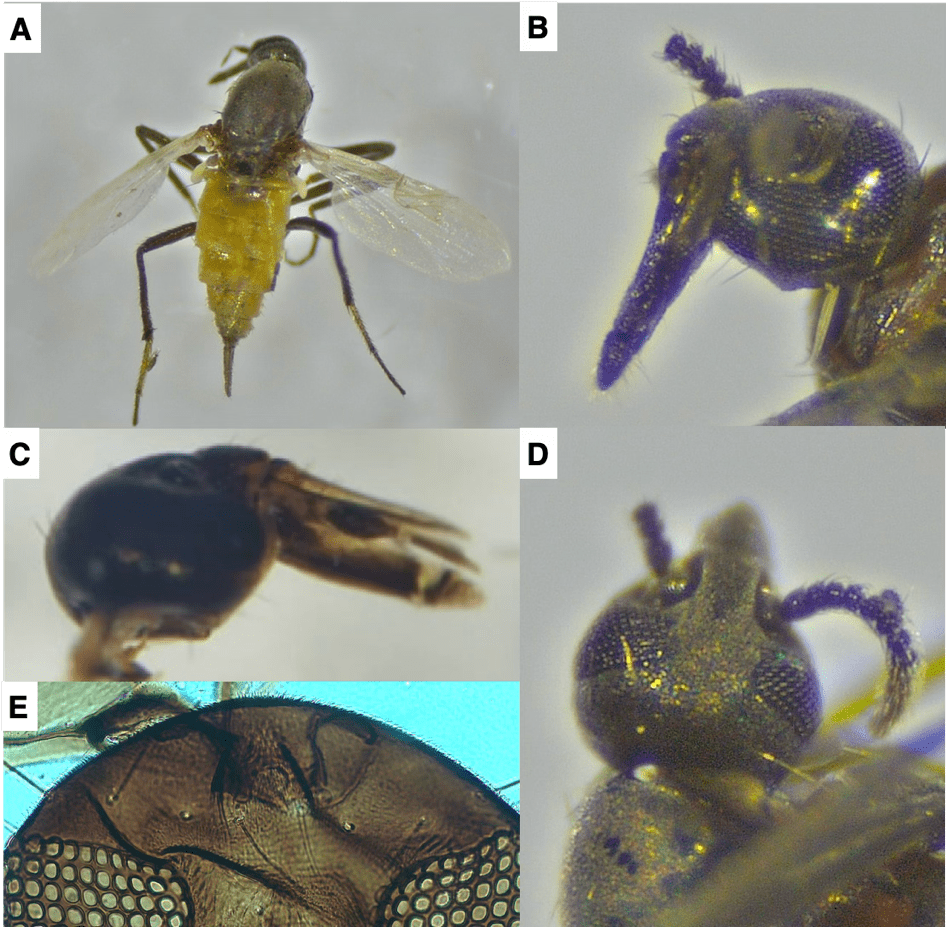


***Figure S14***. Antenna of *Leptoconops pseudoirritans* sp. nov. A. Flagellomeres IV - XIV (MS) B. Terminal segment (MS), C. Hyaline sensory setae (MS).


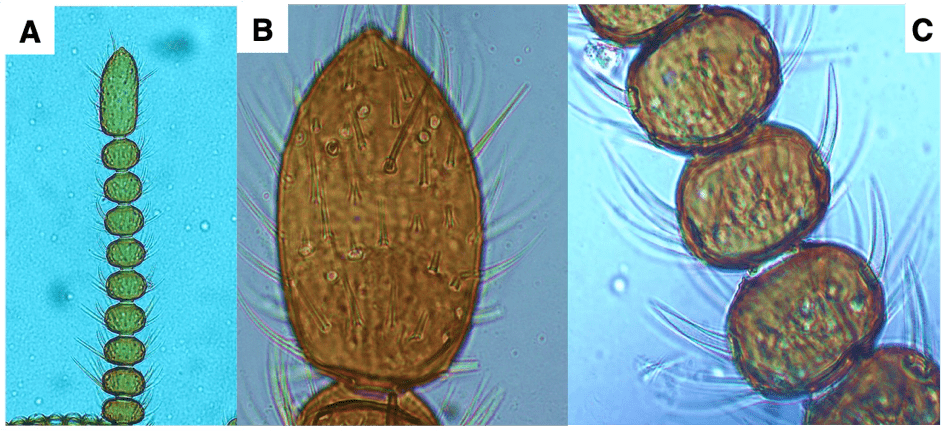


***Figure S15***. Maxillary palp of *Leptoconops pseudoirritans* sp. nov. A. General habitus (MS), B. Palpus (MS), C. Ventral view of 3^rd^ palpal segment (MS), D. Maxilla (MS), E. Mandible (MS).


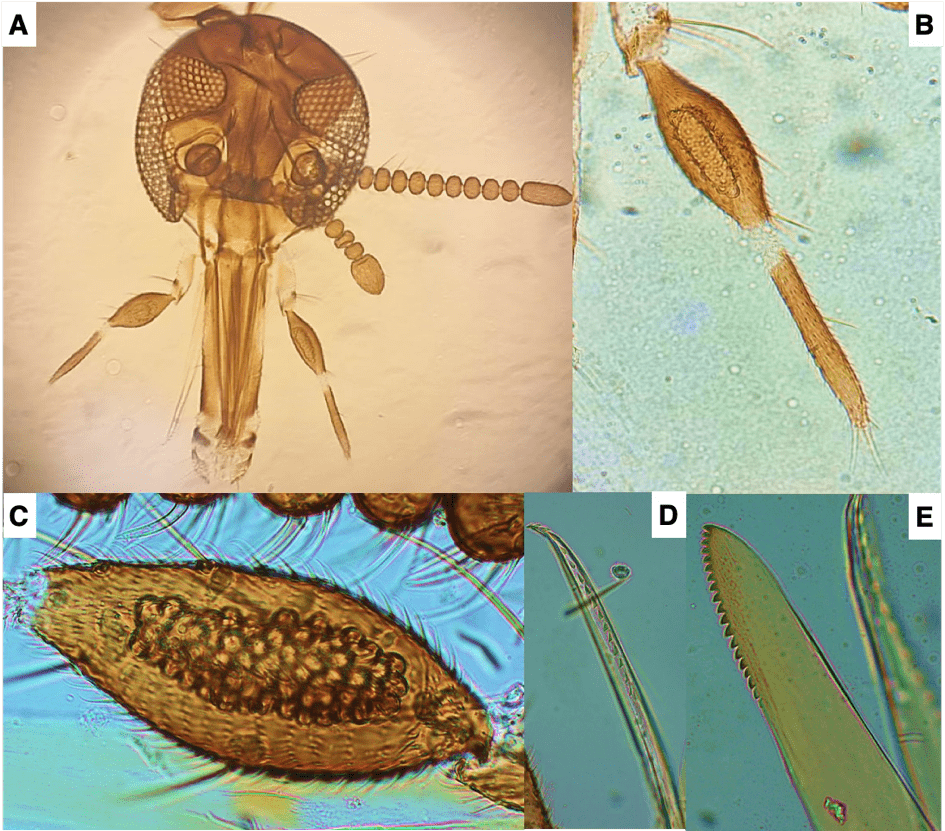


***Figure S16***. Thorax of *Leptoconops pseudoirritans* sp. nov. A. Mesonotum, (FS) B. Humeral pits (FS), C. Pre-scutellum pits (FS), D. Halters pointed by yellow arrow (FS).


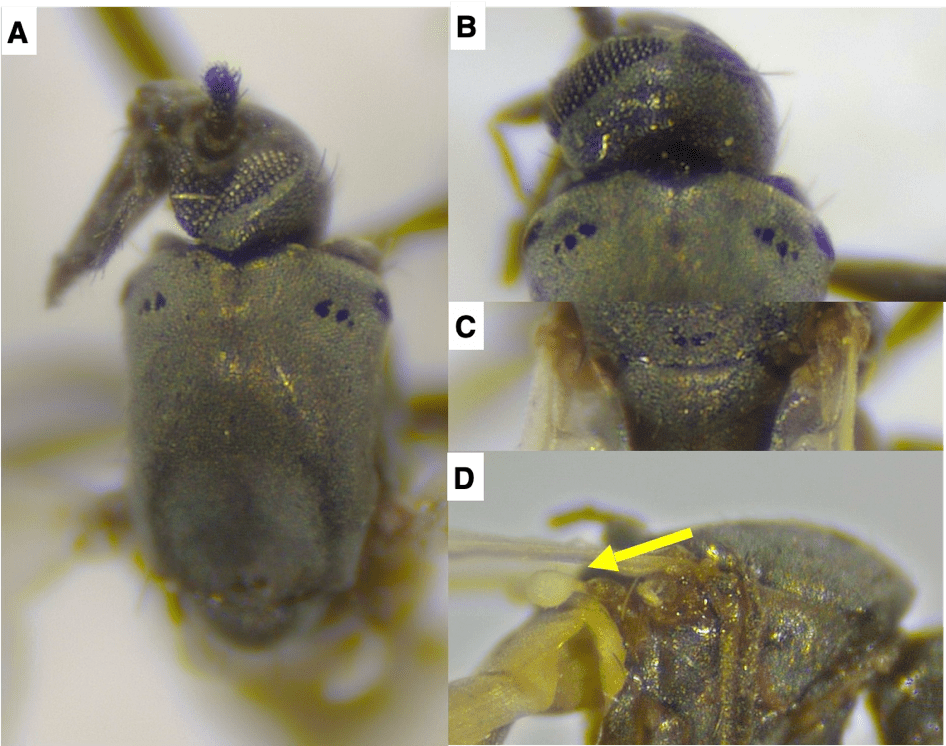


***Figure S17***. Legs of *Leptoconops pseudoirritans* sp. nov. A. Tibial comb (MS), B. Tibial sensory area (MS), C. Chaetotaxy of femur and tibia (MS), D, E, F. Claws (MS), G. Wing (FS).


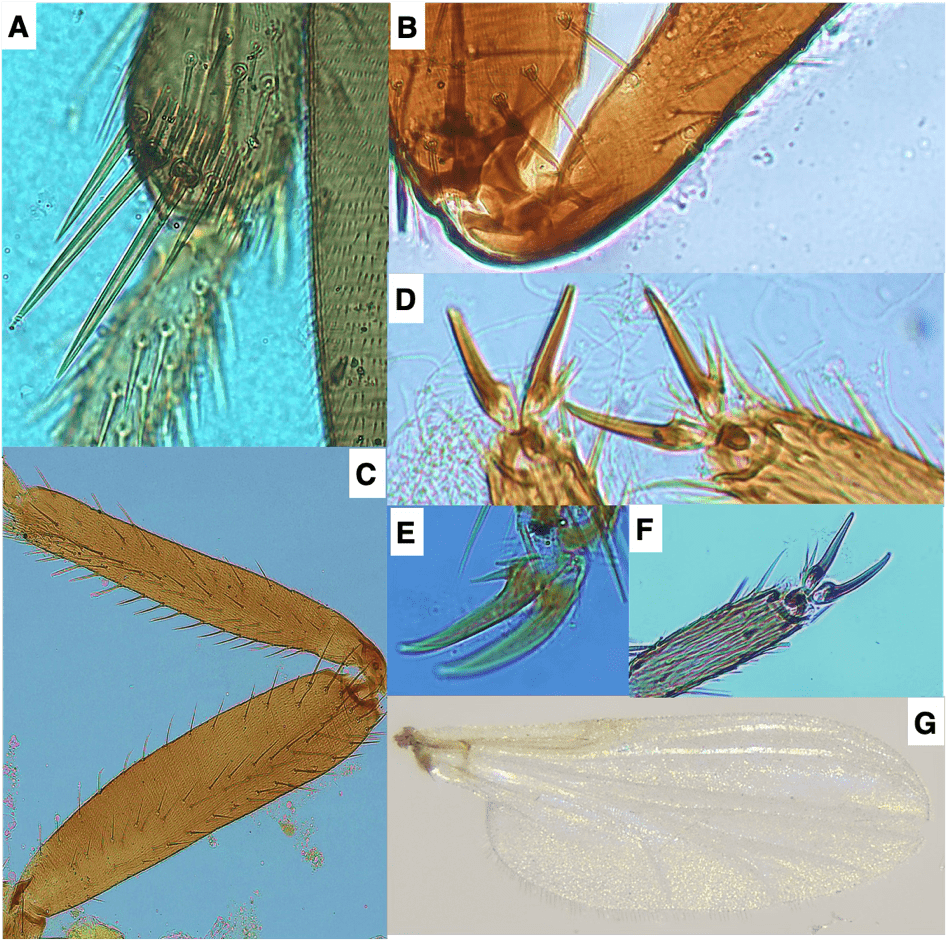


***Figure S18***. Abdomen of *Leptoconops pseudoirritans* sp. nov. A. Tergite (FS), B. Sternite VIII and lamella (MS), C. Lateral view of anal armature (MS), D. Ventral view of anal armature (MS).


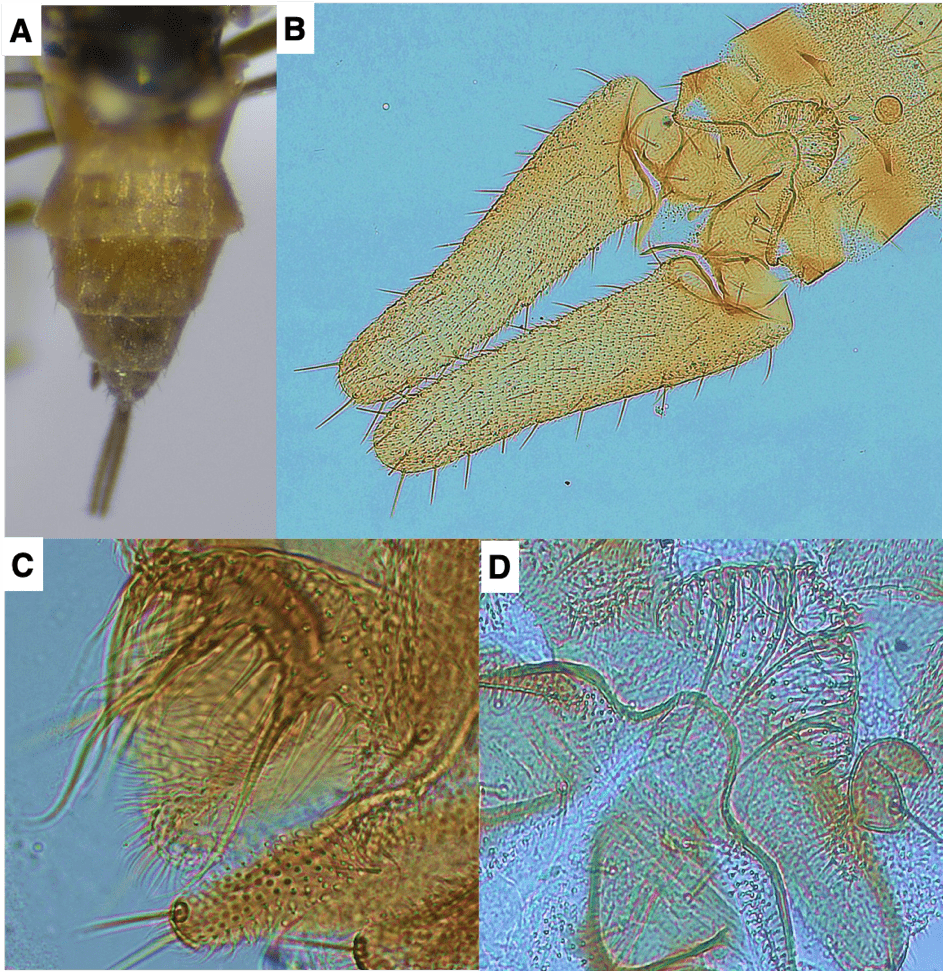


***Fig S19***. Habitus and head of *Leptoconops communis* sp. nov. A. Lateral view (forelegs and antennae missing) (FS), B. Head (MS), C. Head in lateral and dorsal view, respectively (FS), D. Vertex setae (pointed by yellow arrows) (MS).


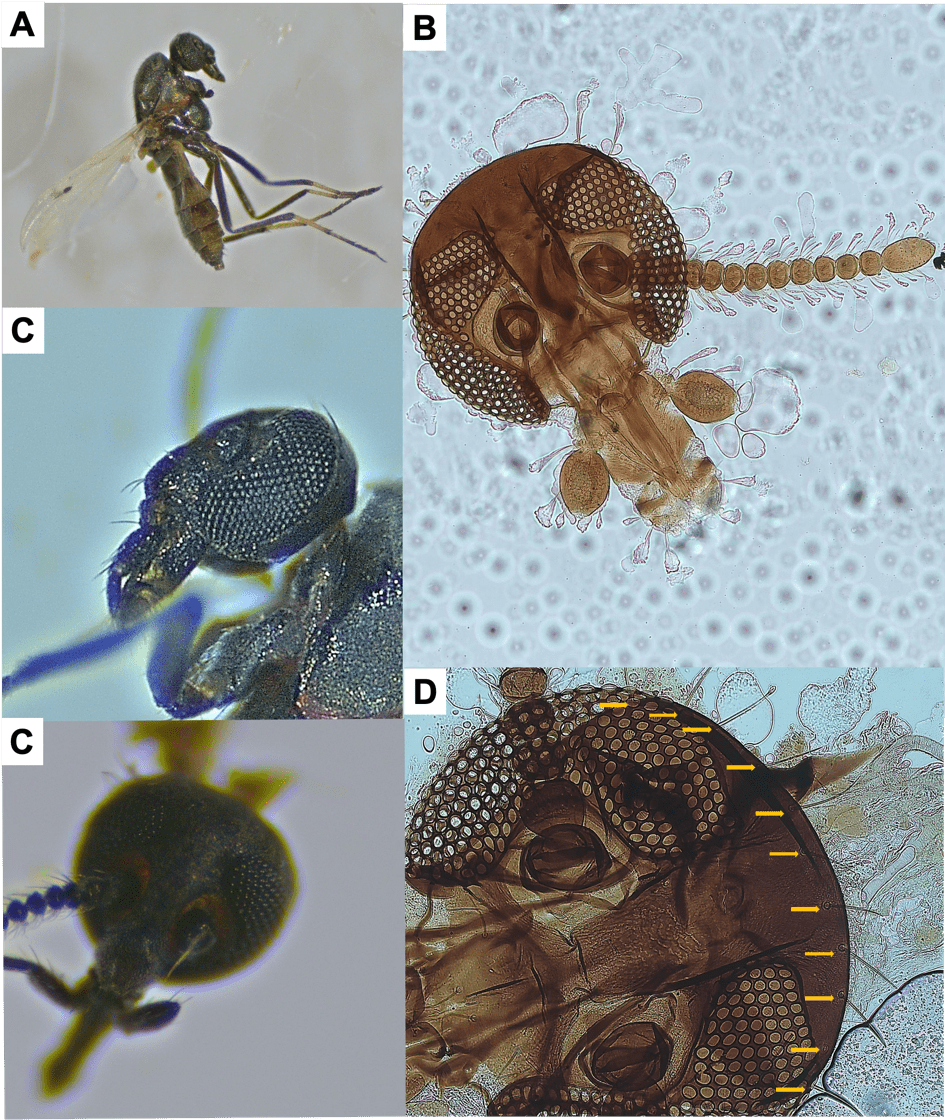


***Figure S20***. Antenna of *Leptoconops communis* sp. nov. A. Whole antenna (MS), B. Whole antenna (MS); C. Pedicel setae (MS), D. Terminal flagellomere setae (MS), E. Sensilla chaetica and sensilla trichodea (MS).


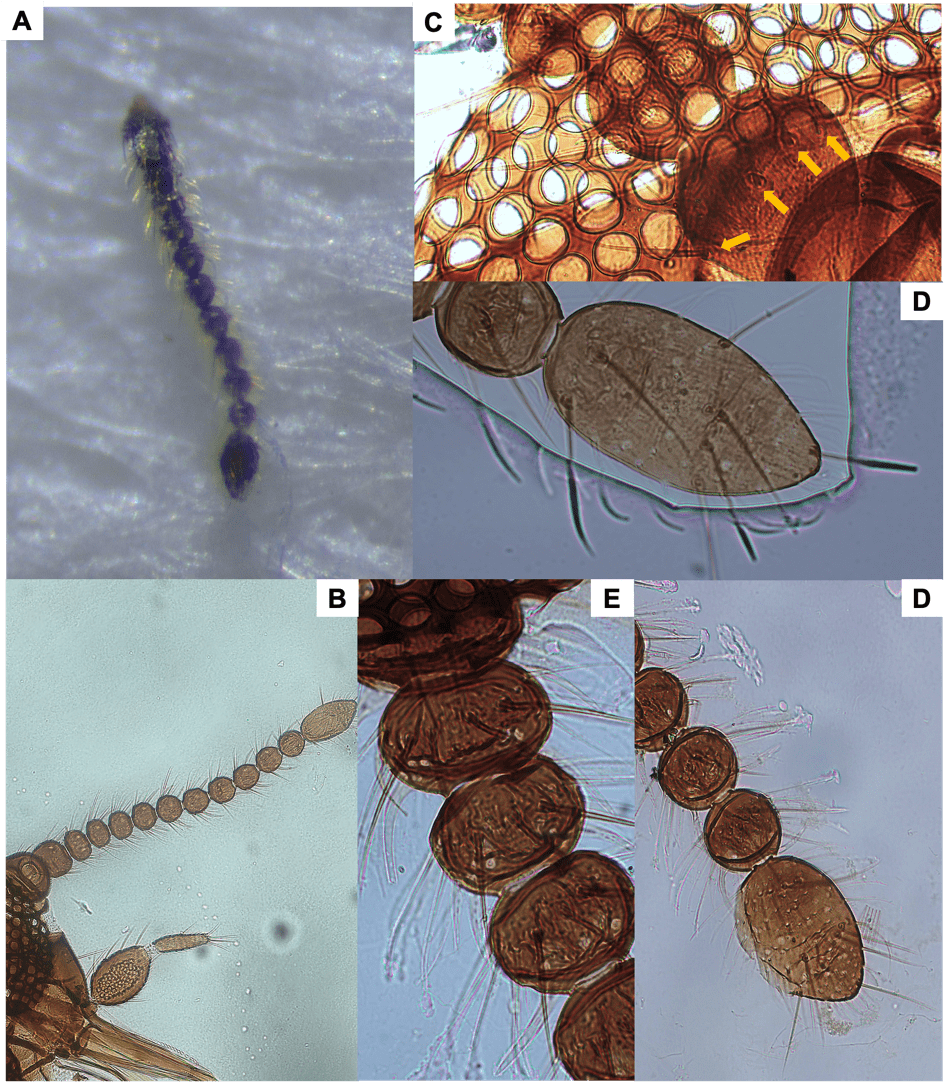


***Figure S21***. Maxillary palp of *Leptoconops communis* sp. nov. A. Palpus (FS), B. Ventral view of 3^rd^ palpal segment (MS), C. 4^th^ palpal segment setae (MS), D. Mandible (MS), E. Maxillae (MS).


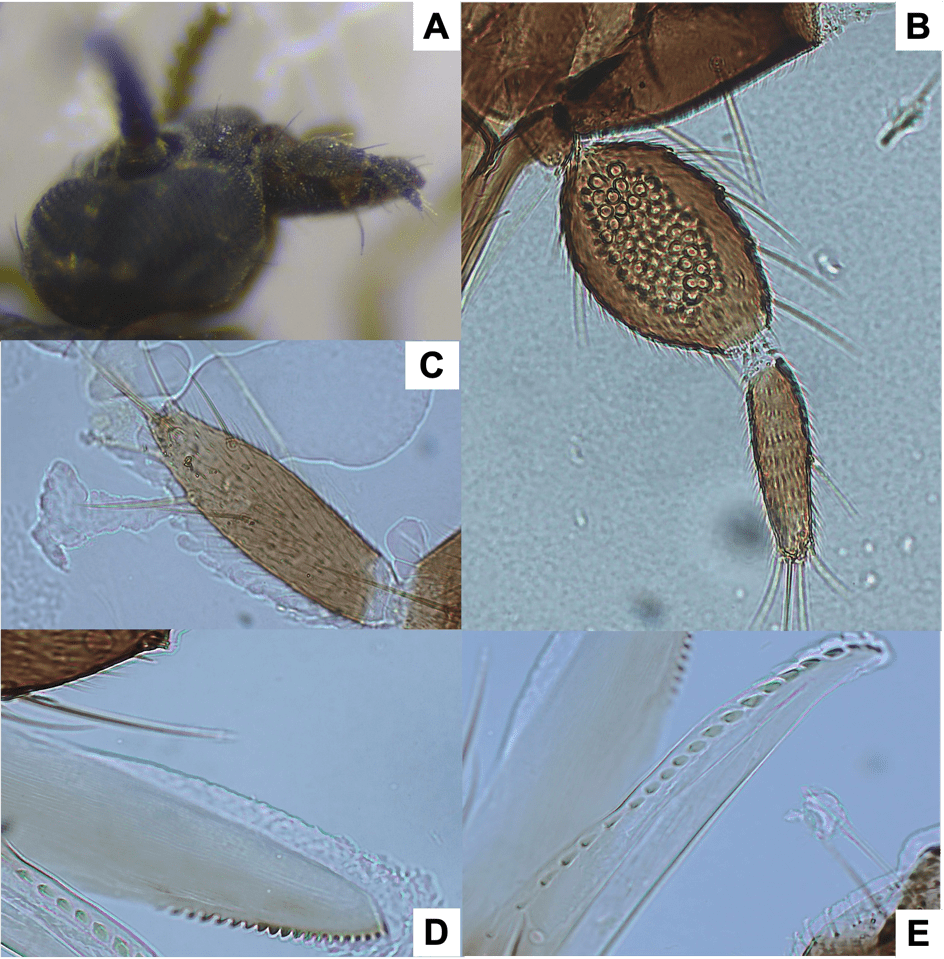


***Figure S22***. Thorax of *Leptoconops communis* sp. nov. A. Halters (FS), yellow arrows indicate the position of B. humeral pits (FS), C. humeral callus setae (FS), D. scutellum setae (FS); E. Anepisternal cleft (FS).


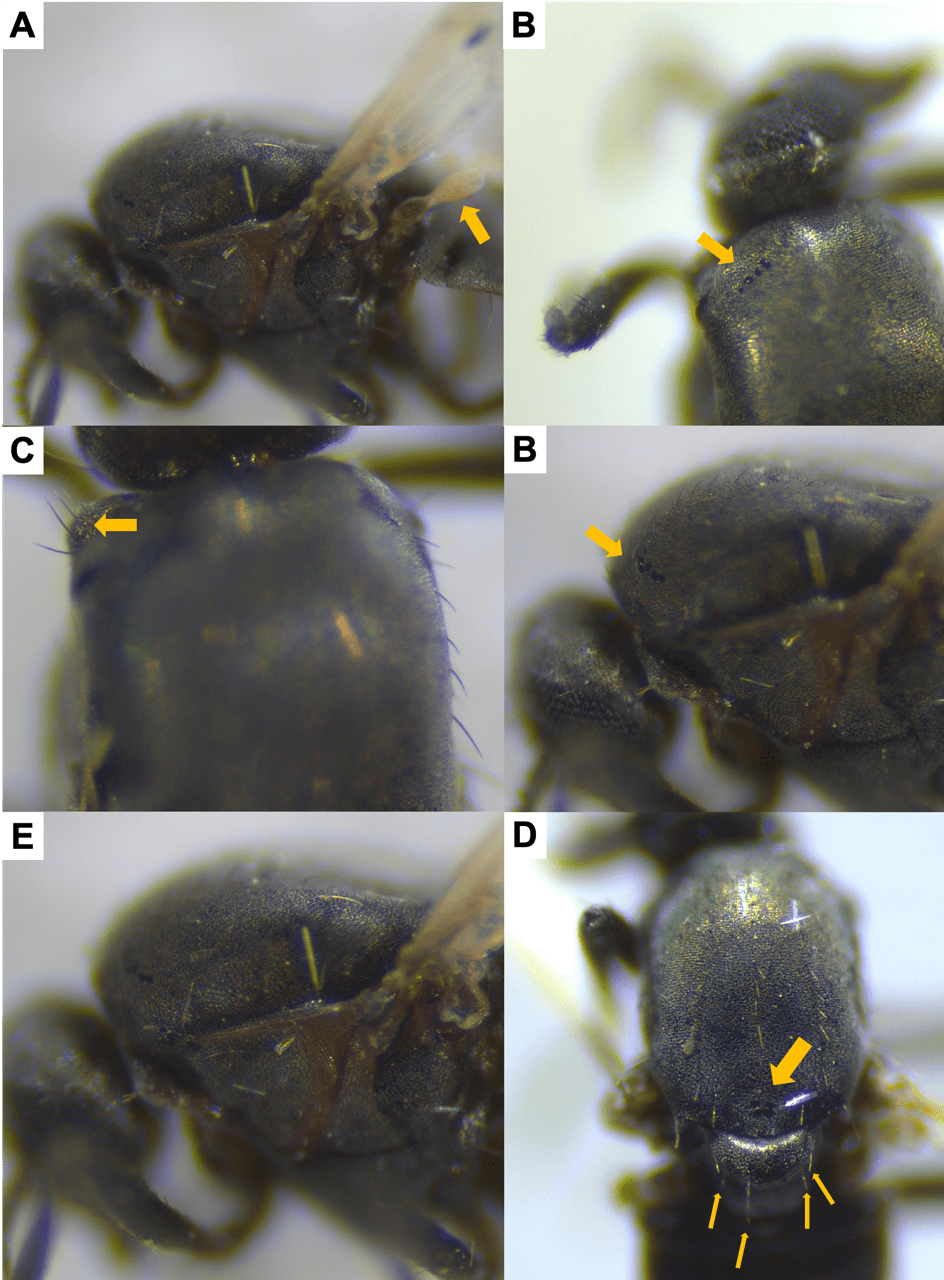


***Figure S23***. Legs of *Leptoconops communis* sp. nov. A. Tibial comb (MS), B. Coxa chordotonal organs (pointed by arrow) (MS), C. Femur chordotonal organs (pointed by arrow) (MS), D. Coxa setae (MS), E. Trochanter setae (MS).


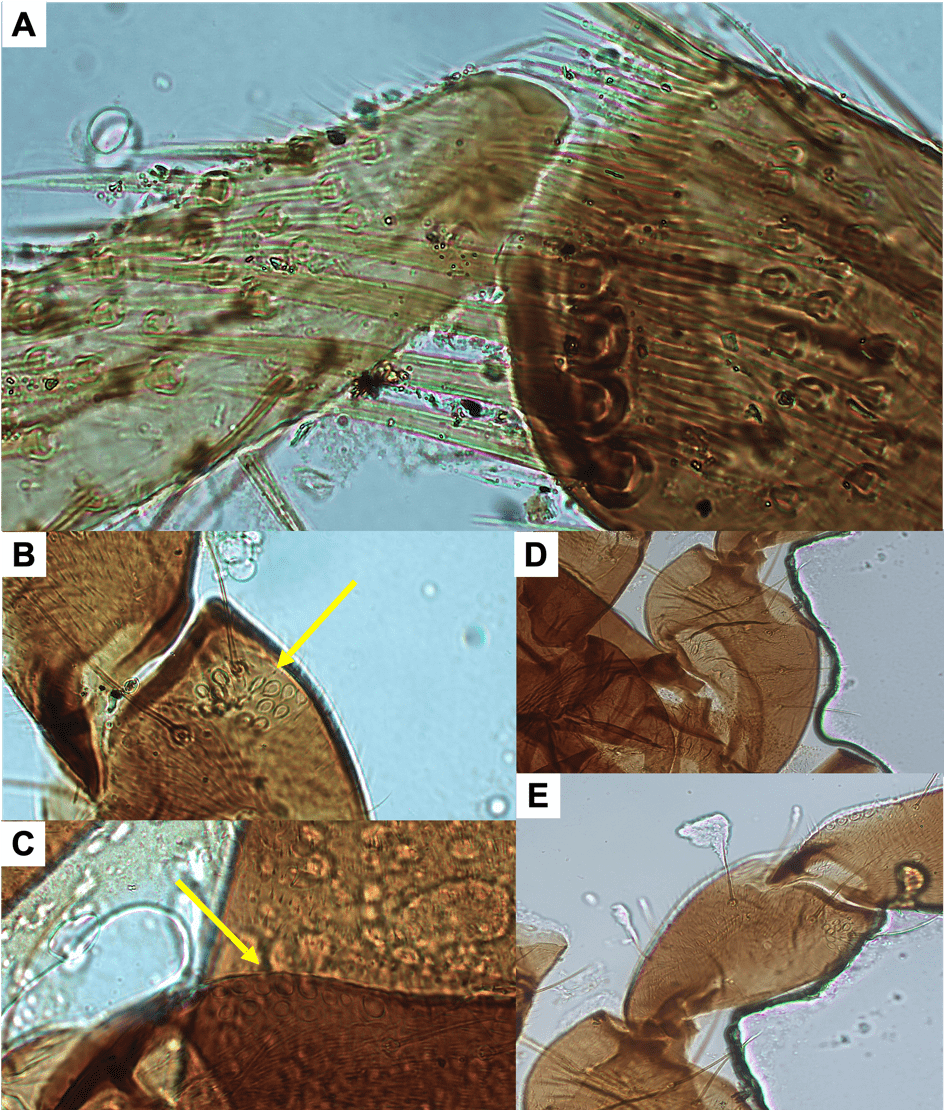


***Figure S24***. Claws of *Leptoconops communis* sp. nov. (MS).


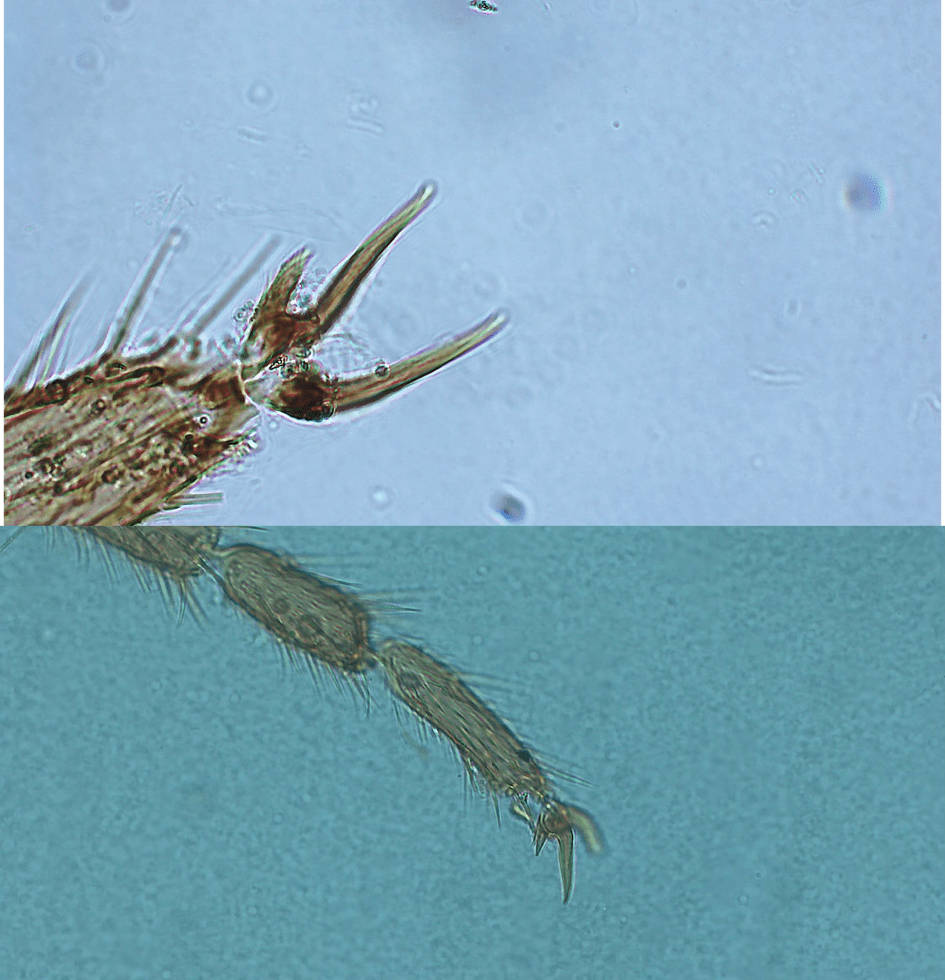


***Fig S25***. Wing and abdomen of *Leptoconops communis* sp. nov. A, B. Wing (FS), C. Tergites (FS), D. Sternites (FS), E. Lamellae (MS), F. Ventral view of anal armature (FS), G. Sternite VIII and extremities of ventral plate (MS), H. Main spermathecae (MS).


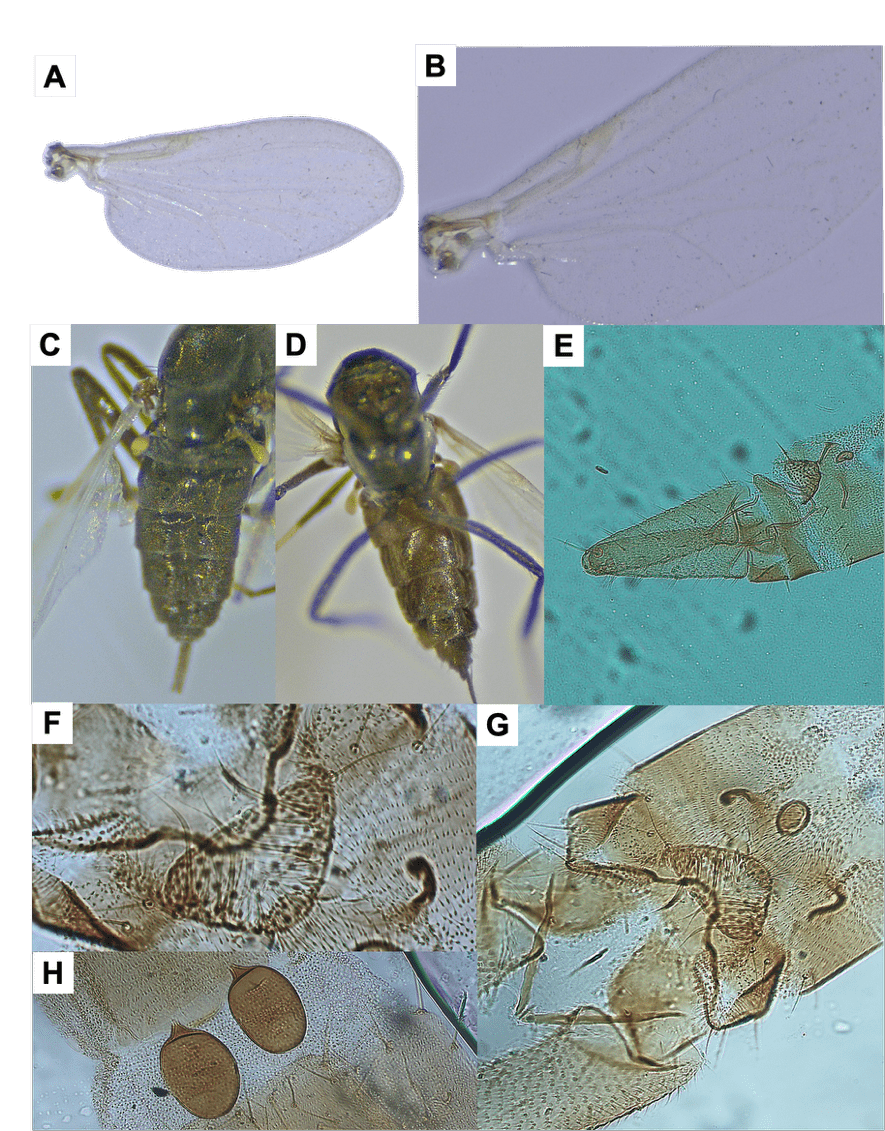


***Table S1***. Description of the main taxonomic morphological features and measurements of *Leptoconops bidentatus*, *Leptoconops irritans*, *Leptoconops bezzii* and *Leptoconops noei*. *Leptoconops noei and Leptoconops bezzii* data were obtained from Italian specimens. *Colour of fore legs seem to be variable between Italian and Spanish specimens.

|  |  | | *Species* | | | |
| --- | --- | --- | --- | --- | --- | --- |
| **Morphological features** | | | ***Leptoconops bidentatus*** | ***Leptoconops irritans*** | ***Leptoconops bezzii*** | ***Leptoconops noei*** |
| **BODY** | | ***Size (mm ± SD)*** | 1.58 ± 0.14 | 1.75 ± 0.13 | 2.01 ± 0.20 | - |
| **THORAX** | | ***Mesonotum and pleuron*** | Shiny dark brown | Dark, dull, pruinose with  metallic tones | Dark, dull, pruinose  with metallic tones | Dark, dull, pruinose  with metallic tones |
|  |  | ***Scutellum setae.*** | 4 setae  2 setae on each side. | 4 setae  2 setae on each side. | - | 6 setae  3 setae on each side. |
|  |  | ***Halters*** | Clean white | Clean white | Dirty white | Dirty white |
|  |  | ***Legs fresh.*** | Evenly light brown  coxa dark and femur, tibia and tarsi pale. | Femur and tibia evenly dark. Base almost entire length except apical tips. of 1^st^ tarsi of the mid and hind legs pale. Base 3/4. of the 2^nd^ tarsi of the fore and midlegs occasionally pale colour | Femur and tibia evenly dark. 1^st^ tarsi of fore legs evenly dark. 1^st^ tarsi mid and hind legs. and base of 2^nd^ tarsi of hindlegs entirely pale. Base 3/4 length. of 2^nd^ tarsi of hind legs pale colour | Femur and tibia evenly dark. * Base 3/4 length. of the 1^st^ tarsi of fore legs pale. 1^st^ tarsi mid and hind legs. and base of 2^nd^ tarsi of hindlegs entirely pale. Base 3/4 length. of 2^nd^ tarsi of hind legs pale colour |
|  |  | ***Tarsal segments apical spines.*** | 1^st^ and 2^nd^ | 1^st^ and 2^nd^  variable on 3^rd^. | 1^st^ and 2^nd^  sometimes on 3^rd^. | 1^st^ and 2^nd^  sometimes on 3^rd^. |
|  |  | ***Tibial comb nº and shape.*** | 4 spines  Outers shorter than inners | 4 spines  Outers markedly smaller than inners | 4 spines  Outers markedly larger than inners | 4 spines  Outers markedly larger than inners |
|  |  | ***Claws*** | Asymmetrical; one side bears two basal teeth one large and other smaller., while the other typically has one tooth and one hair, creating a bidentate appearance | Equal and simple with a bristle on each claw. Shape with smooth gradual curvature | Claws with a basal tooth and a bristle. Strongly curved at the base | Claws with a moderate basal tooth and a bristle. Strongly curved at the base |
|  |  | ***Wing length x width (mm ± SD)*** | ̶ | 0.97 ± 0.02 x 0.40 ± 0.01 | - | 1.14 ± 0.04 x 0.43 ± 0.01 |
| **HEAD** | | ***Interocular space (µm ± SD)*** | 100.40 ± 16.08 | 82.89 ± 18.30 | 112 | 96.72 ± 10.18 |
|  |  | ***Proboscis fresh specimens.*** | Short proboscis  150.30 **±** 7.50 | Long proboscis  350.78 **±** 21.13 | Proboscis as long as  head or slightly shorter | Proboscis as long as  head or slightly shorter |
|  |  | ***Head length x width (µm ± SD)*** | 353.86 ± 16.21 x 311 ± 7.30 | 426.99 ± 8.24 x 423.6 ± 5.84 |  | 376.94 ± 6.33 |
|  |  | ***Colour***  ***Frons pubescence*** | Brown to dark  Little spiculated | Brown to dark  Moderately spiculated | Brown to dark  Moderately spiculated | Brown to dark  Moderately spiculated |
|  |  | ***Vertex nº setae.*** | 10-12 | 10-12 | 12 | 12 |
|  |  | ***Interocular space nº setae.*** | 1 pair of setae on each side plus 1-2 small holes on each side | 2 setae  one on each side. | 2 small holes on each side | 1 pair of setae on  each side sometimes with extra holes. |
|  |  | ***Fronto-clypeus nº setae.*** | 4 | 4 | 4 | 4 |
|  |  | ***Fronto-clypeus disposal.*** | Trapezoidal | Trapezoidal | Trapezoidal | Trapezoidal  Anterior setae separated by a short distance |
|  |  | ***Teeth nº mandible.*** | 18 knife shaped. | 24-26 | 19 | 17-19 |
|  |  | ***Teeth nº lacinia.*** | 12 | 16-18  distal teeth larger than basal. | 19 | 17-19 |
|  |  | ***Palpal ratio*** | 2.49 ± 0.24 | 2.86 ± 0.17 | 2.51 | 1.93 ± 0.22 |
|  |  | ***Shape 3^rd^ and 4^th^ palpus.***  ***(µm ± SD)***  ***Length and width 3^rd^ and 4^th^ palpus.***  ***(µm ± SD)*** | 3^rd^: small  4^th^: thick with clubbed termination  3^rd^ darker than 4^th^ segment.  68.59 ± 4.09 x 27.46 ± 3.3  74.30 ± 4.5 x 17.47 ± 2.45 | 3^rd^: elongate  4^th^: thin and slender  3^rd^ and 4^th^ with similar colour.  135.78 ± 7.57 x 51.46 ± 1.69  146.14 ± 16.2 x 15.12 ± 2.81 | 3^rd^: elongate  4^th^: cylindrical  3^rd^ and 4^th^ with similar colour.  86.26 ± 4.36 x 34.3 ± 0.19  84.93 ± 4.83 x 13.62 ± 0.81 | 3^rd:^ incrassate  4^th^: cylindrical  3^rd^ and 4^th^ with similar colour.  87.53 ± 3.98  - |
|  |  | ***Sensory pits 3^rd^ palpus.*** | 20-25 sensilla in 3-4 irregular rows < 50% of the surface covered | 45-50 sensilla in 3-4 irregular rows  75% of the surface covered | 30-40 sensilla in 6 irregular rows | 30-40 sensilla in 6-8 irregular rows  50% of the surface covered |
|  |  | ***Antennal ratio*** | 0.69 | 0.73 | 0.73 | 0.65 |
|  |  | ***Antennal segments chaetotaxy*** | Scape/pedicel: 4 setae  I: 5-7 setae  II-XI: one pair of well-separated blunt hyaline sensilla III-XIII. on each flagellomere plus a single short hyaline sensilla at least on VIII-XIV.  II-XI: with a variable number of ordinary setae on each flagellomere  I-XI variable on VII-IX: a single rarely 2, long seta on each flagellomere  XII: Setose. 3-5 long setae | Scape/pedicel: 3-4 setae  I: 5-7 setae  I-XI: one pair of well-separated blunt hyaline sensilla strongly curved at the base III-XIII. on each flagellomere plus a single slender hyaline sensilla variable presence.  II-XI: with a high number 16-20. of ordinary setae on each flagellomere mainly distributed on one face  I- XI: With 1 distal segment to 3 basal segments. long setae on each flagellomere  XII: Setose. 5-6 long setae | Scape/pedicel: 3-6 setae  I: 6 setae  II-IV: 1 long seta  I-XI: one pair of well-separated blunt hyaline sensilla strongly curved at the base on each flagellomere, one row of 8-10 hyaline sensilla on external side, two irregular rows of 16-20 hyaline sensilla on internal side.  XII: slightly setose, 3-4 long setae | One pair of blunt trichoid sensilla III-XIII. in each flagellomere. Distance between sensilla variable usually closer in distant segments. Long, curved and thin sometimes 90º curved. Antennal segments little setose. Last segment slightly setose. |
|  |  | ***Antennal segments shape.*** | II-IV longitudinally flattened  V -XI spherical  XII as long as preceded two together | II-VI longitudinally flattened  VII subspherical  VIII-XI spherical  XII as long as preceded two together | II-IV longitudinally flattened  V-XI globular  XII length as 2-2.5 preceded segments | II-III longitudinally flattened  IV variable, subspherical.  V - XI globular  XII length as 2-2.5 preceded segments |
|  |  | ***Antenna length III- XIV (µm)*** | 322.68 | 392.35 | 398.38 | 406.01 |
| **ABDOMEN** | | ***Cerci length x width (µm ± SD)*** | 270.26 ± 27.98 x 62.04 ± 17.06 | 247. 28 ± 17.96 x 78.96 ± 4.27 | 209.78 ± 2.01 x 63.33 ± 2.33 | 207.42 ± 7.03 x 74.40 ± 4.69 |
|  |  | ***Main spermathecae length x width (µm ± SD)*** | 54.71 ± 4.11 x 33.66 ± 1.55 | 61.97 ± 0.80 x 33.49 ± 1.30 | 57 x 31 | 68.05 ± 2.11 x 34.93 ± 3.99 |
|  |  | ***Main spermathecae*** | Subequal. Oval-shaped and moderately sclerotized | Sausage shaped, relatively large and moderately sclerotized | Oval-shaped, relatively large and moderately sclerotized | Sausage shaped, large, moderately sclerotized and neck well-developed. Short pubescence on the terminal end |
|  |  | ***Third spermatheca*** | Present | Present | Present | Present |
|  |  | ***Anal cone*** | Conspicuous  4-8 large bristles mean: 5.6. | Wide and conspicuous  4 large bristles | 4 large bristles | 4 large bristles |
|  |  | ***Tergites***  ***Colour*** | Light brown darker in the base. | Yellowish grey isabelline coloured. | Brown | Brown |
|  |  | ***Armature anal cone*** | V-shaped  Central join inconspicuous | V-shaped | V-shaped | V-shaped  Central join with a conspicuous protuberance |

***Table S2.*** Antennal flagellomere measurements of the new described *Leptoconops* species.

| **ANTENNAL FLAGELLOMERES MEASUREMENTS IN (µm ± SD, N=7)** | | | | |
| --- | --- | --- | --- | --- |
| **FLAGELLOMERE** | **SPECIES** | | | |
|  | ***Leptoconops nigrithorax*** | ***Leptoconops triangularis*** | ***Leptoconops pseudoirritans*** | ***Leptoconops communis*** |
| I | 41.71 ± 6.52 | 62.64 ± 3.28 | 38.80 ± 5.52 | 53.35 ± 5.88 |
| II | 25.82 ± 3.82 | 33.09 ± 3.37 | 24.55 ± 3.42 | 32.55 ± 3.57 |
| III | 24.69 ± 2.96 | 29.51 ± 2.13 | 23.56 ± 2.19 | 28.42 ± 5.17 |
| IV | 26.11 ± 3.83 | 30.40 ± 1.98 | 23.11 ± 2.25 | 27.82 ± 2.51 |
| V | 25.82 ± 2.77 | 29.38 ± 4.93 | 23.67 ± 1.94 | 29.52 ± 1.79 |
| VI | 26.81 ± 3.32 | 32.91 ± 1.25 | 23.37 ± 2.64 | 32.10 ± 2.39 |
| VII | 27.02 ± 2.57 | 34.71 ± 1.59 | 23.53 ± 1.81 | 33.28 ± 1.78 |
| VIII | 27.29 ± 2.31 | 35.58 ± 3.11 | 23.82 ± 2.01 | 33.28 ± 3.19 |
| IX | 27.83 ± 1.53 | 35.21 ± 1.57 | 23.68 ± 1.29 | 34.20 ± 3.19 |
| X | 28.45 ± 3.25 | 35.81 ± 1.59 | 23.19 ± 1.57 | 33.36 ± 2.89 |
| XI | 29.06 ± 3.52 | 35.33 ± 1.93 | 23.57 ± 1.28 | 33.82 ± 2.67 |
| XII | 75.75 ± 2.84 | 91.05 ± 2.88 | 63.31 ± 2.7 | 81.31 ± 6.76 |

***Table S3.*** Legs segments measurements of the new described *Leptoconops* species.

| Legs segments measurements in (µm ± SD, N = 7) | | | | | |
| --- | --- | --- | --- | --- | --- |
| Leg | **SEGMENT** | **SPECIES** | | | |
|  |  | ***Leptoconops nigrithorax*** | ***Leptoconops triangularis*** | ***Leptoconops pseudoirritans*** | ***Leptoconops communis*** |
| Fore | Trochanter | 120.70 ± 17.46 | 108.28 ± 9.39 | 98.86 ± 12.91 | 114.37 ± 14.22 |
|  | Femur | 435.62 ± 15.24 | 458.47 ± 18.24 | 412.84 ± 25.80 | 464.01 ± 27.94 |
|  | Tibia | 459.20 ± 20.80 | 452.55 ± 25.57 | 399.09 ± 17.77 | 465.12 ± 35.75 |
|  | 1^st^ Tarsomere | 204.25 ± 11.28 | 220.93 ± 14.59 | 185.12 ± 8.56 | 225.90 ± 22.32 |
|  | 2^nd^ Tarsomere | 98.22 ± 4.05 | 100.83 ± 5.61 | 90.48 ± 3.40 | 104.48 ± 11.20 |
|  | 3^rd^ Tarsomere | 73.09 ± 3.92 | 79.69 ± 2.33 | 71.98 ± 8.49 | 81.63 ± 5.64 |
|  | 4^th^ Tarsomere | 53.86 ± 3.86 | 54.67 ± 3.14 | 48.25 ± 4.45 | 61.94 ± 3.76 |
|  | 5^th^ Tarsomere | 66.10 ± 5.58 | 70.41 ± 4.52 | 61.75 ± 3.90 | 75.09 ± 2.83 |
| Mid | Trochanter | 87.82 ± 9.19 | 103.20 ± 8.10 | 89.07 ± 17.40 | 92.26 ± 6.84 |
|  | Femur | 552.71 ± 19.35 | 509.65 ± 52.61 | 436 ± 32.70 | 573.62 ± 12.55 |
|  | Tibia | 468.30 ± 24.04 | 460.29 ± 35.74 | 398.93 ± 17.21 | 506.65 ± 18.24 |
|  | 1^st^ Tarsomere | 246.87 ± 16.32 | 233.95 ± 30.40 | 186.28 ± 9.95 | 257.80 ± 6.11 |
|  | 2^nd^ Tarsomere | 120.83 ± 24.81 | 117.83 ± 30.83 | 93.97 ± 4.54 | 121.74 ± 9.13 |
|  | 3^rd^ Tarsomere | 82 ± 10.93 | 81.76 ± 11.53 | 70.14 ± 2.62 | 84.79 ± 5.14 |
|  | 4^th^ Tarsomere | 51.74 ± 3.18 | 53.16 ± 6.38 | 46.69 ± 5.79 | 61.08 ± 1.29 |
|  | 5^th^ Tarsomere | 70.83 ± 5.01 | 68.92 ± 2.25 | 62.81 ± 3.92 | 76.08 ± 2.09 |
| Hind | Trochanter | 103.30 ± 8.58 | 108.38 ± 16.95 | 92.57 ± 12.05 | 106.75 ± 9.88 |
|  | Femur | 547.80 ± 26.14 | 595.61 ± 22.44 | 501.73 ± 36.19 | 539.24 ± 11.51 |
|  | Tibia | 499.13 ± 32.76 | 558.97 ± 22 | 458.44 ± 30.67 | 503.11 ± 8.21 |
|  | 1^st^ Tarsomere | 291.15 ± 19.94 | 339.52 ± 29.01 | 257.68 ± 27.85 | 296.38 ± 20.45 |
|  | 2^nd^ Tarsomere | 186.35 ± 15.15 | 209.57 ± 12.69 | 165.38 ± 18.02 | 181.15 ± 13.66 |
|  | 3^rd^ Tarsomere | 104.23 ± 7.66 | 117.66 ± 8.75 | 91.09 ± 11.90 | 99.57 ± 2.24 |
|  | 4^th^ Tarsomere | 55.93 ± 4.76 | 71.19 ± 5.04 | 56.81 ± 5.87 | 63.28 ± 0.92 |
|  | 5^th^ Tarsomere | 77.65 ± 5.25 | 79.92 ± 3.54 | 67.51 ± 3.63 | 77.18 ± 4.06 |
